# The Novel Atypical Dopamine Uptake Inhibitor *(S)*-CE-123 Partially Reverses the Effort-Related Effects of the Dopamine Depleting Agent Tetrabenazine and Increases Progressive Ratio Responding

**DOI:** 10.3389/fphar.2019.00682

**Published:** 2019-06-28

**Authors:** Renee A. Rotolo, Vladimir Dragacevic, Predrag Kalaba, Ernst Urban, Martin Zehl, Alexander Roller, Judith Wackerlig, Thierry Langer, Marco Pistis, Maria Antonietta De Luca, Francesca Caria, Rebecca Schwartz, Rose E. Presby, Jen-Hau Yang, Shanna Samels, Merce Correa, Gert Lubec, John D. Salamone

**Affiliations:** ^1^Department of Psychological Sciences, University of Connecticut, Storrs, CT, United States; ^2^Department of Pharmaceutical Chemistry, Faculty of Life Sciences, University of Vienna, Vienna, Austria; ^3^Department of Analytical Chemistry, Faculty of Chemistry, University of Vienna, Vienna, Austria; ^4^X-ray Structure Analysis Centre, Faculty of Chemistry, University of Vienna, Vienna, Austria; ^5^Department of Biomedical Sciences, University of Cagliari, National Institute of Neuroscience (INN), Cagliari, Italy; ^6^Àrea de Psicobiologia, Universitat Jaume I, Castelló, Spain; ^7^Department of Neuroproteomics, Paracelsus Medical University, Salzburg, Austria

**Keywords:** dopamine, transport, synthesis, motivation, depression, fatigue, anergia, modafinil

## Abstract

Animal studies of effort-based choice behavior are being used to model effort-related motivational dysfunctions in humans. With these procedures, animals are offered a choice between high-effort instrumental actions leading to highly valued reinforcers vs. low effort/low reward options. Several previous studies have shown that dopamine (DA) uptake inhibitors, including GBR12909, lisdexamfetamine, methylphenidate, and PRX-14040, can reverse the effort-related effects of the vesicular monoamine transport blocker tetrabenazine, which inhibits DA storage. Because many drugs that block DA transport act as major stimulants that also release DA, and produce a number of undesirable side effects, there is a need to develop and characterize novel atypical DA transport inhibitors. *(**S**)*-CE-123 (*(**S**)*-*5*-((benzhydrylsulfinyl) methyl)thiazole) is a recently developed analog of modafinil with the biochemical characteristics of an atypical DA transport blocker. The present paper describes the enantioselective synthesis and initial chemical characterization of *(**S**)*-CE-123, as well as behavioral experiments involving effort-based choice and microdialysis studies of extracellular DA. Rats were assessed using the fixed ratio 5/chow feeding choice test. Tetrabenazine (1.0 mg/kg) shifted choice behavior, decreasing lever pressing and increasing chow intake. *(**S**)*-CE-123 was coadministered at doses ranging from 6.0 to 24.0 mg/kg, and the highest dose partially but significantly reversed the effects of tetrabenazine, although this dose had no effect on fixed ratio responding when administered alone. Additional experiments showed that *(**S**)*-CE-123 significantly increased lever pressing on a progressive ratio/chow feeding choice task and that the effective dose (24.0 mg/kg) increased extracellular DA in nucleus accumbens core. In summary, *(**S**)*-CE-123 has the behavioral and neurochemical profile of a compound that can block DA transport, reverse the effort-related effects of tetrabenazine, and increase selection of high-effort progressive ratio responding. This suggests that *(**S**)*-CE-123 or a similar compound could be useful as a treatment for effort-related motivational dysfunction in humans.

## Introduction

Motivational symptoms such as fatigue, anergia, and psychomotor slowing are seen in depression, Parkinson’s disease, and other disorders. These symptoms are often debilitating and can severely limit long-term functional outcomes (Demyttenaere et al., 2005; Salamone et al., 2006; Friedman et al., 2007; Fava et al., 2014; Rothschild et al., 2014; Chong et al., 2015; Salamone et al., 2016a; Salamone et al., 2016b; Salamone et al., 2016c; Salamone et al., 2017). Moreover, motivational symptoms can be highly resistant to treatment with common antidepressants such as serotonin transporter (SERT) blockers (Cooper et al., 2014; Fava et al., 2014; Rothschild et al., 2014). The catecholamine uptake inhibitor bupropion has shown some success in treating motivational symptoms in depressed people (Pae et al., 2007; Papakostas et al., 2006; Cooper et al., 2014), and reports indicate that treatment with drugs that inhibit dopamine transporter (DAT), such as d-amphetamine and methylphenidate, can improve motivational function (Stotz et al., 1999). However, psychomotor stimulants that block DAT also can have undesirable effects, such as abuse liability and induction of psychotic symptoms. For these reasons, it is important to develop and assess drugs that are highly selective for DAT but show atypical neurochemical characteristics that may lower the side effect profile.

**Figure 1:**
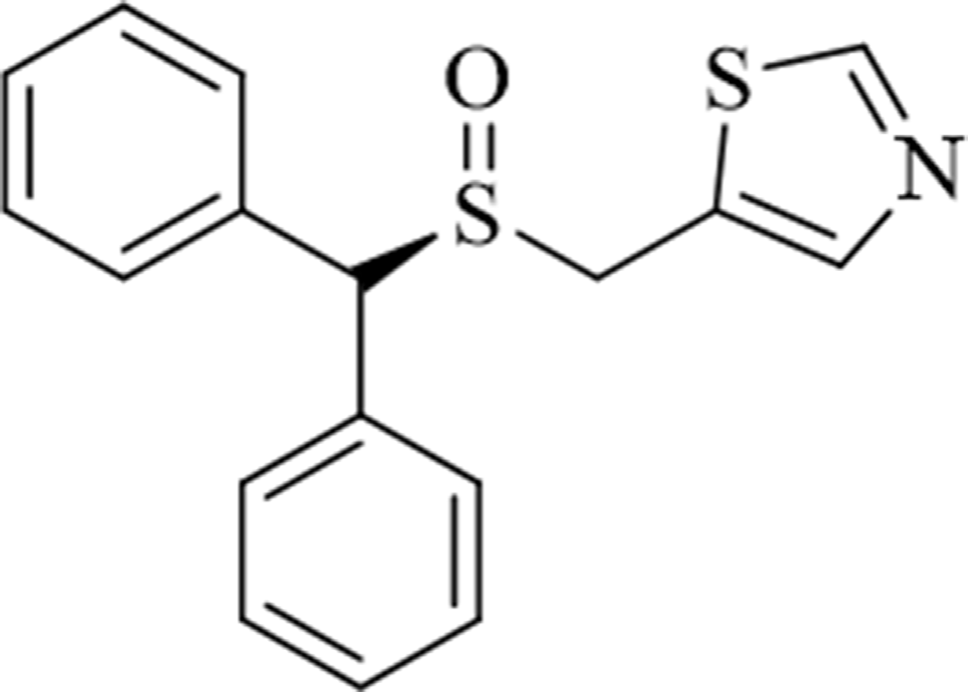
Structure of the novel atypical dopamine reuptake inhibitor (*S*)-CE-123 ((*S*)-5-((benzhydrylsylfinyl)methyl)thiazole).

A recently synthesized and chromatographically separated analog of modafinil, *(**S**)*-CE-123 (**Figure 1**), is a highly selective atypical inhibitor of DAT that has been shown to enhance cognitive flexibility and reduce impulsivity in rats (Nikiforuk et al., 2017). The present paper describes the enantioselective synthesis and initial characterization of *(**S**)*-CE-123. In addition, *(**S**)*-CE-123 was assessed for its effects on effort-related aspects of motivation in rats. These behavioral pharmacology studies evaluated the motivational effects of *(**S**)*-CE-123 in rats using well-characterized tests of effort-based choice behavior, the concurrent fixed ratio 5 (FR5)/chow feeding choice task, and the concurrent progressive ratio (PROG)/chow feeding choice task. Effort-related choice is studied using procedures that offer high-effort options leading to highly valued reinforcers vs. low-effort/low-reward options (Salamone and Correa, 2012; Salamone et al., 2016a; Salamone et al., 2016b; Salamone et al., 2016c; Hart et al., 2017; Salamone et al., 2017; Salamone et al., 2018). Considerable research has implicated dopamine (DA) transmission, particularly in nucleus accumbens core, in the regulation of effort-based choice; animals with impaired DA transmission (i.e., DA antagonism or depletion) show a shift from the high-effort option to the low-effort option, while enhancement of DA transmission reverses those effects (Sokolowski and Salamone, 1998; Salamone et al., 2002; Salamone et al., 2007; Floresco et al., 2008; Farrar et al., 2010; Mai et al., 2012; Hosking et al., 2015; Salamone et al., 2016a; Salamone et al., 2016b; Salamone et al., 2016c). It has been suggested that animal models employing tests of effort-based decisión-making can be used to study functions that are related to aspects of human motivational dysfunction (Salamone et al., 2006; Salamone et al., 2016a; Salamone et al., 2016b; Salamone et al., 2016c; Salamone et al., 2018). This strategy has been validated by clinical research showing that patients with major depression, Parkinson’s disease, and other disorders show a low-effort bias when tested on effort-related choice procedures (Treadway et al., 2012; Yang et al., 2014; Chong et al., 2015; Barch et al., 2017).

Studies have shown that a low-effort bias can be induced in rats by several conditions associated with depressive symptoms, including stress (Shafiei et al., 2012; Bryce and Floresco, 2016), inflammatory challenge (Nunes et al., 2014; Yohn et al., 2016e), withdrawal from methamphetamine (Hart et al., 2018), and injections of the vesicular monoamine transporter type-2 inhibitor tetrabenazine (TBZ; Nunes et al., 2013; Randall et al., 2014; Yohn et al., 2015a; Yohn et al., 2015b). TBZ induces depressive symptoms in people (Frank, 2009; Frank, 2010; Guay, 2010) and has been used in classical animal models of depression (Tadano et al., 2000; Wang et al., 2010). By virtue of inhibiting vesicular monoamine transporter type-2, TBZ blocks DA storage, depletes brain DA, and reduces postsynaptic DA receptor signaling (Nunes et al., 2013). Across a variety of behavioral tests, TBZ shifts choice behavior and induces a low-effort bias, and the reallocation of behavior from high- to low-effort options that is produced by TBZ is not due to alterations in food preference or hedonic taste reactivity, reduced apetite, or impairments in reference memory (Randall et al., 2012; Nunes et al., 2013; Randall et al., 2014; Pardo et al., 2015; Yohn et al., 2015a). On operant behavior tests that give animals the choice between lever pressing on a FR5 or PROG schedule for a preferred food vs. approaching and consuming a less preferred lab chow (Nunes et al., 2013; Randall et al., 2014; Yohn et al., 2016a; Yohn et al., 2016b; Yohn et al., 2016c), TBZ reliably decreases working for food by lever pressing but actually increases consumption of the concurrently available chow. TBZ in this dose range does not reduce consumption of either type of food that is used in these studies nor does it affect preference as measured in free-feeding preference tests (Nunes et al., 2013). Also, the effects of TBZ on operant choice procedures differ substantially from the effects of reinforcer devaluation, as well as appetite suppressant drugs (Randall et al., 2012; Randall et al., 2014). These effects of TBZ are not reversed by the SERT blockers fluoxetine and citalopram, or by the norepinephrine transporter (NET) inhbitor desipramine (Yohn et al., 2016a; Yohn et al., 2016b), but they are attenuated by several drugs that block DAT, including bupropion, GBR12909, lisdexamfetamine, methylphenidate, and modafinil (Nunes et al., 2013; Randall et al., 2014; Salamone et al., 2016a; Salamone et al., 2016b; Yohn et al., 2016a; Yohn et al., 2016b; Yohn et al., 2016c). Drugs that inhibit DAT, such as bupropion, lisdexamfetamine, and PRX-14040, also have been shown to increase the motivation to work for food reinforcers in more highly demanding behavioral tasks, such as the PROG/chow feeding choice task (Randall et al., 2015; Yohn et al., 2016b; Yohn et al., 2016c; Yohn et al., 2016d). In view of these results, the present studies investigated the ability of *(**S**)*-CE-123 to attenuate the effort-related effects of TBZ in rats tested on the concurrent FR5/chow feeding choice task and to enhance exertion of effort in rats assessed with the PROG/chow feeding choice task. An additional experiment studied the effects of the behaviorally effective dose of *(**S**)*-CE-123 (24.0 mg/kg) on extracelular DA in nucleus accumbens using microdialysis methods.

## Materials and Methods

### Synthesis of *(S)*-CE-123

#### Synthesis of 5-(Chloromethyl)Thiazole Hydrochloride

**Figure:**
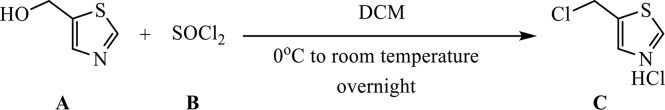


**A** (0.63 **g**, 5.5 mmol) was dissolved in 20 ml of dichloromethane (DCM) and dunked in an ice bath. **B** (0.40 ml, 5.5 mmol) was gradually dripped in, and the reaction was allowed to proceed overnight. The reaction mixture was concentrated *in vacuo*, and the obtained substance was dried in high vacuum to remove thionyl chloride and water excess (the product is highly hygroscopic) to afford 0.93 g of product **C** as yellow powder (yield >95%, quantitative).

#### Synthesis of [(Diphenylmethyl)Sulfanyl]Methanimideamide Hydrobromide

**Figure:**
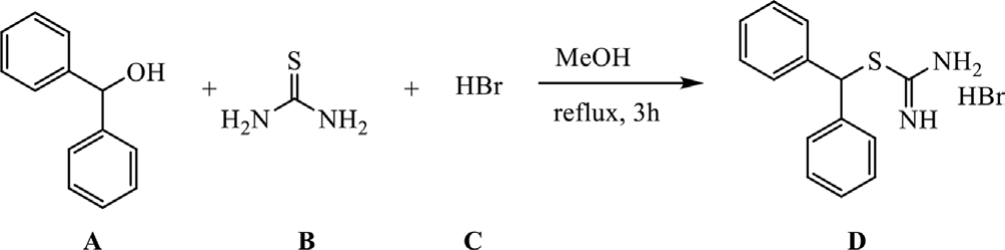


**A** (1.29 g, 7 mmol) was dissolved in 30 ml of methanol (MeOH) in a 100-ml three-necked round-bottomed flask, **B** (0.61 g, 8 mmol) was added, and the reaction mixture was refluxed (external temperature 87–90°C) for 30 min. **C** (3.64 ml, 32.2 mmol) was added in a dropwise manner over the course of half an hour, and the reaction mixture was further refluxed for an additional 3 h.

The reaction mixture was cooled down to room temperature, MeOH was evaporated, and the remaining substance was suspended in 30 ml of DCM, stirred for half an hour, and filtered through a Büchner funnel to remove unreacted **A**. The solid residue was washed with additional 30 ml of DCM and subsequently suspended in 30 ml of water, stirred for half an hour, and filtered through a Büchner funnel to remove unreacted **B**. The residue was washed with 30 ml of water and dried to give 1.62 g of product D as fine, white powder (yield 71%).

#### Synthesis of 5-((Benzhydrylthio)Methyl)Thiazole

**Figure:**
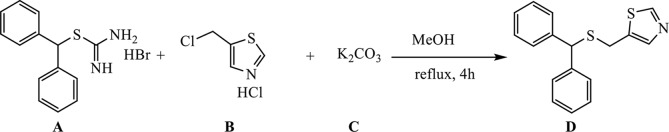


**A** (1.62 g, 5 mmol) was dissolved in 30 ml of MeOH, and **B** (0.93 g, 5.5 mmol) was added and dissolved, upon which **C** (3.45 g, 25 mmol) was introduced, and the reaction mixture was refluxed for 4 h (external temperature was 87–90°C).

The hot reaction mixture was filtered through a Büchner funnel to remove the majority of K_2_CO_3_, and methanol was evaporated under reduced pressure. The remaining substance was suspended in 50 ml of water, and reaction products were extracted with ethyl acetate (3 × 75 ml). The extracts were pooled, dried over anhydrous Na_2_SO_4_, filtered and condensed under reduced pressure to afford an oily mixture that was purified by column chromatography on silica gel (2.5% solution of MeOH in DCM was used as eluent), and recrystallized from petroleum ether to yield 1.25 g of the desired product **D** as white crystals (yield 84%).

#### Synthesis of ***(S)***-**5**-((Benzhydrylsulfinyl)Methyl)Thiazole (***(S)***-CE-123)

**Figure:**
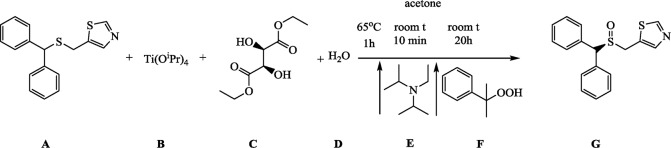


**A** (1.25g, 4.25 mmol) was dissolved in 25 ml of acetone in a 100-ml round-bottomed flask. **B** (0.37 ml, 1.25 mmol), **C** (0.43 ml, 2.5 mmol), and **D** (11.25 µL, 0.625 mmol) were added to the mixture, which was stirred for 5 min to achieve homogeneity at the room temperature and then refluxed for 1 h at 65°C (external temperature). The mixture was given 15 min to cool down to room temperature, **E** (0.15 ml, 0.85 mmol) was added, and after 10 min of stirring, **F** (0.78 ml, 4.25 mmol) was added, and the reaction was allowed to proceed for 20 h.

Acetone was evaporated from the mixture, and the oily orange–brown residue was purified by column chromatography on silica gel (2.5% solution of methanol in DCM was used as the mobile phase) to give 0.99 g of the final product **G**, as white solid (yield 74%).

### *In Vivo* Behavioral Pharmacology

#### Animals

For the behavioral pharmacology experiments, adult male Sprague-Dawley rats (Envigo Sprague-Dawley, Indianapolis, IN, USA; weights 275–299 g upon arrival) were pair-housed in a colony maintained at 23°C, with a 12-h light/dark cycle (lights on 07:00). Rats were food deprived to 85% of their free-feeding body weight for operant training and allowed modest growth throughout the experiment. Water was available *ad libitum* in the home cages. Animal protocols were approved by the University of Connecticut Institutional Animal Care and Use Committee, and the studies were conducted according to National Institutes of Health guidelines. For the *in vivo* microdialysis experiment, adult male Sprague-Dawley rats (Harlan Italy; weights 275–300 g upon arrival) were used. Rats were housed four per cage, in standard plastic cages with wood chip bedding, maintained at 22 ± 2°C and 60% humidity with a 12-h light/dark cycle (lights on 07:00). Water and standard laboratory rodent chow (Mucedola, Settimo Milanese, Italy) were provided *ad libitum* in the home cage. All animal experiments were carried out in accordance with the Guidelines for the Care and Use of Mammals in Neuroscience and Behavioral Research according to Italian (D.L. 116/92 and 152/06) and European Council (609/86 and 63/2010) directives and in compliance with the approved animal policies by the Ethical Committee for Animal Experiments (CESA, University of Cagliari) and the Italian Ministry of Health (Aut. N. 162/2016- PR). All efforts were taken to minimize pain and suffering and to reduce the number of animals used.

#### Behavioral Procedures

##### FR5/Chow Feeding Choice Task

Behavioral sessions were conducted in operant chambers (28 × 23 × 23 cm^3^; Med Associates, Fairfax, VT) with 30-min sessions 5 days/week. Rats were initially trained to lever press on a continuous reinforcement FR1 schedule (high-carbohydrate 45-mg pellets, Bio-Serv, Frenchtown, NJ) and then shifted to the FR5 schedule. After 5 weeks of training on the FR5 schedule, chow was introduced. Weighed amounts of laboratory chow (Laboratory Diet, 5P00 Prolab RMH 3000, Purina Mills, St. Louis, MO; typically 15–20 g) were concurrently available on the floor of the chamber during the FR5/chow feeding choice task sessions. At the start of each session, it was confirmed that the pieces of weighed chow were larger than the spaces between the bars that make up the floor of the chamber, so they could not fall through. At the end of each 30-min session, rats were immediately removed from the chambers, number of lever presses was recorded, and the amount of chow consumed was determined by weighing the remaining food (including spillage from a tray beneath the floor of the chamber). Rats were trained on the FR5/chow feeding choice procedure for 5 weeks, after which drug testing began. On baseline and drug treatment days, rats consumed all of the operant pellets that were delivered during each session.

##### PROG/Chow Feeding Choice Task

Behavioral sessions were conducted in operant chambers with 30-min sessions 5 days/week. Rats were initially trained to lever press on a continuous reinforcement FR1 schedule (high-carbohydrate 45-mg pellets, Bio-Serv) and then shifted to the PROG schedule (Randall et al., 2012; Randall et al., 2014; Randall et al., 2015). For PROG sessions, the ratio started at FR1 and was increased by one additional response every time 15 reinforcements were obtained (FR1 × 15, FR2 × 15, etc.). A “time-out” feature deactivated the response lever for the rest of the session whenever 2 min elapsed without a completed ratio. After 9 weeks of training on the PROG schedule, chow was introduced. Weighed amounts of laboratory chow (Laboratory Diet, 5P00 Prolab RMH 3000, Purina Mills; typically 15–20 g) were concurrently available on the floor of the chamber during the PROG/chow feeding choice task sessions. At the end of each 30-min session, rats were immediately removed from the chambers, number of lever presses was recorded, and the amount of chow consumed was determined by weighing the remaining food (including spillage from a tray beneath the floor of the chamber). Rats were trained on the PROG/chow feeding choice procedure for 5 weeks, after which drug testing began. On baseline and drug treatment days, rats consumed all of the operant pellets that were delivered during each session.

#### Drug Treatments and Dose Selection

*(**S**)*-CE-123 (*(**S**)*-*5*-((benzhydrylsulfinyl)methyl)thiazole) was obtained from the Lubec Laboratory (University of Vienna, Austria) and dissolved in dimethyl sulfoxide (DMSO) (10%), Tween 80 (15%), and 0.9% saline (75%). The DMSO/Tween 80/saline solution was administered as the vehicle control. TBZ (9,10-dimethoxy-3-(2-methylpropyl)-1,3,4,6,7, 11b hexahydrobenzo[a]quinolizin-2-one) was obtained from Tocris Bioscience (Ellisville, MO) and was dissolved in DMSO (20%) and 0.9% saline (80%) and was titrated with microliter quantities of 1.0 N HCl until the solid drug was in solution at a pH of 4.0–4.5. The DMSO/saline solution was administered as the vehicle control. The dose of 1.0 mg/kg TBZ was based on extensive piloting in our laboratory. The doses of *(**S**)*-CE-123 were selected based on extensive pilot studies and information about its relative affinity for DAT.

#### Behavioral Pharmacology Experiments

Trained rats (n = 8) were administered either TBZ (1.0 mg/kg) or vehicle, and *(**S**)*-CE-123 (6.0, 12.0, and 24.0 mg/kg) or vehicle, *via* intraperitoneal (IP) injections on drug testing days. Rats received TBZ or vehicle 120 min before testing and *(**S**)*-CE-123 or vehicle 30 min before testing. The experiment used a within-groups design, with each rat receiving each drug treatment in a randomly varied order (one treatment per week, with none of the treatment sequences repeated across different animals). Sample size was determined based on information obtained from pilot studies done in our laboratory. Experimenter blinding was not necessary due to the objective nature of the data collection. The following five treatment combinations were given: TBZ vehicle + *(**S**)*-CE-123 vehicle; 1.0 mg/kg TBZ + *(**S**)*-CE-123 vehicle; 1/0 mg/kg TBZ + 6.0 mg/kg *(**S**)*-CE-123; 1.0 mg/kg TBZ + 12.0 mg/kg *(**S**)*-CE-123; 1.0 mg/kg TBZ + 24.0 mg/kg *(**S**)*-CE-123. The endpoint of the experiment was marked by the completion of the last scheduled drug treatments for each rat, after having received each drug treatment in a randomly varied order.

Following the initial behavioral experiment, *(**S**)*-CE-123 was tested on a different group of rats (n = 7) to assess whether it had a behavioral effect when administered alone. Rats were trained on the FR5/chow feeding choice task as described previously. Thirty minutes prior to the testing session, rats were administered either vehicle or 24.0 mg/kg *(**S**)*-CE-123. One week later, rats received either vehicle or 24.0 mg/kg *(**S**)*-CE-123 so that the treatment orders were counterbalanced. Only the highest dose of *(**S**)*-CE-123 (24.0 mg/kg) was chosen for testing in this paradigm.

A third behavioral experiment was conducted to determine if *(**S**)*-CE-123 had an effect on rats’ behavior on the PROG/chow feeding choice task when administered alone. Thirty minutes prior to the testing session, trained rats (n = 15) were administered either vehicle or 6.0, 12.0, or 24.0 mg/kg *(**S**)*-CE-123. This experiment used a within-groups design, with each rat receiving each drug treatment in a randomly varied order. Treatments were administered once per week over the course of 4 weeks.

### *In Vivo* Microdialysis

#### Surgery

Male Sprague-Dawley rats (275–300 g; Harlan, Italy) were anesthetized with fentanyl (0.06 mg/kg IP), placed in a stereotaxic apparatus, and implanted with vertical dialysis probes prepared as previously described (De Luca et al., 2016) with 1.5 mm dialyzing portion. According to the rat brain atlas of Paxinos and Watson (1998), unilateral probes were implanted in the nucleus accumbens shell (A +2.2, L +1.0 from bregma, V −7.8 from dura; n = 10) or nucleus accumbens core (A +1.4; L +1.6 from bregma, V −7.6 from dura; n = 7).

#### Analytical Procedure

On the day following surgery, animals were connected to an infusion pump, and probes were perfused with Ringer’s solution (147 mM NaCl, 4 mM KCl, 2.2 mM CaCl_2_) at a constant rate of 1 µl/min. After rinsing for at least 1 h, dialysate samples (20 µl) were collected every 20 min and injected into an HPLC equipped with a reverse phase column (C8 3.5 µm, Waters, USA) and a coulometric detector (ESA, Coulochem II) to quantify DA. The electrodes of the analytical cell were set at +125 mV (oxidation) and −175 mV (reduction) to detect dopamine. The mobile phase composition was: 50 mM NaH_2_PO_4_, 0.1 mM Na_2_EDTA, 0.5 mM n-octylsulfate, and 15% (v/v) methanol. The sensitivity of the assay for DA was 5 fmol/sample. When the DA did not differ more than 10% in three consecutive samples, the average value was considered as the basal level of DA. The animals were treated with vehicle or ***(S)***-CE-123 24.0 mg/kg IP, and monoamine levels were monitored for 3 h from the start of the treatment.

#### Histology

At the end of the experiment, animals were sacrificed, and brains were removed and stored in formalin (8%). Brains were sliced and stained with Nissl stain for histological examination in order to verify the correct placement of the microdialysis probes.

### Statistical Analysis

#### Behavioral Pharmacology Experiments

Total number of lever presses and gram quantity of chow intake from the 30-min sessions were analyzed using repeated measures ANOVA. A statistical program (SPSS, version 25) was used to perform all analyses. Since there were significant overall F values for the three behavioral measures being used, nonorthogonal planned comparisons were performed, using the overall error term to assess differences between each treatment and the control condition. The number of comparisons was restricted to the number of treatments minus one (Keppel, 1991). Statistical outliers were predefined as any point that is more than two standard deviations from the mean. No data from this study were excluded as outliers. Microdialysis data were expressed as percent of the last three baseline samples and were analyzed by a 4 group × 12 sample factorial ANOVA with repeated measures on the sample factor. Since there was a significant group × sample interaction, so subsequent analysis of simple main effects examined the effect of the sample factor for each of the four groups. Results from treatments showing significant overall changes were subjected to Tukey’s tests for *post hoc* comparisons, with significance at p < 0.05.

## Results

### FR5/Chow Feeding Choice Task

**Figure 2:**
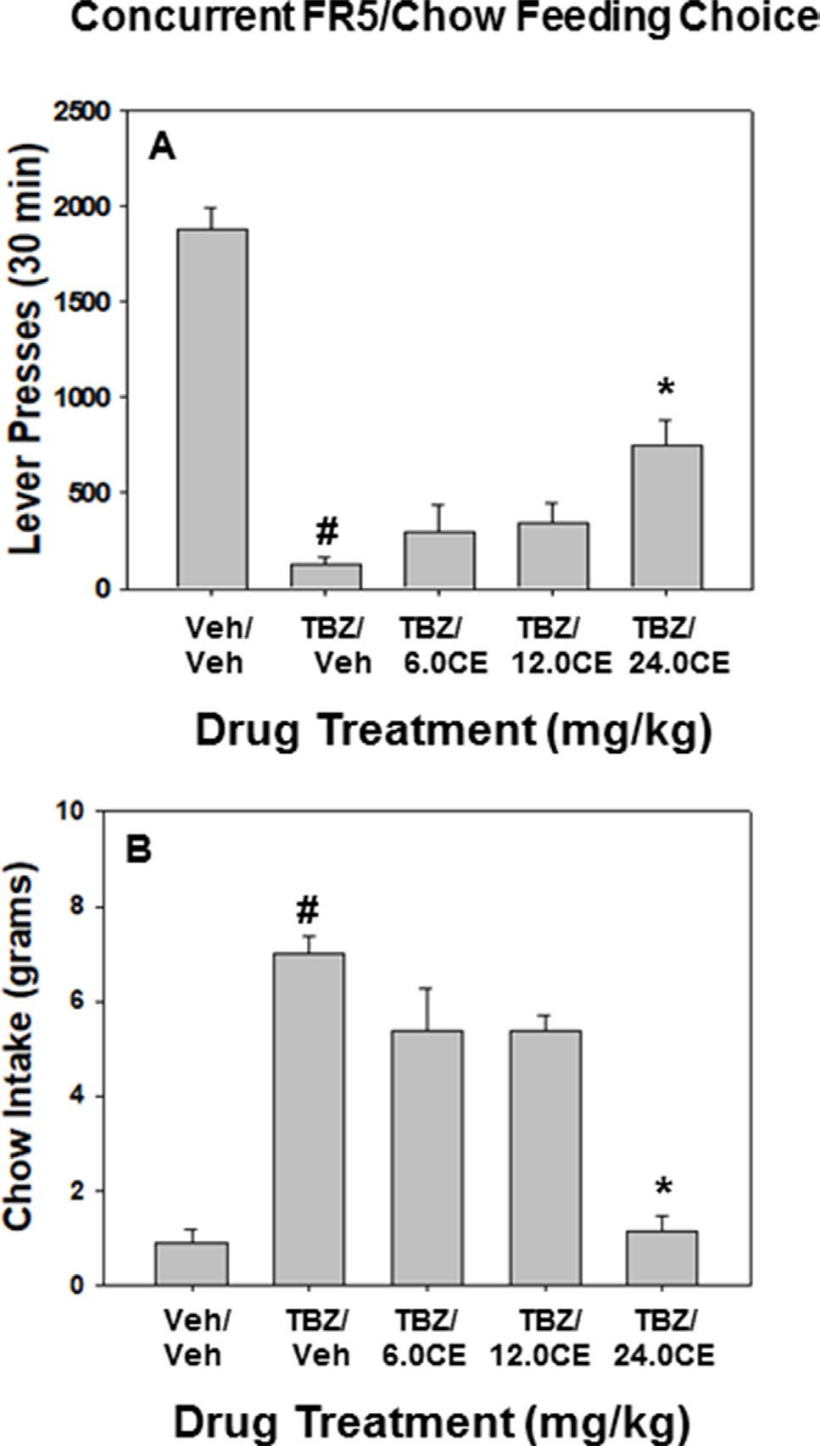
The effects of the DAT blocker (*S*)-CE-123 on TBZ-induced changes in performance on the concurrent lever pressing/chow-feeding choice procedure. Rats (n = 8) received intraperitoneal injections of vehicle plus vehicle (v/v), 1.0 mg/kg TBZ plus vehicle (TBZ/V), or TBZ plus 6.0, 12.0, or 24.0 mg/kg doses of (*S*)-CE-123. **(A)** Mean (± SEM) number of lever presses (FR5/chow schedule) during the 30-min session. ^#^ *p* < 0.001, TBZ plus vehicle significantly differed from vehicle plus vehicle; *p < 0.01, TBZ plus 24.0 mg/kg (*S*)-CE-123 significantly differed from TBZ plus vehicle. **(B)** Mean (± SEM) gram quantity of chow intake. ^#^ *p* < 0.001, TBZ plus vehicle significantly differed from vehicle plus vehicle; **p < 0.001, TBZ plus 24.0 mg/kg (*S*)-CE-123 significantly differed from TBZ plus vehicle.

**Figure 2** shows the results of the FR5/chow feeding choice experiment. TBZ shifted effort-based choice, decreasing lever pressing and increasing chow intake. Repeated measures ANOVA revealed that there was an overall significant effect of drug treatment on lever pressing [F(4,28) = 34.625, p < 0.001] (**Figure 2A**). Planned comparisons showed that TBZ significantly decreased lever pressing compared with vehicle treatment {TBZ/Veh vs. Veh/Veh [F(1,28) = 105.87, p < 0.001]}. There was also significant overall effects of drug treatment on chow intake [F(4,28) = 27.280, p < 0.001], and TBZ alone significantly increased chow intake relative to vehicle treatment {TBZ/Veh vs. Veh/Veh [F(1,28) = 66.625, p < 0.001]} (**Figure 2B**). Additional planned comparisons revealed that coadministration of the dose of 24.0 mg/kg *(**S**)*-CE-123 with TBZ significantly attenuated the effects of TBZ on lever pressing {TBZ plus 24.0 mg/kg vs. TBZ/Veh [F(1,28) = 13.2866, p < 0.01]} and chow intake {TBZ plus 24.0 mg/kg vs. TBZ/Veh [F(1,28) = 61.014, p < 0.001]}.

**Figure 3:**
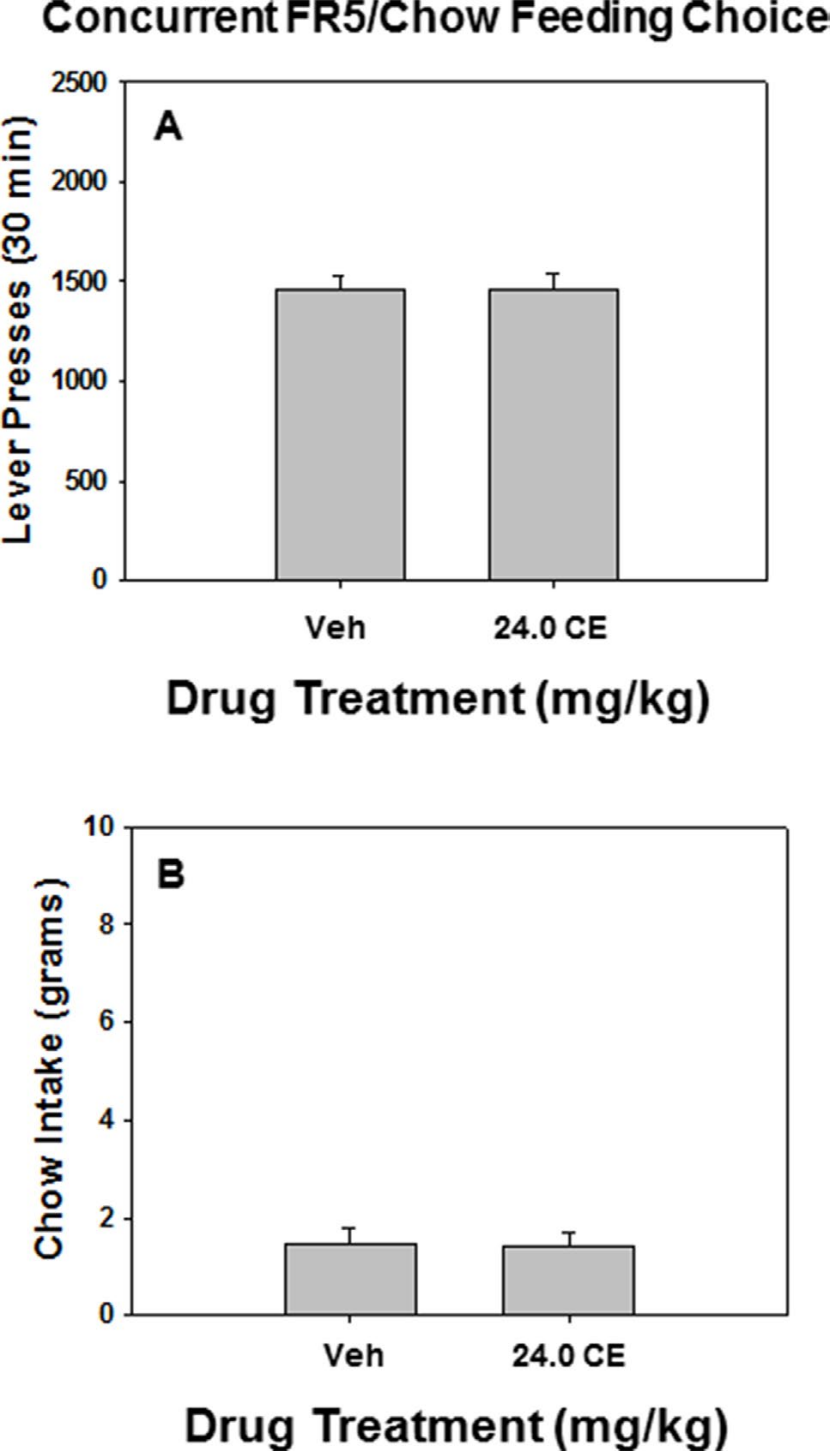
The effects of the DAT blocker (*S*)-CE-123 on performance on the concurrent lever pressing/chow-feeding choice procedure. Rats (n = 7) received intraperitoneal injections of vehicle (Veh), or 24.0 mg/kg (*S*)-CE-123. **(A)** Mean (± SEM) number of lever presses (FR5/chow schedule) during the 30-min session. Lever presses of rats treated with 24 mg/kg (*S*)-CE-123 did not significantly differ from rats treated with vehicle (p = n.s.). **(B)** Mean (± SEM) gram quantity of chow intake. Chow intake of treated with 24 mg/kg (*S*)-CE-123 did not significantly differ from rats treated with vehicle (p = n.s.).

As shown in **Figure 3**, there was no effect of drug treatment relative to vehicle on lever pressing or chow intake on the FR5/chow feeding choice task when 24.0 mg/kg *(**S**)*-CE-123 was administered alone prior to testing. Repeated measures ANOVA indicated no significant difference between rats treated with 24.0 mg/kg *(**S**)*-CE-123 and vehicle-treated rats on lever pressing [F(1,6) = 0.012, p = n.s]. (**Figure 3A**) or chow intake [F(1,6) = 0.030, p = n.s]. (**Figure 3B**).

### PROG/Chow Feeding Choice Task

**Figure 4:**
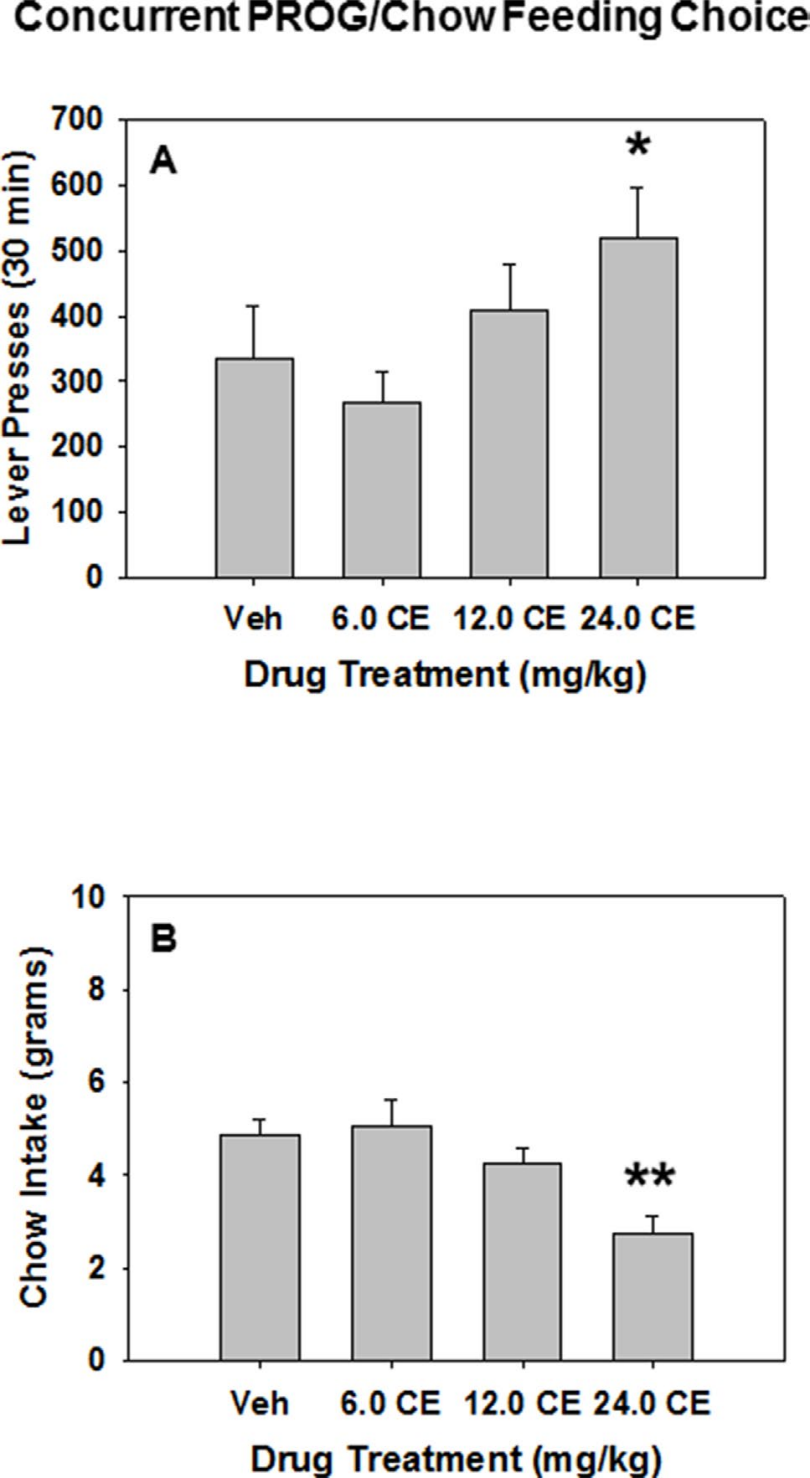
The effects of the DAT blocker (*S*)-CE-123 on performance on the concurrent lever pressing/chow-feeding choice procedure. Rats (n = 15) received intraperitoneal injections of vehicle (Veh), 6.0, 12.0, or 24.0 mg/kg doses of (*S*)-CE-123. **(A)** Mean (± SEM) number of lever presses (PROG/chow schedule) during the 30-min session. *p < 0.05, 24.0 mg/kg (*S*)-CE-123 significantly differed from vehicle. **(B)** Mean (± SEM) gram quantity of chow intake. **p < 0.001, 24.0 mg/kg (*S*)-CE-123 significantly differed from vehicle.

*(**S**)*-CE-123 was tested for its behavioral effect on the PROG/chow feeding choice task when administered alone (i.e., in the absence of TBZ). Repeated measures ANOVA revealed an overall main effect of drug treatment on lever pressing [F(3,42) = 3.165, p < 0.05], and planned comparisons demonstrated that lever presses were significantly increased at 24.0 mg/kg *(**S**)*-CE-123 compared with vehicle treatment (Veh vs. 24.0 mg/kg *(**S**)*-CE-123 [F(1,42) = 4.061, p < 0.05] (**Figure 4A**). Drug treatment with *(**S**)*-CE-123 also had a significant effect on chow intake during the PROG/chow session, indicated by a repeated measures ANOVA [F(3,42) = 13.771, p < 0.001], with a significant reduction in chow intake at the 24.0 mg/kg dose compared with vehicle (Veh vs. 24.0 mg/kg *(**S**)*-CE-123 [F(1,42) = 28.107, p < 0.001] (**Figure 4B**).

### Microdialysis

**Figure 5:**
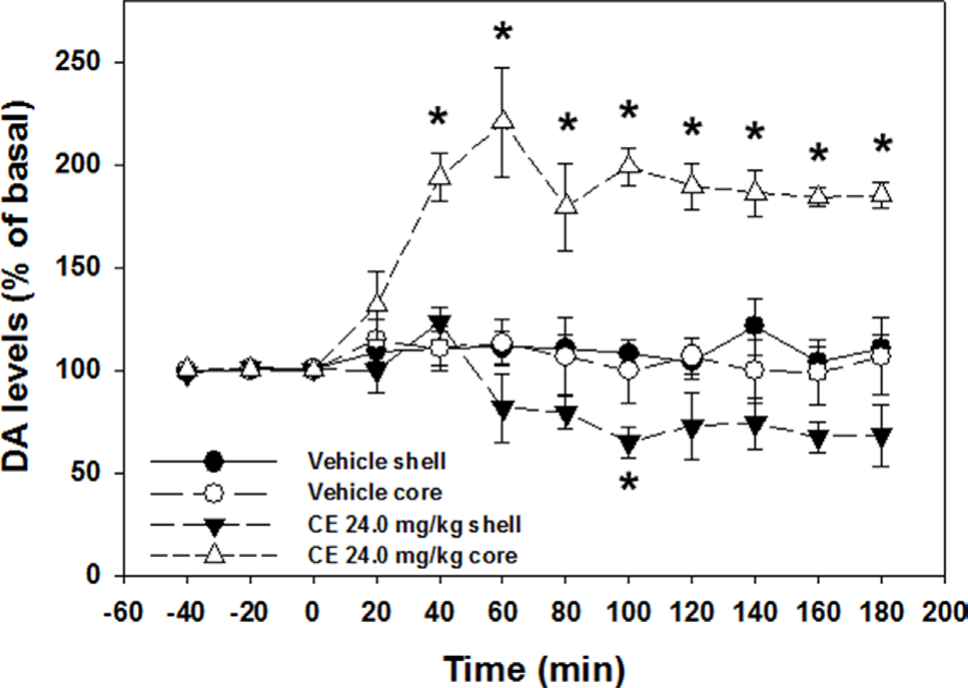
Effect of vehicle or 24.0 mg/kg ***(S)***-CE-123 on extracellular DA in the nucleus accumbens core and shell measured by microdialysis. Mean (± SEM) extracellular DA (expressed as percent baseline) in 20-min samples across 180 min. Three baseline samples were collected prior to injection, followed by nine postinjection samples. *Significantly different from vehicle, p < 0.05.

**Figure 6:**
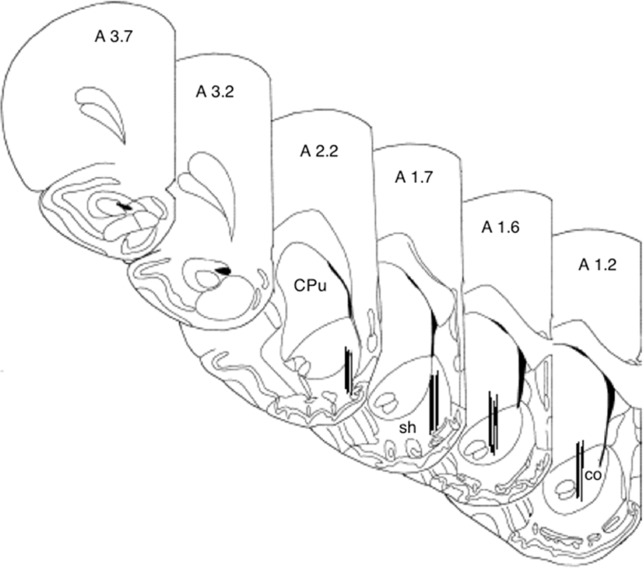
Histology figure showing microdialysis probe placements for nucleus accumbens core (co) and shell (sh). Drawings are from Paxinos and Watson (1998). CPu, caudate putamen.

One-way ANOVA revealed no differences in basal DA levels between nucleus accumbens shell vehicle (mean 130.46 fmoles DA/20 µl; SEM 19.01) and nucleus accumbens shell 24.0 mg/kg *(**S**)*-CE-123 (mean 90.65 fmoles DA/20 µl; SEM 12.17) conditions [F(1,9) = 2.411, p = n.s.], or nucleus accumbens core vehicle (mean 91.28 fmoles DA/20 µl; SEM 27.37) and nucleus accumbens core 24.0 mg/kg *(**S**)*-CE-123 (mean 90.25 fmoles DA/20 µl; SEM 17.12) conditions [F(1,6) = 0.001, p = n.s.]. A 4 group × 12 sample repeated measures factorial ANOVA revealed a significant overall effect of treatment group [F(3,13) = 29.680, p < 0.001], a significant effect of sample [F(11,143) = 6.756, p < 0.001], and a significant treatment × sample interaction [F(33,143) = 8.019, p < 0.001] (**Figure 5**). Analysis of simple effects in which each condition was analyzed separately showed that there was a significant increase in extracellular DA in the nucleus accumbens core in the group that received 24.0 mg/kg *(**S**)*-CE-123 [F(11,33) = 11.950, p < 0.001]. Tukey’s tests indicated that 24.0 mg/kg ***(S)***-CE-123 administration increased nucleus accumbens core DA significantly from baseline at samples taken 40–180 min after injection (p < 0.05). Analysis of simple effects also showed that there was a significant difference in extracellular DA in the nucleus accumbens shell in the group treated with 24.0 mg/kg ***(S)***-CE-123 [F(11,33) = 7.049, p < 0.001] from baseline, and a Tukey’s test indicated a significant reduction at 100 min after injection (p < 0.05). Injections of vehicle had no significant effect on extracellular DA levels in either nucleus accumbens core or shell (**Figure 5**). **Figure 6** depicts the placements of dialysis probes in the nucleus accumbens core and shell.

## Discussion

The synthesis of *(**S**)*-CE-123 afforded highly pure desired enantiomer, and NMRs and mass spectroscopy methods yielded spectra unambiguously identifying the compound (see **Supplementary Materials**). In previous studies, the absolute configuration was characterized as the *(**S**)*-enantiomer that was shown to be stronger at inhibiting DAT than the *(**R**)*-enantiomer (Nikiforuk et al., 2017; Kristofova et al., 2018). In uptake inhibition assays conducted in HEK293 cells stably expressing human isoforms of DAT, NET, and SERT, the EC_50_ for inhibition of DAT by *(**S**)*-CE-123 was reported to be 2.76 × 10^-6^ M, which was 30-fold selective relative to inhibition of NET and more than 400-fold selective compared with SERT inhibition (Nikiforuk et al., 2017, **Supplementary Figure 17A**). Moreover, (***S***)-CE-123 is able to penetrate the blood–brain barrier after systemic injection (Kristofova et al., 2018). In the present paper, we report the first enantioselective synthesis of *(**S**)*-CE-123, as well as behavioral studies focusing on effort-related choice behavior.

The behavioral studies sought to provide an initial characterization of the behavioral effects of the novel atypical DAT inhibitor, *(**S**)*-CE-123, in terms of its ability to alter effort-related choice behavior in rats. Specifically, one of the experiments employed a task designed to measure effort-related choice, which was used to assess the ability of *(**S**)*-CE-123 to reverse the effort-related effects of TBZ. In this experiment, rats trained on the FR5/chow feeding choice task shifted from the high-effort option (FR5 lever pressing) to the low-effort option (chow intake) when treated with TBZ, consistent with previous findings (Nunes et al., 2013; Randall et al., 2014; Yohn et al., 2015a; Yohn et al., 2016b; Yohn et al., 2016c). Several previous studies with TBZ have shown that these shifts in effort-based choice are not due to changes in food intake, food preference, sucrose preference, motor incapacity, or reference memory and do not resemble the effects of appetite suppressant drugs or reward devaluation by pre-feeding (Nunes et al., 2013; Randall et al., 2014; Pardo et al., 2015; Yohn et al., 2015a). Coadministration of 24.0 mg/kg *(**S**)*-CE-123 with TBZ produced a significant but partial reversal of the effects of TBZ, as indicated by an increased selection of FR5 lever pressing (**Figure 2A**) and a decrease in chow intake (**Figure 2B**) compared with TBZ plus vehicle. In a parallel control experiment in rats tested on the same task, 24.0 mg/kg *(**S**)*-CE-123 administered in the absence of TBZ did not have any effect on lever pressing (**Figure 3A**) or chow intake (**Figure 3B**).

In order to develop a more comprehensive characterization of the effort-related actions of monoamine uptake inhibitors, it also is important to administer them alone, in the absence of a drug like TBZ, because that would allow for the assessment of both increases and decreases in performance, in the absence of a drug condition like TBZ, which induces an impairment. In previous studies, the PROG/chow feeding choice task has been used to assess the ability of drugs to enhance selection of high-effort PROG lever pressing (e.g., Randall et al., 2012; Randall et al., 2015). This task is especially well suited for such an assessment because unlike the FR5/chow feeding choice task, baseline lever pressing rates are relatively low on the PROG component due to the increasing response requirement of the PROG schedule. Thus, as the ratio work requirement gets gradually higher, animals eventually shift over to chow consumption (Randall et al., 2012; Randall et al., 2014; Randall et al., 2015). Previous research has demonstrated that the NET inhibitors desipramine and atomoxetine, as well as the SERT inhibitor fluoxetine, all fail to increase PROG lever pressing in rats tested on the PROG/chow feeding choice procedure (Yohn et al., 2016d). In contrast, drugs that inhibit DAT, including bupropion, lisdexamfetamine, PRX-14040, MRZ-9547, and GBR 12909, all have been shown to increase selection of PROG responding on this task (Sommer et al., 2014; Randall et al., 2015; Yohn et al., 2016b; Yohn et al., 2016c; Yohn et al., 2016d). In the present studies, (**S**)-CE-123 was shown to significantly increase PROG lever pressing and decrease chow intake in rats tested on the PROG/choice procedure (**Figure 4**). Furthermore, the effective dose of (**S**)-CE-123 in both the TBZ reversal and PROG/chow feeding choice studies, 24.0 mg/kg, significantly increased extracellular DA in nucleus accumbens core as measured by microdialysis (**Figure 5**). In previous studies in which PROG lever pressing and bupropion-induced changes in extracellular DA were investigated in separate groups of animals (Randall et al., 2015), the magnitude of the increases in PROG lever pressing and accumbens core DA shown after administration of 20.0 mg/kg bupropion were comparable with those shown in the present studies with 24.0 mg/kg (***S***)-CE-123.

It is not clear why extracellular DA did not increase in the nucleus accumbens shell after injections of (***S***)-CE-123. In a recent study, Camats-Perna et al. (2019) reported that (***S***)-CE-123 at doses of 10.0 and 100.0 mg/kg increased extracellular DA in the nucleus accumbens of mice. However, given the small size of the mouse brain, and the active surface area of the probe, it is evident that both core and shell DA contributed to this finding. Previous studies have looked at the neurochemical effects of modafinil, the parent compound for (***S***)-CE-123. Bobak et al. (2016) used voltammetry methods to study the effects of modafinil on extracellular DA in rats; they employed a ventral striatal electrode placement that did not specifically target the shell and observed that doses of modafinil up to 100.0 and 300.0 mg/kg increased DA transmission. Mereu et al. (2017) conducted a microdialysis study of core and shell DA in mice and observed that while 17.0 mg/kg modafnil did not significantly increase shell DA, 30.0 mg/kg produced a modest increase in DA in core and shell, while higher doses (100.0 and 300.0 mg/kg) produced very robust increases. These high doses of modafinil (100.0 and 300.0 mg/kg) are 10 times the behaviorally effective doses of modafinil that we have used in our rat studies with effort-based choice (e.g. 7.5, 15.0, and 30.0 mg/kg; Salamone et al., 2016a).

Despite the negative findings with shell DA, core DA did substantially increase in the present study, and previous work has shown that the core subregion is a critical site at which effort-based choice is regulated. Sokoloski and Salamone (1998) reported that the shift from lever pressing to chow intake in rats tested on the concurrent FR5/chow feeding choice task was produced after injections of the neurotoxic agent 6-hydroxydopamine into nucleus accumbens core but not the shell. Ghods-Sharifi and Floresco (2010) showed that a low-effort bias in rats tested on an effort-discounting task was produced after inactivation of the nucleus accumbens core but not the shell. Randall et al. (2012) observed that a postsynaptic marker of DA transmission (expression of DA and cyclic AMP dependent phosphoprotein phosphorylated at the threonine-34 site) was significantly higher in animals with high PROG lever pressing compared with low PROG lever pressing; this was seen in accumbens core but not shell. Furthermore, local accumbens core injections of the DA antagonist flupenthixol (Farrar et al., 2010) and TBZ (Nunes et al., 2013) shifted choice behavior from FR5 lever pressing to chow intake, while injections into overlying neostriatum did not.

Several lines of evidence indicate that DAT blockers can have pro-motivational effects in animal models (Nunes et al., 2013; Randall et al., 2015; Sommer et al., 2014; Yohn et al., 2016a; Yohn et al., 2016b; Yohn et al., 2016c; Yohn et al., 2016d). Although DAT inhibition is a commonly used descriptor for a broad class of drugs, there is considerable heterogeneity within this group of compounds. For example, amphetamines such as d-amphetamine and methamphetamine are not only competitive inhibitors of DAT but also substrates that are transported into the neuronal terminal and stimulate release (Ferris et al., 2011). Cocaine is a classical DAT inhibitor with a rapid onset and offset of action (Tanda et al., 2013). Unfortunately, most classical DAT inhibitors and releasing agents have a well-characterized abuse liability (Todtenkopf and Carlezon, 2006; Ostlund et al., 2014; Dong et al., 2017), which limits their therapeutic utility for treating motivational dysfunction in psychiatry. However, not all drugs that bind to the DAT share cocaine’s behavioral profile or have a high potential for abuse. Over the last several years, atypical DAT inhibitors have been under development, which have characteristics that differ from cocaine. GBR12909 was developed as a potential treatment for cocaine addiction (Rothman et al., 2008). GBR12909 blocks many of the effects of cocaine and on its own has produced mixed effects in terms of cocaine-like actions; it did not produce psychostimulant effects in people in the dose ranges tested (Sogaard et al., 1990; Preti, 2000), and although it supported self-administration in primates, its efficacy was lower than that of cocaine (Woolverton et al., 2001). Although GBR12909 was discontinued as a potential treatment for cocaine abuse due to its cardiac effects, analogs of GBR12909 have been studied for their atypical binding characteristics (Rothman et al., 2008). The DAT has multiple functional conformations, and several benztropine analogs have been shown to bind to the DAT in a manner that is distinct from that of cocaine (Schmitt et al., 2008; Kohut et al., 2014). Some of these benztropine analogs have been shown to increase extracellular DA in nucleus accumbens, albiet over a much longer time course than cocaine (Tanda et al., 2013). Unlike cocaine, these drugs failed to induce conditioned place preference (Tanda et al., 2013).

*(**S**)*-CE-123 is an analog of modafinil, which also binds to the DAT with an atypical profile (Schmitt and Reith, 2011; Cao et al., 2016). Modafinil inhibits DAT and increases extracellular DA over a broad time course (Mereu et al., 2017), and while this drug has been shown to have cognitive enhancing and pro-motivational effects, it has a relatively low abuse liability (Mereu et al., 2013; Müller et al., 2013). Modafinil has been reported to improve fatigue symptoms in depressed patients (Lam et al., 2007), and recent studies from our laboratory have shown that modafinil can reverse the low-effort bias induced by TBZ in rats (Salamone et al., 2016a; Yohn et al., 2016c). When bound to the DAT, *(**S**)*-CE-123 also has an atypical pattern compared with cocaine, in that it interacts with the negatively charged ASP79 locus (Kristofova et al., 2018). Moreover, *(**S**)*-CE-123 acts as a highly selective atypical inhibitor of DAT relative to NET and SERT and is more selective for DAT than modafinil (Kristofova et al., 2018). *(**S**)*-CE-123 has been shown to enhance cognitive flexibility, improve memory acquisition and retrieval, and reduce impulsivity in rats (Nikiforuk et al., 2017; Kristofova et al., 2018), and in the present studies, it reverses the motivational impairments induced by TBZ and increases selection of high-effort PROG lever pressing.

In the present studies, a partial reversal of the effort-related motivational dysfunction induced by TBZ was achieved with *(**S**)*-CE-123 administration in a dose range of 6.0–24.0 mg/kg. The efficacy of *(**S**)*-CE-123 at restoring lever pressing appears to be lower than that of bupropion, GBR12909, lisdexamfetamine, methylphenidate, and modafinil in rats tested on similar procedures (Nunes et al., 2013; Salamone et al., 2016a; Yohn et al., 2016a; Yohn et al., 2016b; Yohn et al., 2016c), although most drugs that block DAT tend to produce only partial reversals of the effects of TBZ (i.e., approximately 60–85% restoration of responding). Nevertheless, *(**S**)*-CE-123 was highly efficacious at reversing the TBZ-induced increase in chow intake (**Figure 2B**), which may indicate that this drug also has appetite-suppressant actions. These potential appetite-suppressant effects of (**S**)-CE-123 would suggest that the increases in lever pressing seen in the FR5 and PROG/chow feeding choice studies are not due to an increase in appetite. Drugs that inhibit DAT bind across a broad range of affinities, which is potentially related to differences in potency seen in behavioral experiments. High-affinity drugs such as d-amphetamine, methylphenidate, PRX-14040, and GBR12909 have a relatively high potency for reversing the effects of TBZ (Salamone et al., 2016a; Yohn et al., 2016a; Yohn et al., 2016b; Yohn et al., 2016c). In contrast, compounds with lower DAT binding affinities such as bupropion, modafinil, and *(**S**)*-CE-123 tend to be less potent at reversing the effects of TBZ (i.e., they require higher doses; Nunes et al., 2013; Salamone et al., 2017; present studies).

Future studies should examine a larger group of atypical DAT blockers to determine the overall relation between the neurochemical characteristics of these compounds (e.g. DAT affinity, selectivity and binding locus, effects on DAT trafficking (Mash et al., 2002; Vaughan and Foster, 2013), dynamics of effects on extracellular DA) and their effort-related behavioral effects. Furthermore, behavioral studies should be extended to include tasks that provide information about other behavioral effects, including locomotor activity, and most importantly, potential abuse liability. Drugs such as amphetamine and methylphenidate can increase selection of high-effort activity in humans (Wardle et al., 2011) and can improve motivational function in depressed patients (Stotz et al., 1999), but they also are well known for their abuse liability. In contrast, the atypical DAT blocker modafinil has been reported to improve motivational function in depressed patients (Lam et al., 2007), albeit without a strong abuse liability. The molecular interactions that occur as a result of the particular functional configuration of the DAT as it binds to drugs that block its action may be related to the diversity of patterns of abuse potential seen across multiple drugs (Tanda et al., 2013). Thus, the binding characteristics of drugs like modafinil and its analogs, such as *(**S**)*-CE-123, suggest that novel compounds may ultimately be identified that are suitable for treating motivational dysfunction, and future studies should determine if *(**S**)*-CE-123 shows signs of having substantial abuse potential.

## Ethics Statement

This study was carried out in accordance with the recommendations of the National Institute of Health guidelines and the Institutional Animal Care and Use Committee of the University of Connecticut. The protocol was approved by the Institutional Animal Care and Use Committee of the University of Connecticut.

## Author Contributions

RR contributed to writing of the manuscript and performed the statistical analyses of the behavioral experiments. RR, RS, RP, SS, and JY carried out the behavioral experiments. PK and VD synthesized the compounds, EU did the NMR assignments, MZ the mass spectrometry analysis, AR did the X-ray analysis, JW did HPLC analyses, and TL provided the laboratory space and advice. MP, ML, and FC performed the in vivo microdialysis aspects of the experiment including the surgical procedure and analysis. GL initiated and planned the study and contributed to writing of the manuscript. JS and MC supervised the design of the behavioral experiments and data analysis and wrote parts of the manuscript.

## Funding

This work was supported by grants to JS from the University of Connecticut Research Foundation, to RS from the Connecticut Institute for Brain and Cognitive Sciences and the Summer Undergraduate Research Fund at the University of Connecticut, and to SS from the University of Connecticut Psychological Sciences Department. MADL was supported by Fondazione di Sardegna (Esercizio finanziario 2017), FIR 2019, Fondo di Sviluppo e Coesione 2014-2020(Project RASSR03071; CUP F76C18000830002).

## Conflict of Interest Statement

The authors declare that the research was conducted in the absence of any commercial or financial relationships that could be construed as a potential conflict of interest.
