# Supplementary material for: The Novel Atypical Dopamine Uptake Inhibitor (S)-CE-123 Partially Reverses the Effort-Related Effects of the Dopamine Depleting Agent Tetrabenazine and Increases Progressive Ratio Responding
Source: Front Pharmacol. 2019 Jun 28;10:682. doi: 10.3389/fphar.2019.00682 (PMC6611521; doi:10.3389/fphar.2019.00682)

Supplementary Material

The Novel Atypical Dopamine Uptake Inhibitor *(S)*-CE-123 Partially Reverses the Effort-related Effects of the Dopamine Depleting Agent Tetrabenazine

Renee A. Rotolo[1§], Vladimir Dragacevic[2§], Predrag Kalaba[2], Ernst Urban[2], Martin Zehl[3], Alexander Roller[4], Judith Wackerlig[2], Thierry Langer[2], Marco Pistis[5], Maria Antonietta De Luca[5], Francesca Caria[5], Rebecca Schwartz[1], Rose E. Presby[1], Jen-Hau Yang[1], Shanna Samels[1], Merce Correa[1,6], Gert Lubec*[7], John D. Salamone*[1]

[1]John Salamone, Department of Psychological Sciences, University of Connecticut, Storrs, CT, USA

[2] Department of Pharmaceutical Chemistry, Faculty of Life Sciences, University of Vienna, Althanstraße 14, 1090 Vienna, Austria

[3]Department of Analytical Chemistry, Faculty of Chemistry, University of Vienna, Währinger Straße 38, 1090 Vienna, Austria

[4]X-ray Structure Analysis Centre, Faculty of Chemistry, University of Vienna, Währinger Straße 38, 1090 Vienna, Austria.

[5]Department of Biomedical Sciences, University of Cagliari, Cagliari, Italy, National Institute of Neuroscience (INN)

[6]Merce Correa, Àrea de Psicobiologia, Campus de Riu Sec, Universitat Jaume I, 12071 Castelló, Spain

[7]Gert Lubec, Department of Neuroproteomics, Paracelsus Medical University, Salzburg, Austria

§ these authors have contributed equally to the work

*Corresponding authors: John D. Salamone (john.salamone@uconn.edu) and Gert Lubec (gert.lubec@lubeclab.com)

Content

Analytical characterization of S-CE-123	Page 2-3
Attribution of absolute configuration	Page 3-4
X-ray data	Page 5-7
HPLC spectra- purity determination	Page 8
HRESIMS spectra	Page 9
HPLC spectra- *ee.* excess determination	Page 10
^{1}H, ^{13}C and 2D NMR spectra	Page 11-39
BioTools VCD Report	Page 40-57

1. Analytical characterization of *S*-CE-123

^{1}H and ^{13}C NMR spectra were recorded on a Bruker Avance 500 NMR spectrometer (UltraShield) using a 5-mm switchable probe (PA BBO 500SB BBF-H-D-05-Z, 1H, BB = ^{19}F and ^{31}P - ^{15}N) with z axis gradients and automatic tuning and matching accessory (Bruker BioSpin). The resonance frequency for ^{1}H NMR was 500.13 MHz and for ^{13}C NMR 125.75 MHz. All measurements were performed for a solution in fully deuterated chloroform or DMSO at 298 K. Standard 1D and gradient-enhanced (ge) 2D experiments, like double quantum filtered (DQF) COSY, NOESY, HSQC, and HMBC, were used as supplied by the manufacturer. Chemical shifts are referenced internally to the residual, non-deuterated solvent signal for acetone ^{1}H (δ 2.09 ppm) and to the carbon signal of the solvent for acetone ^{13}C (δ 30.60 and 205.87 ppm).

HRESIMS spectra were obtained on a maXis HD ESI-Qq-TOF mass spectrometer (Bruker Daltonics, Bremen, Germany). Samples were dissolved to 20 µg/mL in MeOH and directly infused into the ESI source at a flow rate of 3 µL/min with a syringe pump. The ESI ion source was operated as follows: capillary voltage: 0.9 to 4.0 kV (individually optimized), nebulizer: 0.4 bar (N_2), dry gas flow: 4 L/min (N_2), and dry temperature: 200 °C. Mass spectra were recorded in the range of *m/z* 50 – 1550 in the positive-ion mode. The sum formulas were determined using Bruker Compass DataAnalysis 4.2 based on the mass accuracy ($\Delta m/z \leq 2$ ppm) and isotopic pattern matching (SmartFormula algorithm).

The purity of the compound was determined by HPLC on an UltiMate 3000 series system equipped with VWD detector (Dionex/Thermo Fisher Scientific, Germering, Germany). Separation was carried out on an Acclaim 120 C18, 2.1 x 150 mm, 3 µm HPLC column (Thermo Fisher Scientific) using LC-MS-grade water and acetonitrile as mobile phase A and B, respectively. The sample components were separated and eluted with a linear gradient from 10% to 90% B in 25 min followed by an isocratic column cleaning and re-equilibration step. The flow rate was 0.2 mL/min and the column oven temperature was set to 25°C. The purity was determined by HPLC chromatography with UV detector (254 nm), as being the ratio of the peak area of the compound and the total peak areas (i.e., the sum of the areas of all peaks that were not present in the solvent blank). For chiral measurements LC-2010A HT Liquid Chromatograph device

(Shimadzu Corporation, Tokyo, Japan) was used equipped with analytical Chiralpack IA (Daicel Inc., Tokyo, Japan). 100% ACN was used as mobile phase, the flow rate was 1 mL/min and the column oven temperature was set to 25°C. Melting points were measured on Leica Galen III apparatus (Leica Biosystems, Germany).

The final product, *(S)*-CE-123, has been unambiguously characterized. The overall purity of the ***(S)***-enantiomer, determined by a C18 analytical column-based HPLC method using reversed-phase chromatography conditions was 99.3% The experimental molecular mass of the ***(S)***-CE-123 was determined by HRESIMS from the precursor ion *m/z* 314.0667 $[M+H]^+$(calculated for $C_{17}H_{16}NOS_2^+$, 314.0668, Δ = 0.0 ppm). Enantiopurity of the less retained (***S***)-enantiomer was determined to be 94.9% (*ee* 89.8%). Melting point was determined to be 106-107ºC. Chemical shifts assignment by proton and carbon NMR are shown in **Supplementary Table 4**.

2. Attribution of absolute configuration

2.1. Vibrational-circular dichroism (VCD) analysis

Enantiomers of the racemic compound CE-123 were originally separated as described by *Nikiforuk et al. (2017)*. In short, this was achieved by using CHIRALPACK IA semipreparative column (10mm diameter x 20 mm length) (Daicel Inc, Tokyo, Japan) and 100% EtOH as mobile phase.

Determination of absolute configuration of individual enantiomers obtained by chiral separations was performed using the vibrational-circular dichroism (VCD) method. For this purpose, enantiomers were outsourced to European Center for Chirality, University of Antwerp as a part of BioTools Ltd. (Florida, USA).

2.2. X-ray analysis

To confirm the results from the VCD method, the less retained enantiomer of CE-123 was crystallized and X-ray measurement was performed. The X-ray intensity data were measured on

Bruker D8 Venture diffractometer equipped with multilayer monochromator, Mo K/a INCOATEC micro focus sealed tube and Oxford cooling system. The structures were solved by *direct methods* and refined by *full-matrix least-squares techniques*. Non-hydrogen atoms were refined with *anisotropic displacement parameters*. Hydrogen atoms were inserted at calculated positions and refined with riding model. The following software was used: *Bruker SAINT software package*[i] using a narrow-frame algorithm for frame integration, *SADABS*[ii] for absorption correction, *OLEX2*[iii] for structure solution, refinement, molecular diagrams and graphical user-interface, *Shelxle*[iv] for refinement and graphical user-interface *SHELXS-2013*[v] for structure solution, *SHELXL-2013*[vi] for refinement, and *Platon*[vii] for symmetry check. Experimental data, CCDC-Codes, Crystal data, data collection parameters, structure refinement details are given in Supplementary Material section.

Based on the agreement of VCD spectra with Confidence Level of 99%, absolute configurations ***(S)*** and ***(R)*** were assigned to the less and the more retained enantiomer of CE-123, respectively. Experimental details and report are available in the text below. By comparing VCD spectra of the enantiomer of CE-123 synthesized for this study with the VCD spectra of enantiomers of CE-123 to which the absolute configuration had been previously assigned, absolute configuration of the synthesized enantiomer was determined to be (***S***). Based on crystallographic data and following the *Cahn-Ingold-Prelog* rule, the configuration of the active substance was determined to be (***S***), which supports the findings of the VCD method.

[i] Bruker SAINT v8.37A Copyright © 2005-2016 Bruker AXS
[ii] Sheldrick, G. M. (1996). *SADABS.* University of Göttingen, Germany.
[iii] Dolomanov, O.V., Bourhis, L.J., Gildea, R.J, Howard, J.A.K. & Puschmann, H. , OLEX2, (2009), J. Appl. Cryst. 42, 339-341
[iv] C. B. Huebschle, G. M. Sheldrick and B. Dittrich, ShelXle: a Qt graphical user interface for SHELXL, J. Appl. Cryst., 44, (2011) 1281-1284
[v] Sheldrick, G. M. (1996). *SHELXS.* University of Göttingen, Germany.
[vi] Sheldrick, G. M. (1996). *SHELXL.* University of Göttingen, Germany.
[vii] Spek, A. L. (2009). *Acta Cryst.* D65, 148-155.

X-ray Analysis

Supplementary Table 1. Experimental parameters and CCDC-Code.

Sample	Machine	Source	Temp.	Detector Distance	Time/ Frame	#Frames	Frame width	CCDC
	Bruker		[K]	[mm]	[s]		[°]	
PrKaSCE123A2	D8	Mo	100	34	30	2014	0.5	**1837036**

(*S*)-5-((benzhydrylsulfinyl)methyl)thiazole for "Frontiers in Pharmacology"

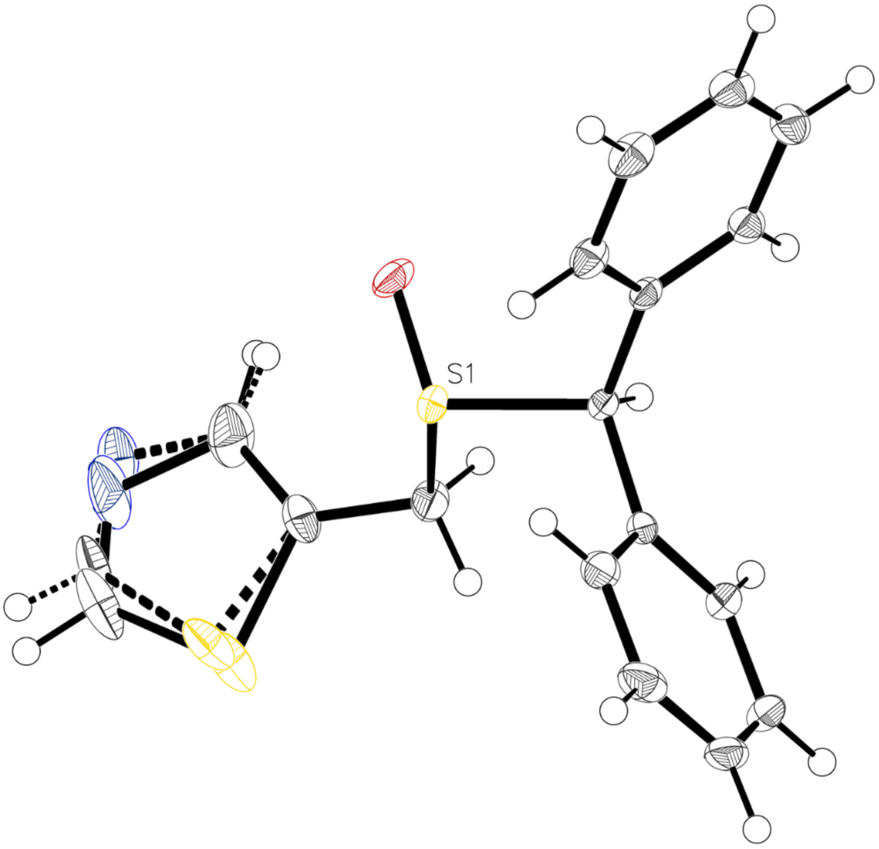

Supplementary Figure 1. Asymmetric Unit of [**PrKaSCE123A2**], drawn with 50% displacement ellipsoid. The chiral space group *P2₁* and the according results of Flack and Hooft parameter (-0.003(14), -0.002(12)) proof the chirality in S1 as "S". The bond precision for C-C single bonds is 0.0025Å. Main Residue disorder is 14%.

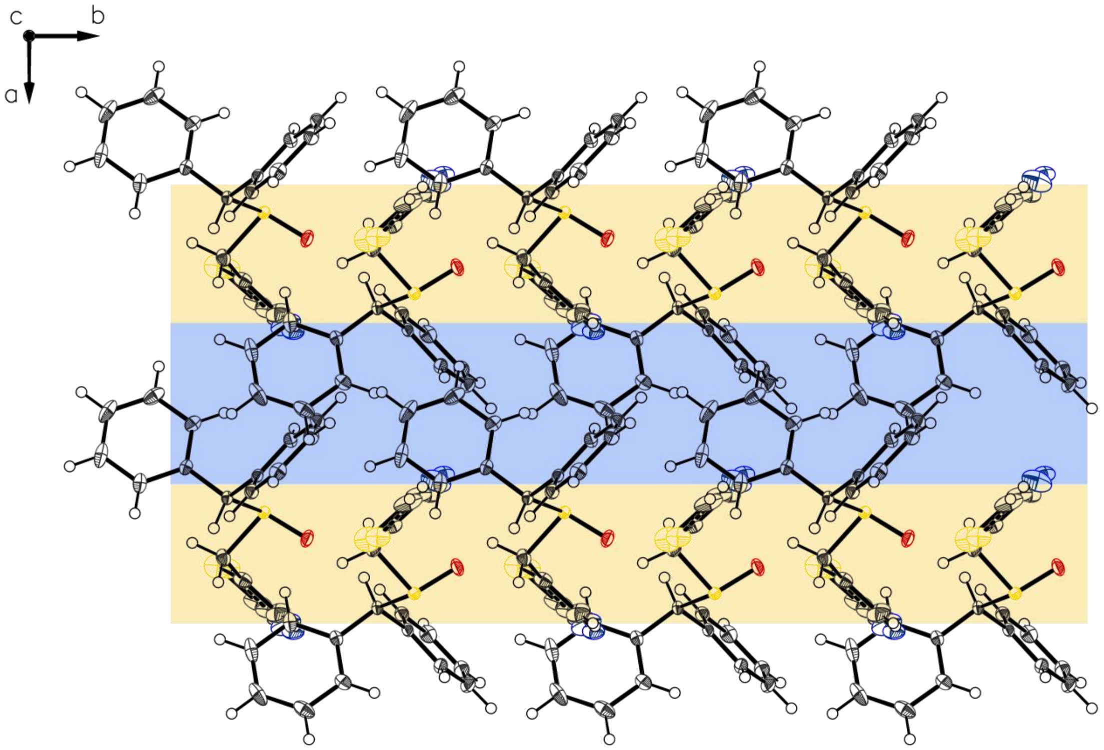

Supplementary Figure 2. Packing of [**PrKaSCE123A2**] in plane a-b. Characterized by alternating regions of polar (orange shaded) and nonpolar (blue shaded) fragments.

Supplementary Table 2. Sample and crystal data of [**PrKaSCE123A2**].

**Chemical formula**	C17H15NOS2	**Crystal system**	monoclinic	
**Formula weight [g/mol]**	313.42	**Space group**	*P21*	
**Temperature [K]**	100	**Z**	2	
**Measurement method**	\f and \w scans	**Volume [Å^3]**	755.27(8)	
**Radiation (Wavelength [Å])**	MoKα (λ = 0.71073)	**Unit cell dimensions [Å] and [°]**	9.4062(6)	90
**Crystal size / [mm^3]**	0.164 × 0.079 × 0.026		8.4553(5)	116.145(2)
**Crystal habit**	clear colourless needle		10.5788(6)	90
**Density (calculated) / [g/cm^3]**	1.378	**Absorption coefficient / [mm^{-1}]**	0.35	
**Abs. correction Tmin**	0.7134	**Abs. correction Tmax**	0.746	
**Abs. correction type**	multiscan	**F(000) [e$^-$]**	328	

Supplementary Table 3. Data collection and structure refinement of [**PrKaSCE123A2**].

**Index ranges**	$-13 \leq h \leq 13$, $-11 \leq k \leq 11$, $-14 \leq l \leq 14$	**Theta range for data collection [°]**	4.824 to 60.176	
**Reflections number**	33075	**Data / restraints / parameters**	4416/4/217	
**Refinement method**	Least squares	**Final R indices**	all data	R1 = 0.0252, wR2 = 0.0593
**Function minimized**	$\Sigma\ w(F_o^2 - F_c^2)^2$		$I>2\sigma(I)$	R1 = 0.0235, wR2 = 0.0585
**Goodness-of-fit on F^2**	1.041	**Weighting scheme**	$w=1/[\sigma^2(F_o^2)+(0.0309P)^2+0.1482P]$	
**Largest diff. peak and hole [e Å^{-3}]**	0.21/-0.18		where $P=(F_o^2+2F_c^2)/3$	

VD-253

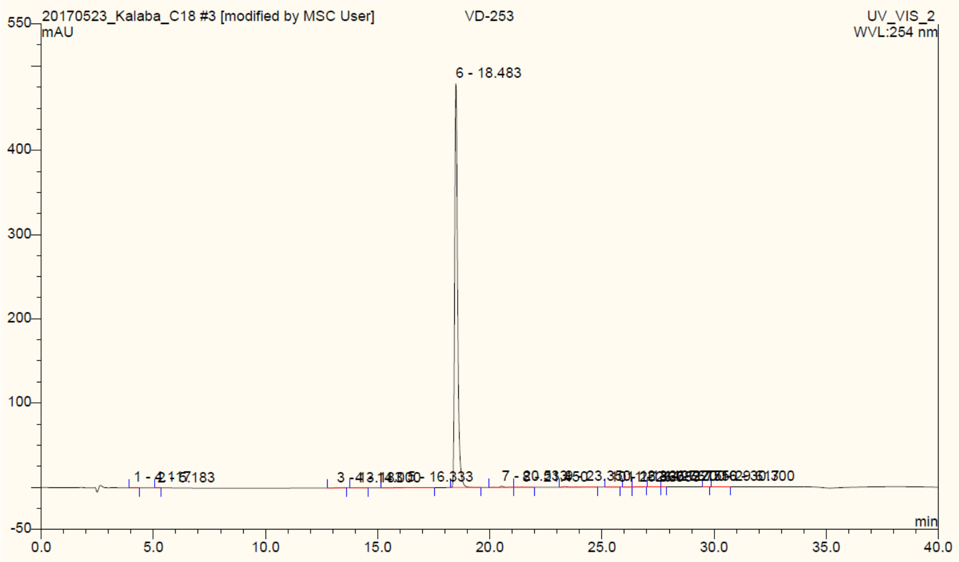

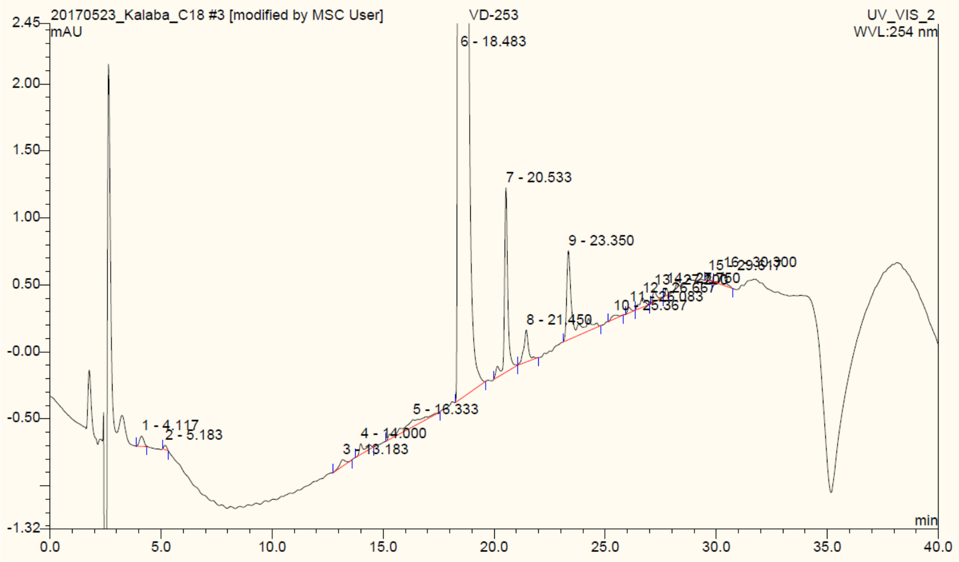

Retention Time:**18.48 min**

Relative Peak Area: **99.05 % (without blank correction)**

99.27 % (with blank correction)

Supplementary Figure 3. HPLC-method determined purity of *S*-CE-123

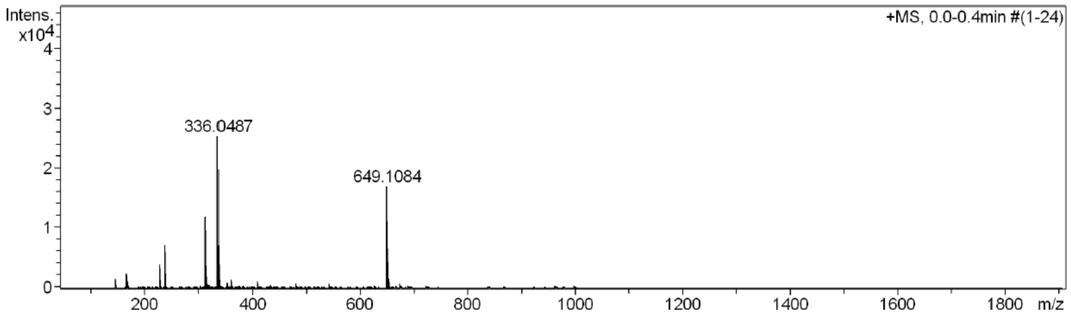

Meas. m/z	#	Formula	Score	m/z	err [mDa]	err [ppm]	mSigma	rdb	e⁻ Conf	N-Rule
314.0668	1	C 17 H 16 N O S 2	100.00	314.0668	0.0	0.0	9.3	10.5	even	ok
	2	C 17 H 8 N 5 O 2	73.94	314.0673	0.5	1.5	14.3	16.5	even	ok
	3	C 16 H 12 N O 6	56.11	314.0659	-0.9	-2.7	18.6	11.5	even	ok
336.0487	1	C 17 H 15 N Na O S 2	100.00	336.0487	0.0	0.1	6.2	10.5	even	ok
	2	C 17 H 7 N 5 Na O 2	34.95	336.0492	0.5	1.5	43.2	16.5	even	ok
	3	C 16 H 11 N Na O 6	29.62	336.0479	-0.8	-2.5	43.3	11.5	even	ok

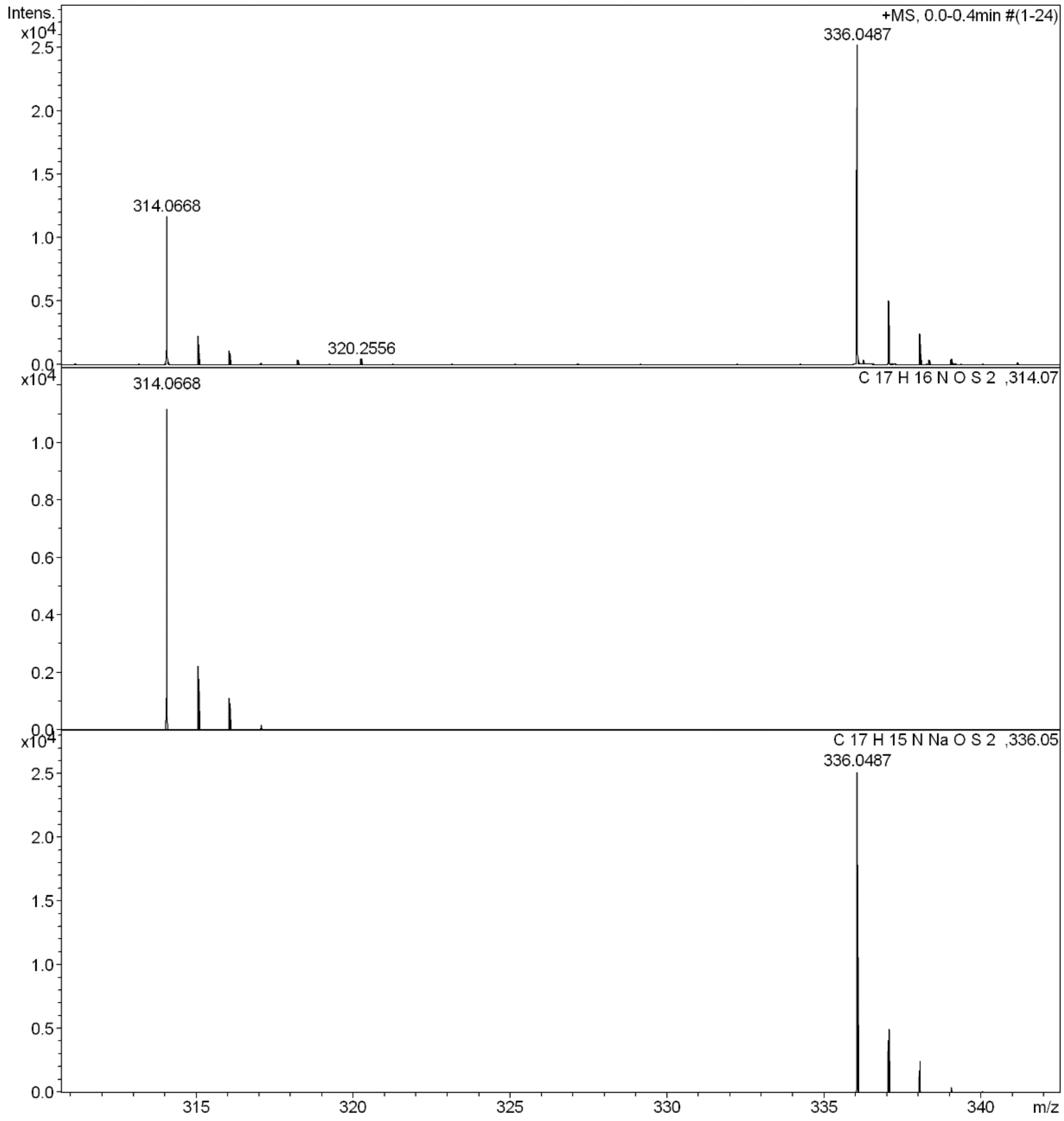

Supplementary Figure 4. High-resolution mass spectrometry spectrum of *S*-CE-123

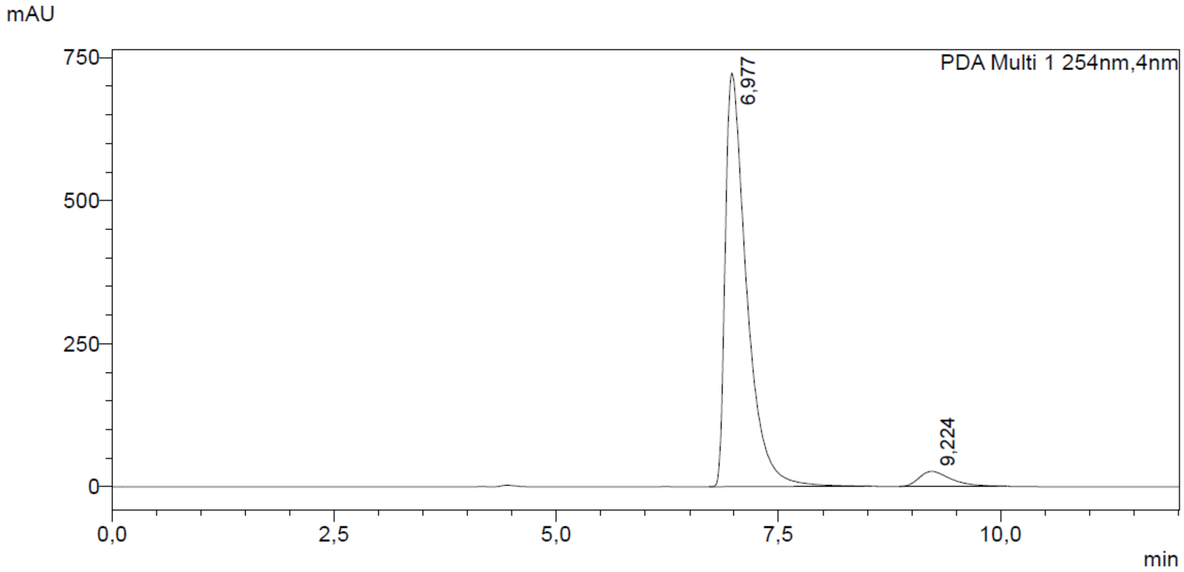

Peak Table

PDA Ch1 254nm

Peak#	Ret. Time	Area	Area%
1	6,977	12030511	94,906
2	9,224	645744	5,094
Total		12676255	100,000

Supplementary Figure 5. HPLC-based method for chiral-resolution determines enantio-purity of S-CE-123 to be 94.9 %.

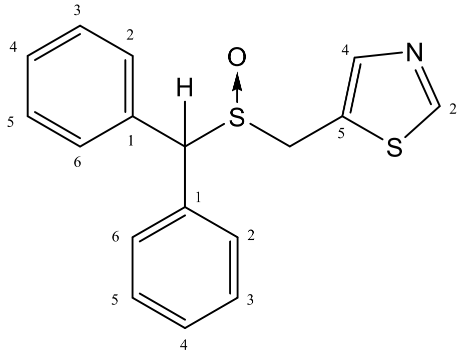

Supplementary Table 4. Chemical shifts and their attribution in ^{1}H and ^{13}C NMR.

**VD253** in d_6Aceton		^{1}H	^{13}C
Ar 2	CH	8,98	156,16
Ar 4	CH	7,72	145,61
Ar 5	C	--	128,46
Ph 1	C	--	138,07
Ph 1	C	--	136,90
Ph 2,6	CH	7,55	131,23
Ph 2,6	CH	7,57	130,31
Ph 3,5	CH	7,47	130,74
Ph 3,5	CH	7,40	130,00
Ph 4	CH	7,40	129,78
Ph 4	CH	7,35	129,58
CH	CH	5,07	71,64
CH_2	CH_2	4,30/3,92	49,03

VD253 in d6Acetone (Proton) 28.9.2017

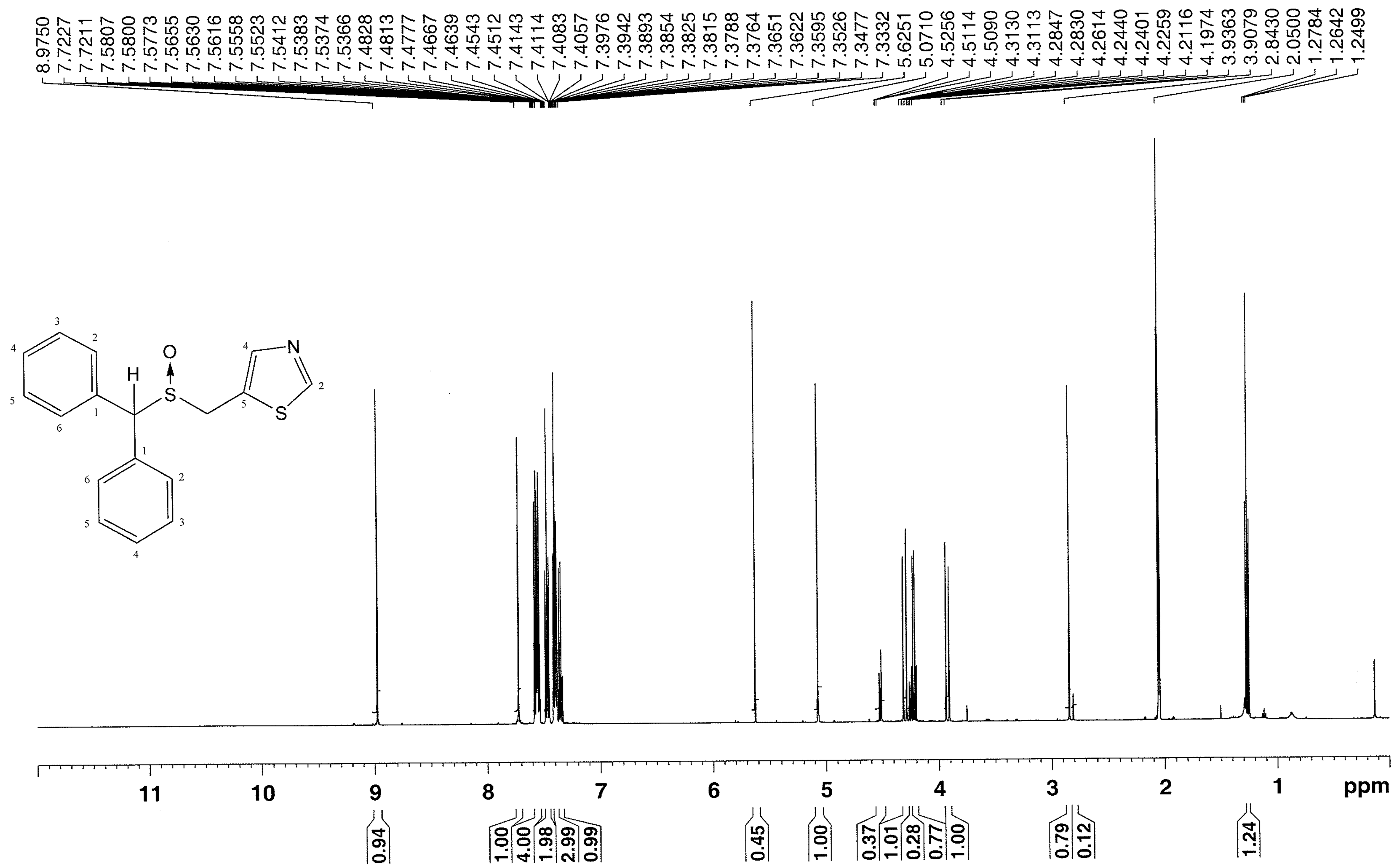

VD253 in d6Acetone (Proton) 28.9.2017

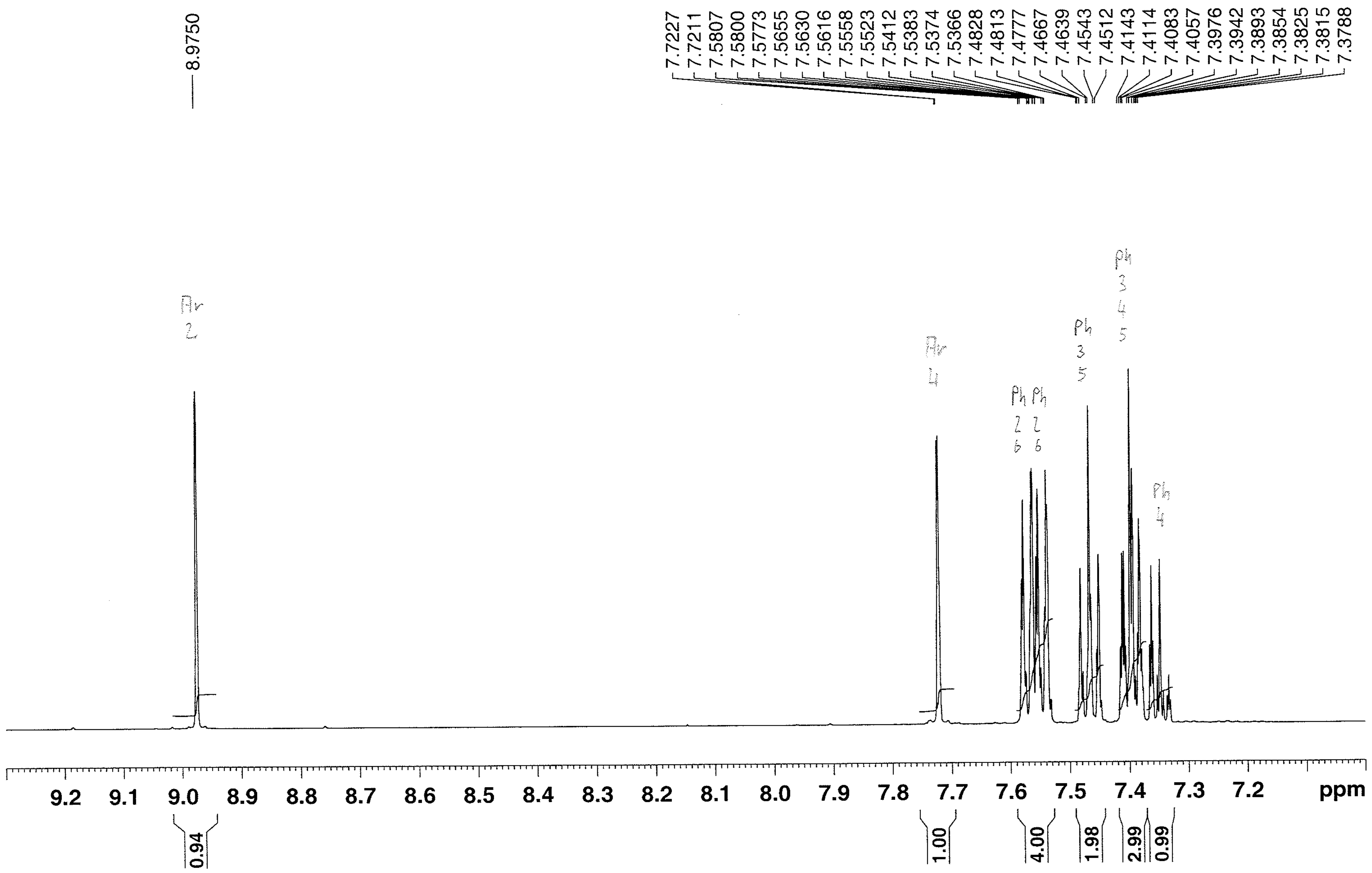

VD253 in d6Acetone (Proton) 28.9.2017

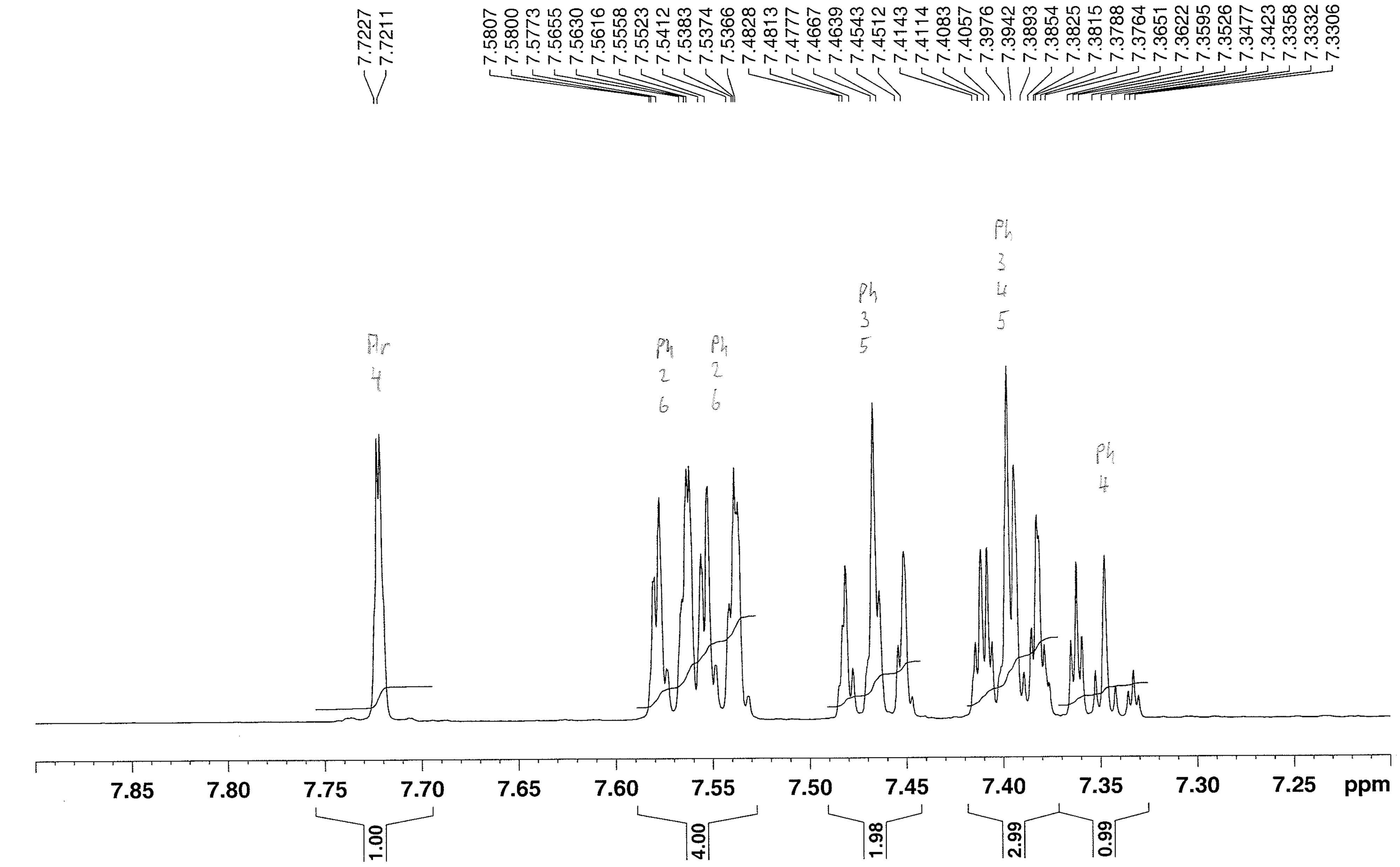

VD253 in d6Acetone (Proton) 28.9.2017

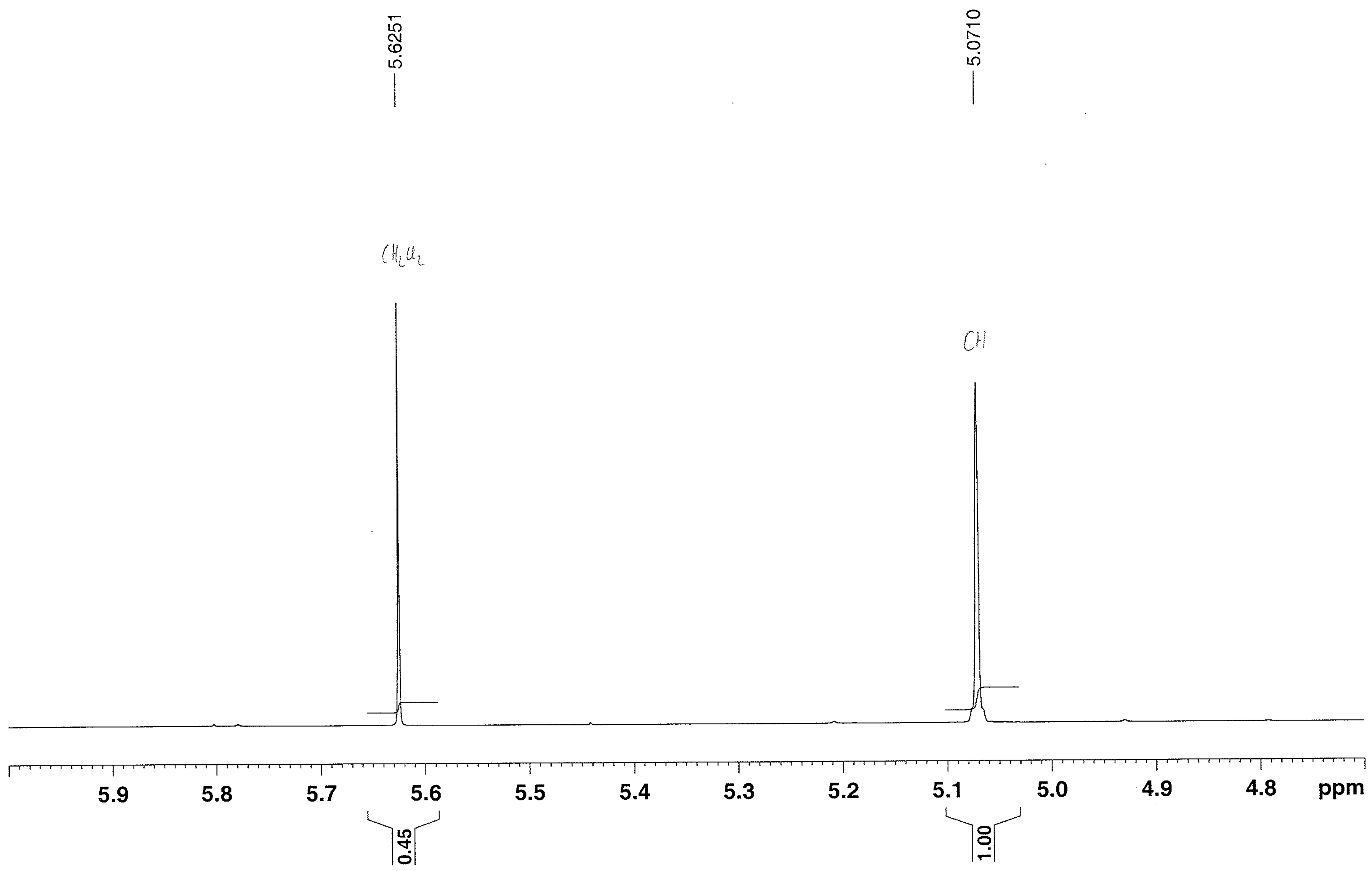

VD253 in d6Acetone (Proton) 28.9.2017

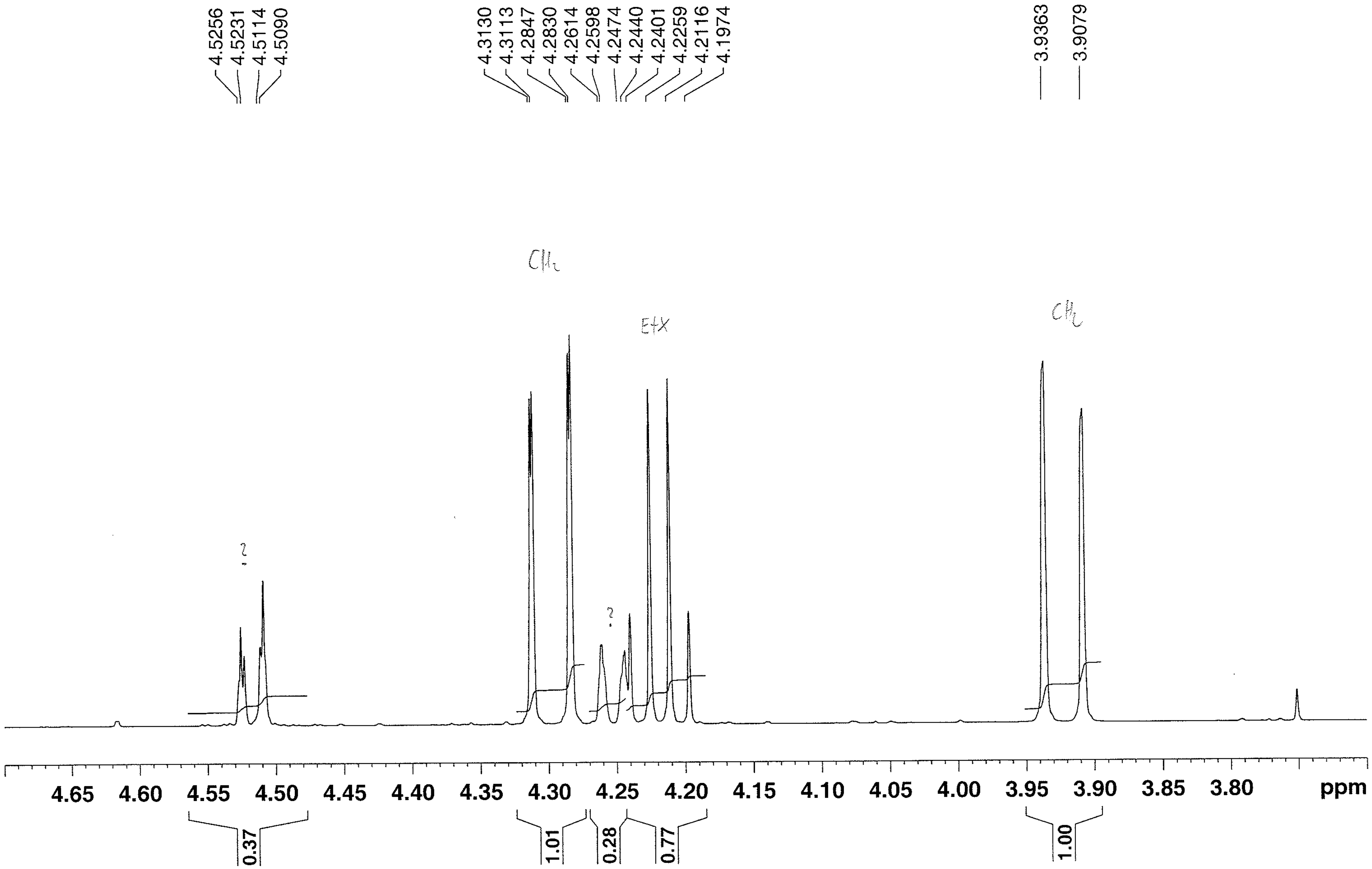

VD253 in d6Acetone (Proton) 28.9.2017

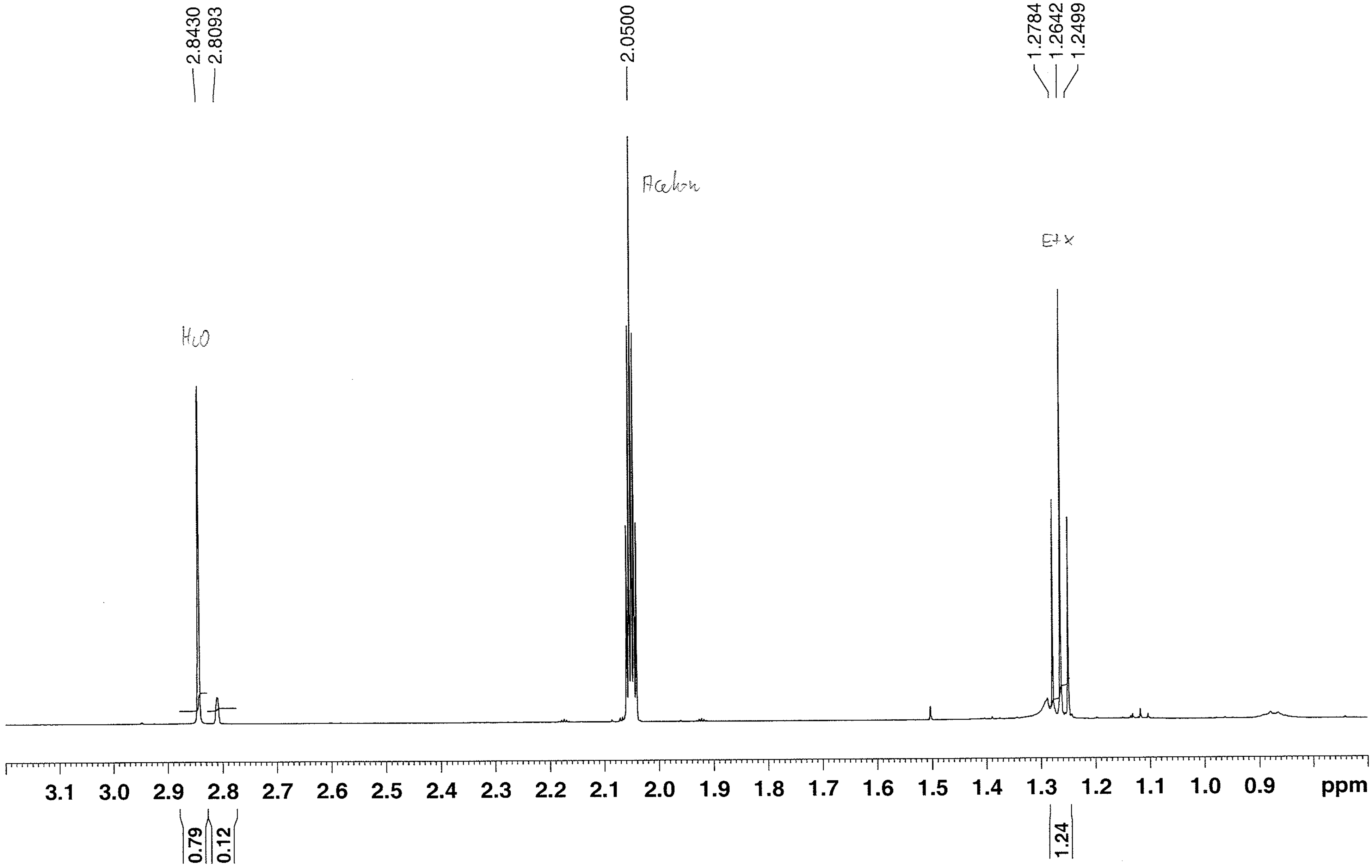

VD253 in d6Acetone (APT) 28.9.2017

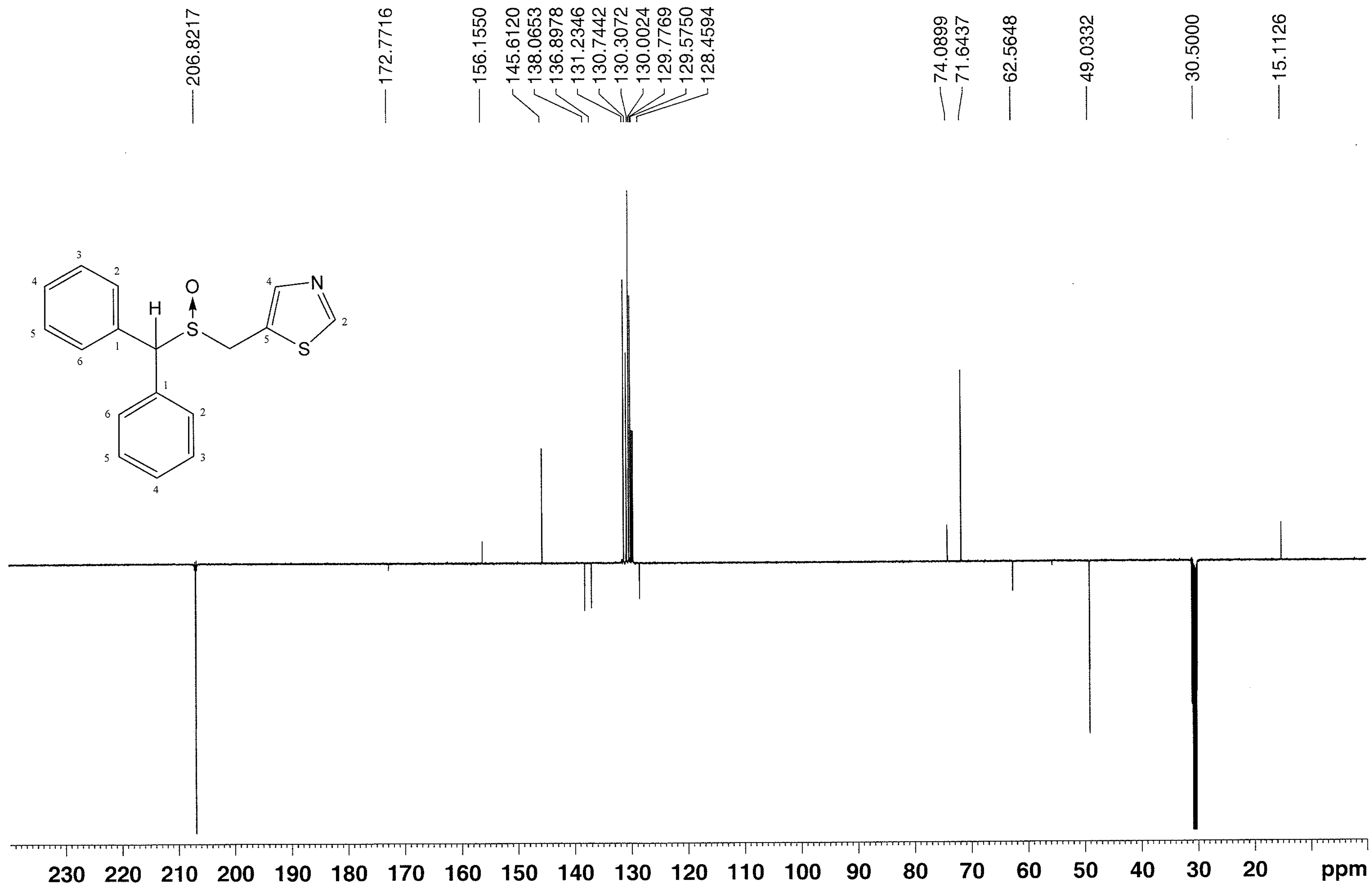

VD253 in d6Acetone (APT) 28.9.2017

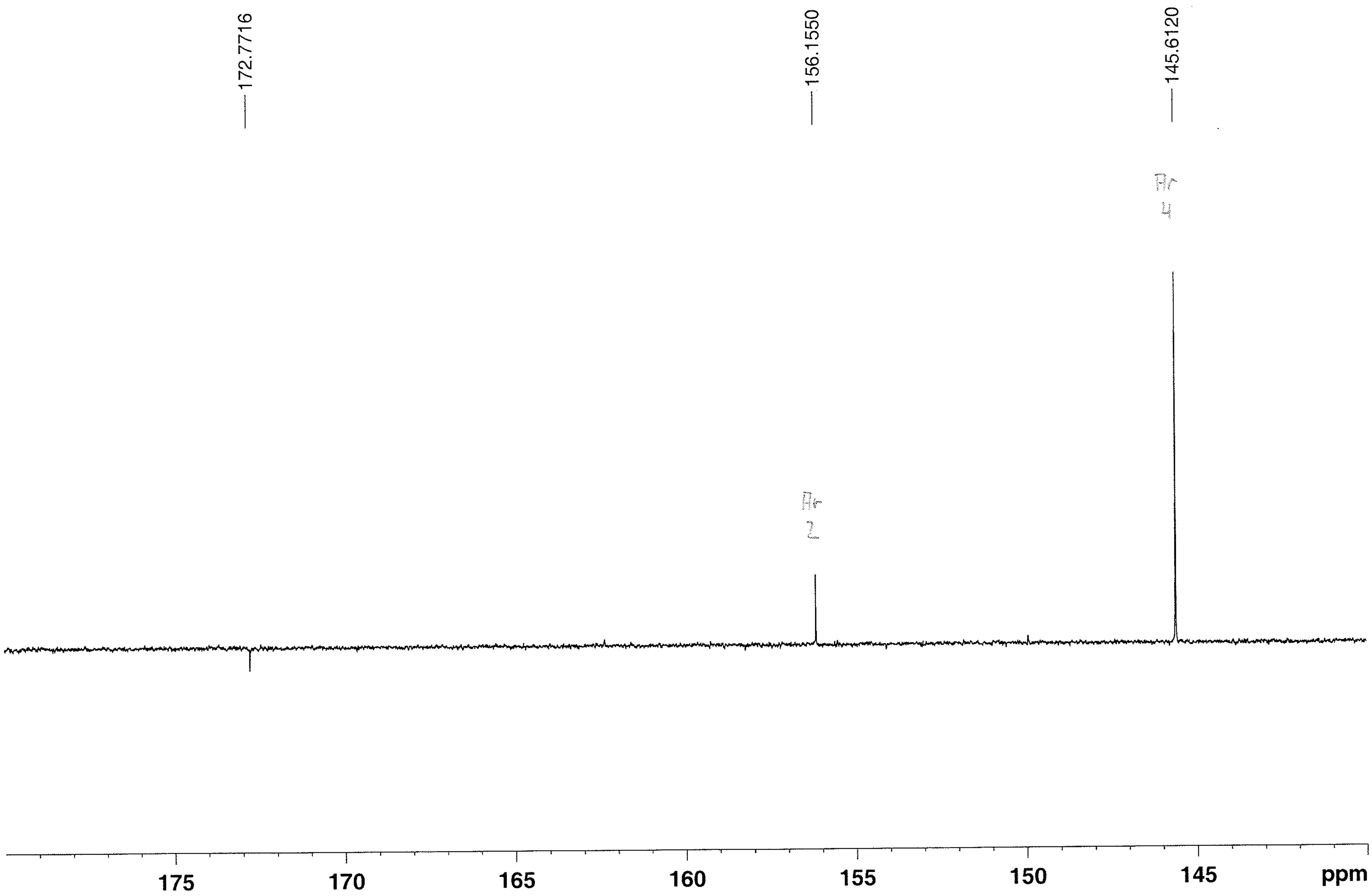

VD253 in d6Acetone (APT) 28.9.2017

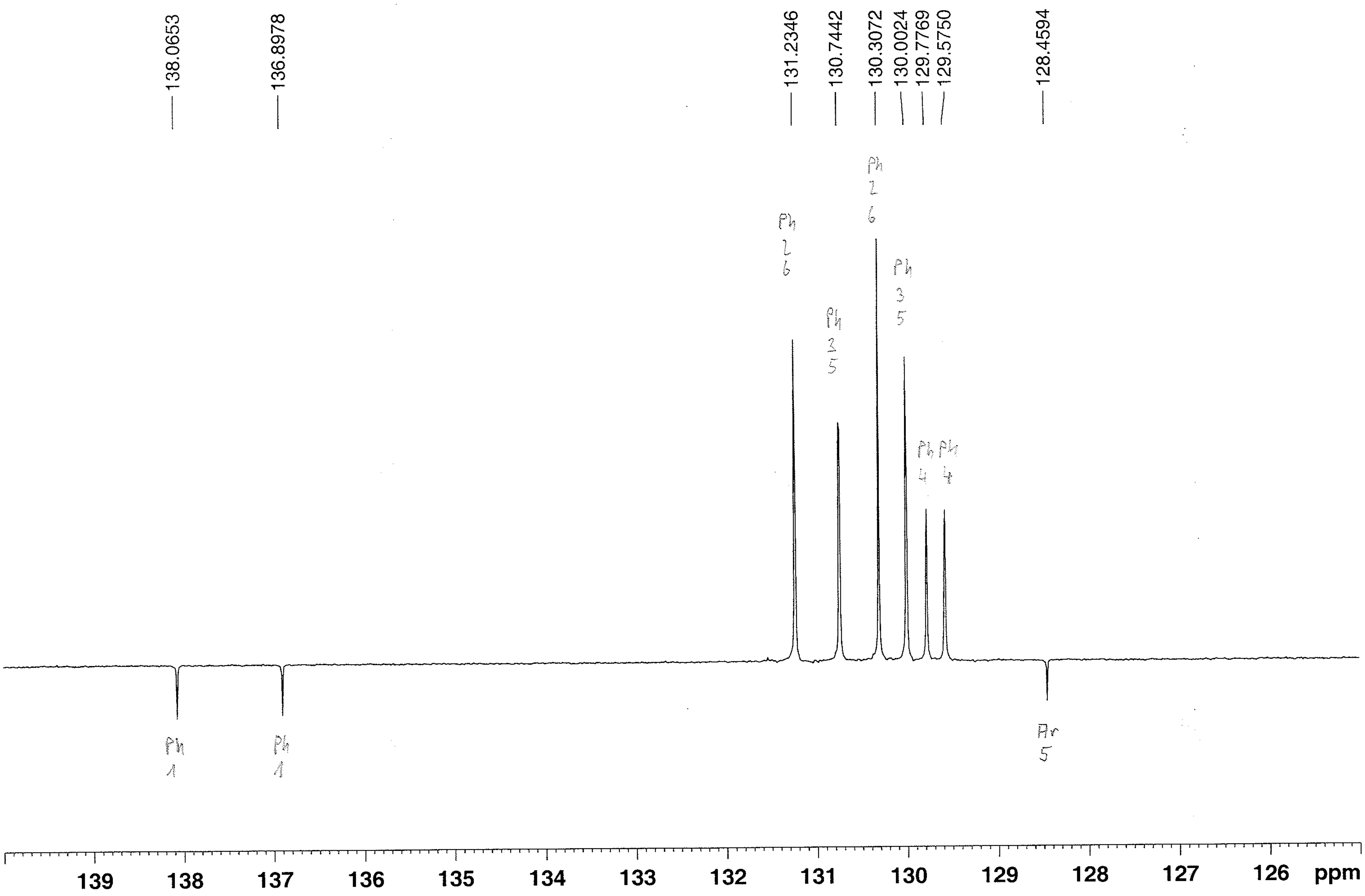

VD253 in d6Acetone (APT) 28.9.2017

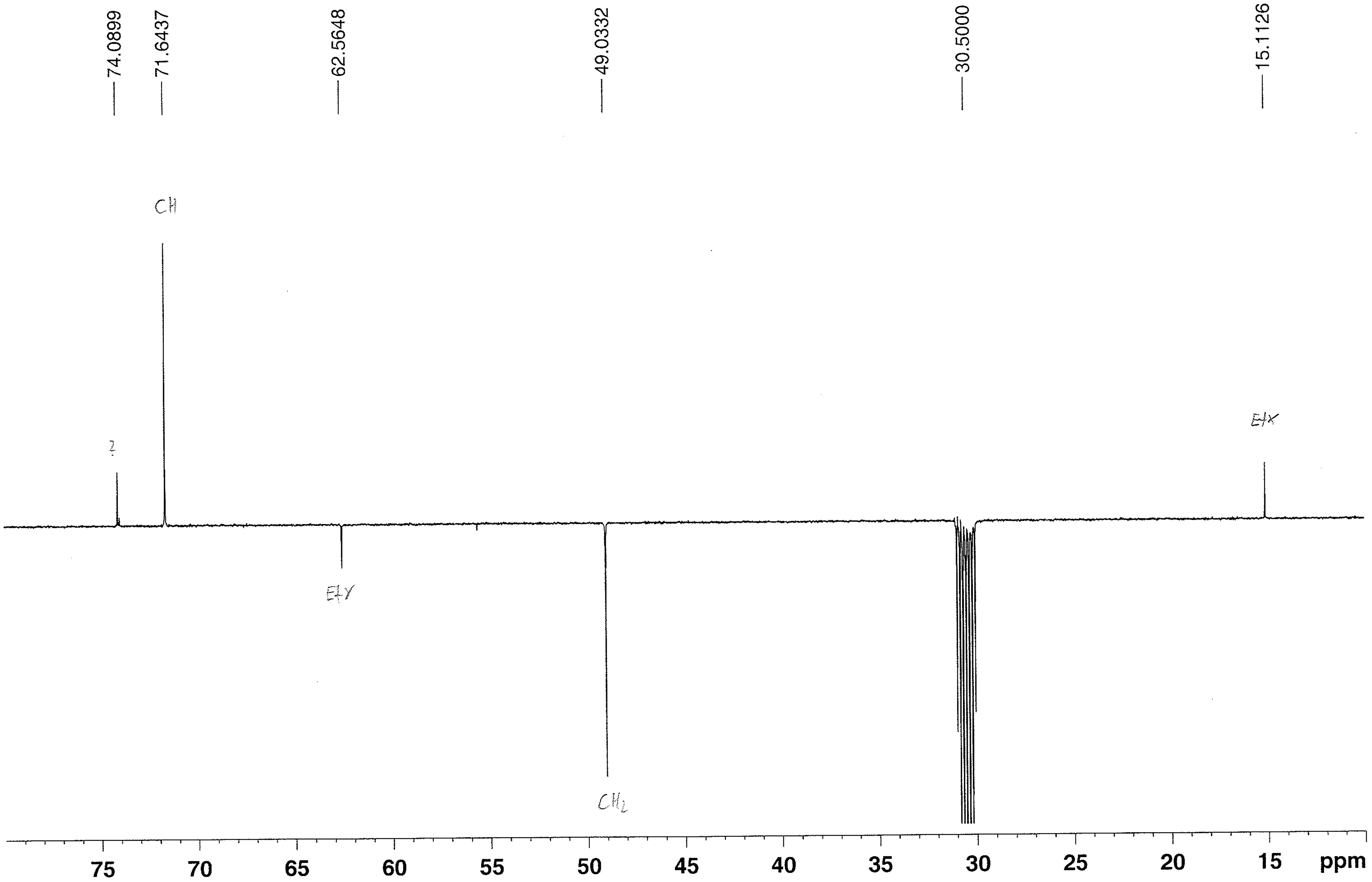

VD253 in d6Acetone (COSY) 28.9.2017

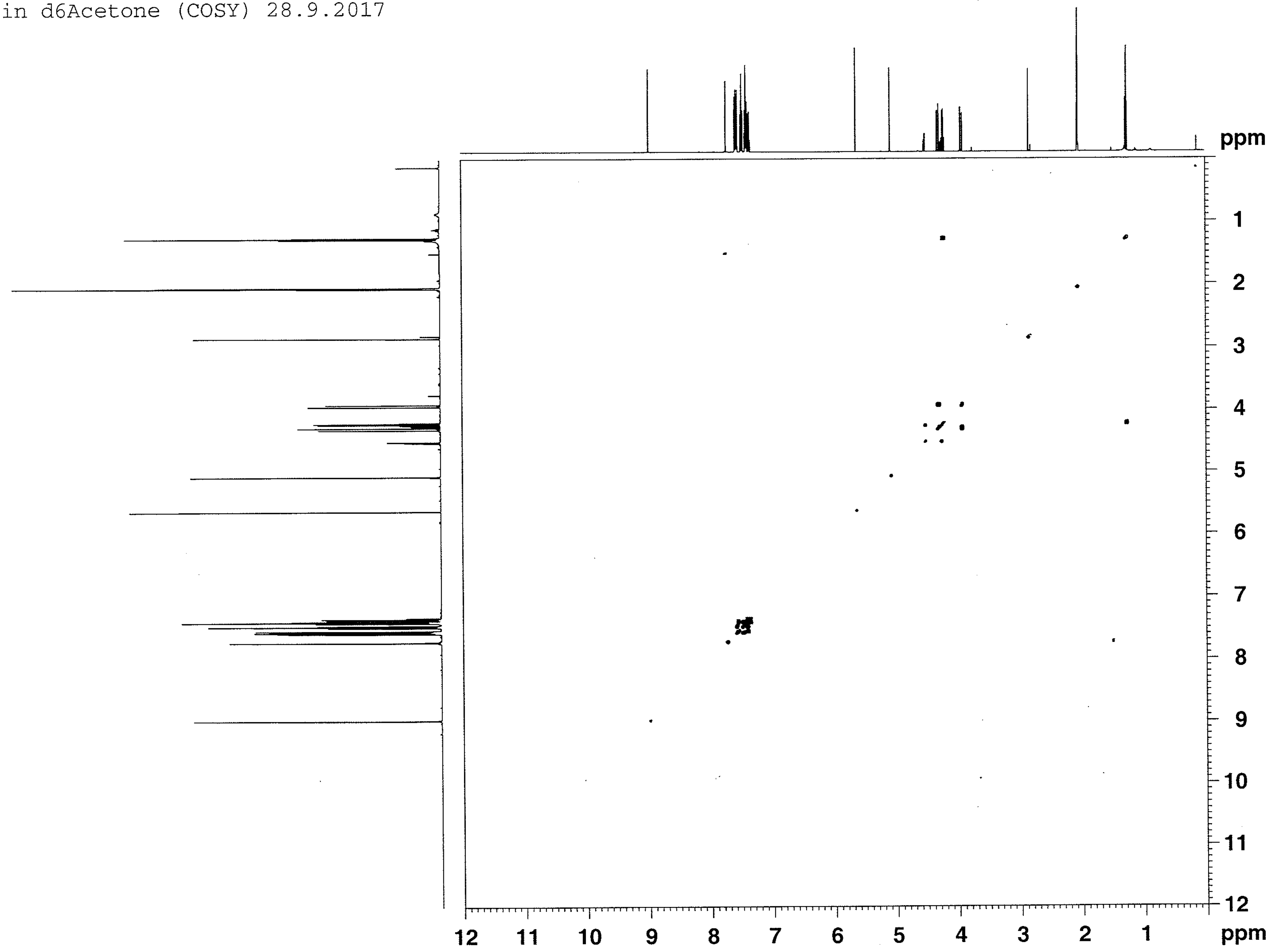

VD253 in d6Acetone (COSY) 28.9.2017

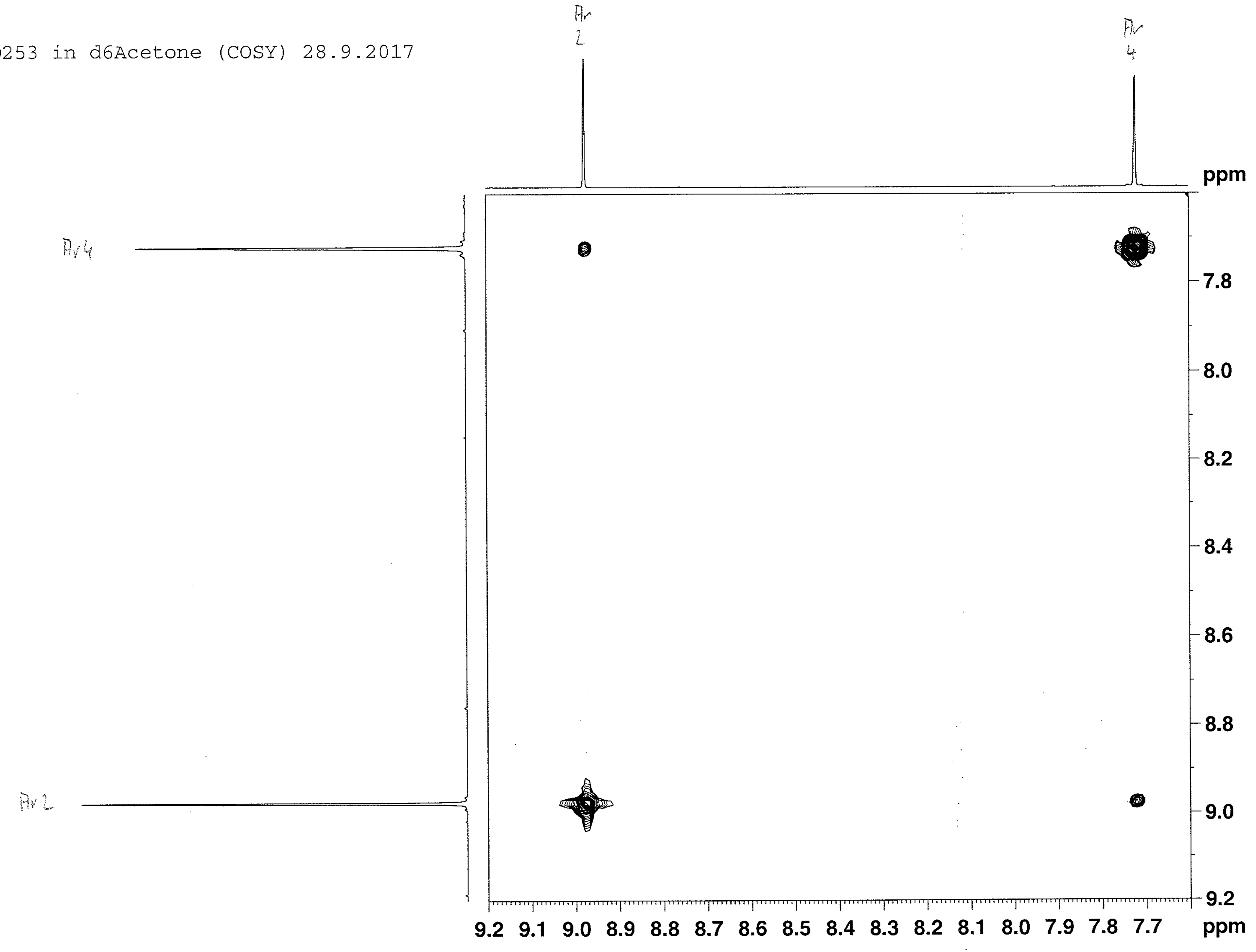

VD253 in d6Acetone (COSY) 28.9.2017

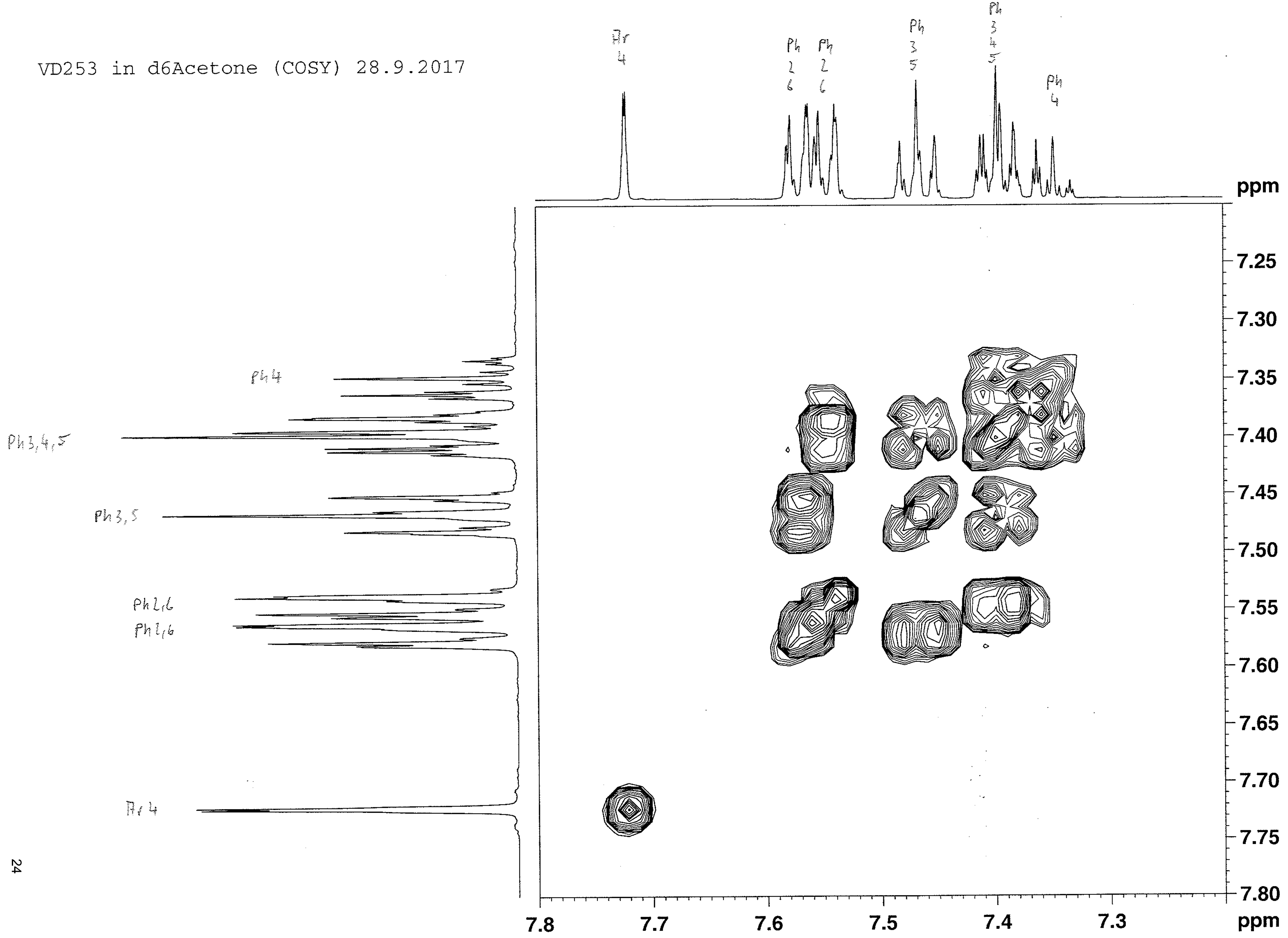

VD253 in d6Acetone (COSY) 28.9.2017

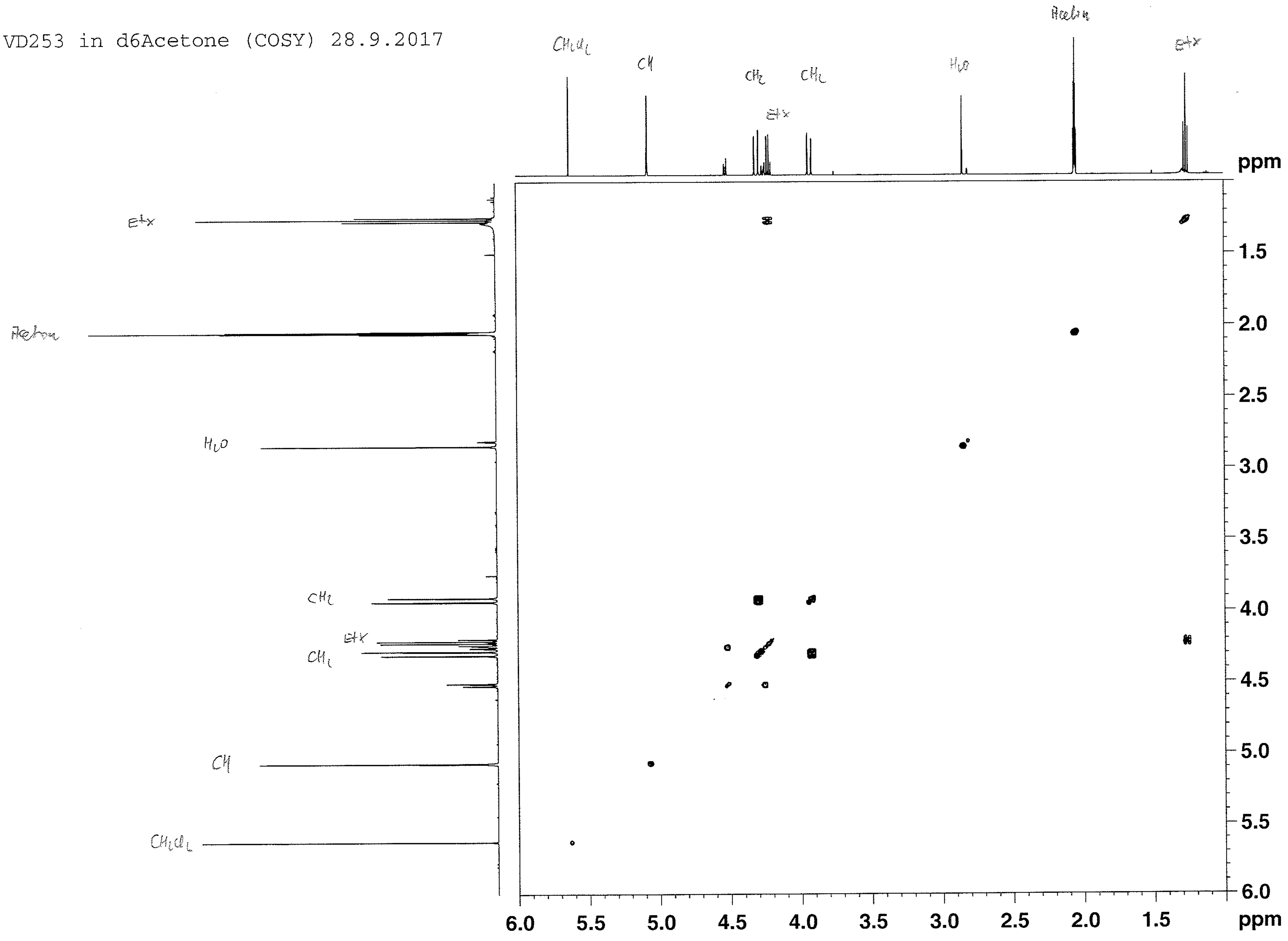

VD253 in d6Acetone (HSQC) 28.9.2017

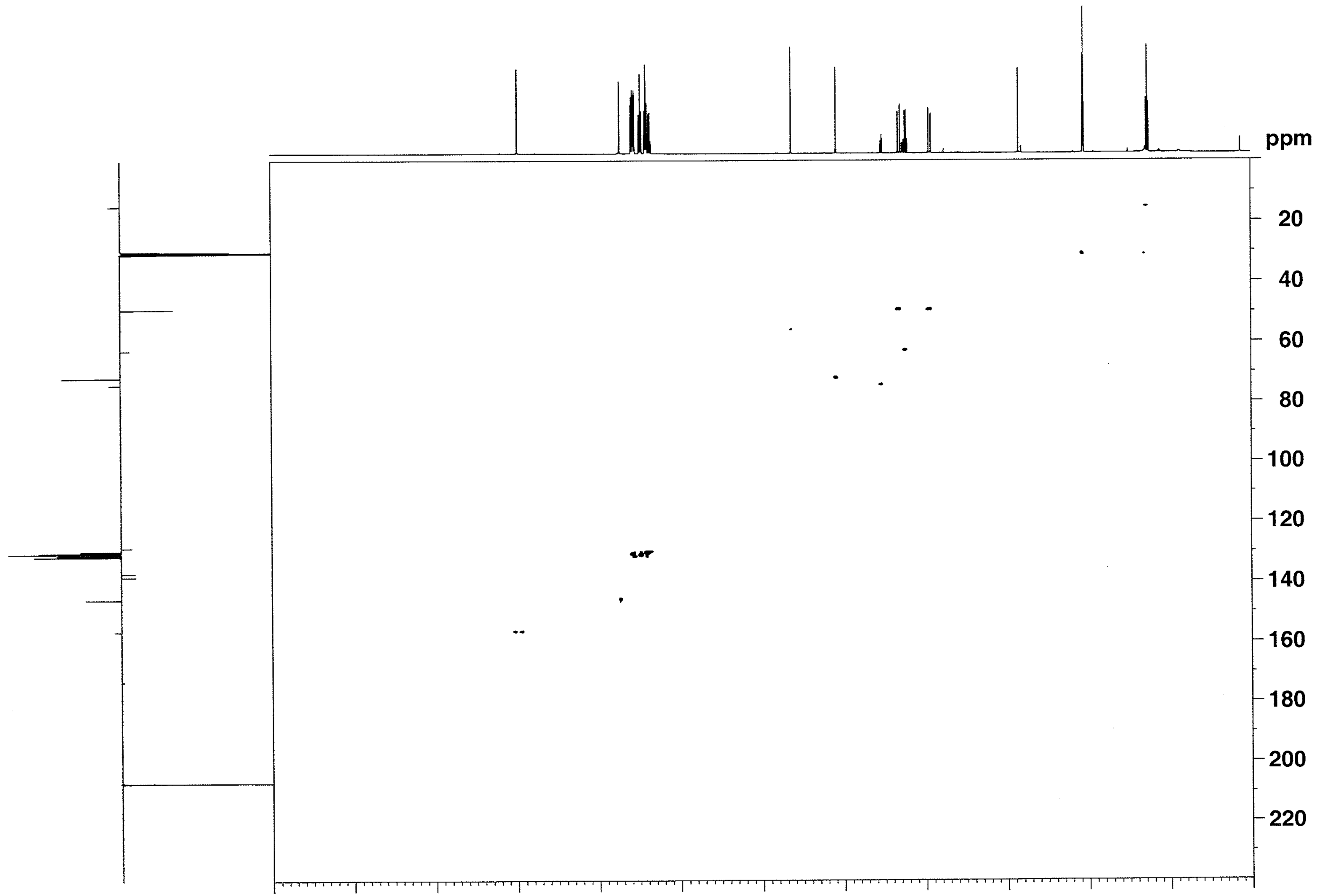

VD253 in d6Acetone (HSQC) 28.9.2017

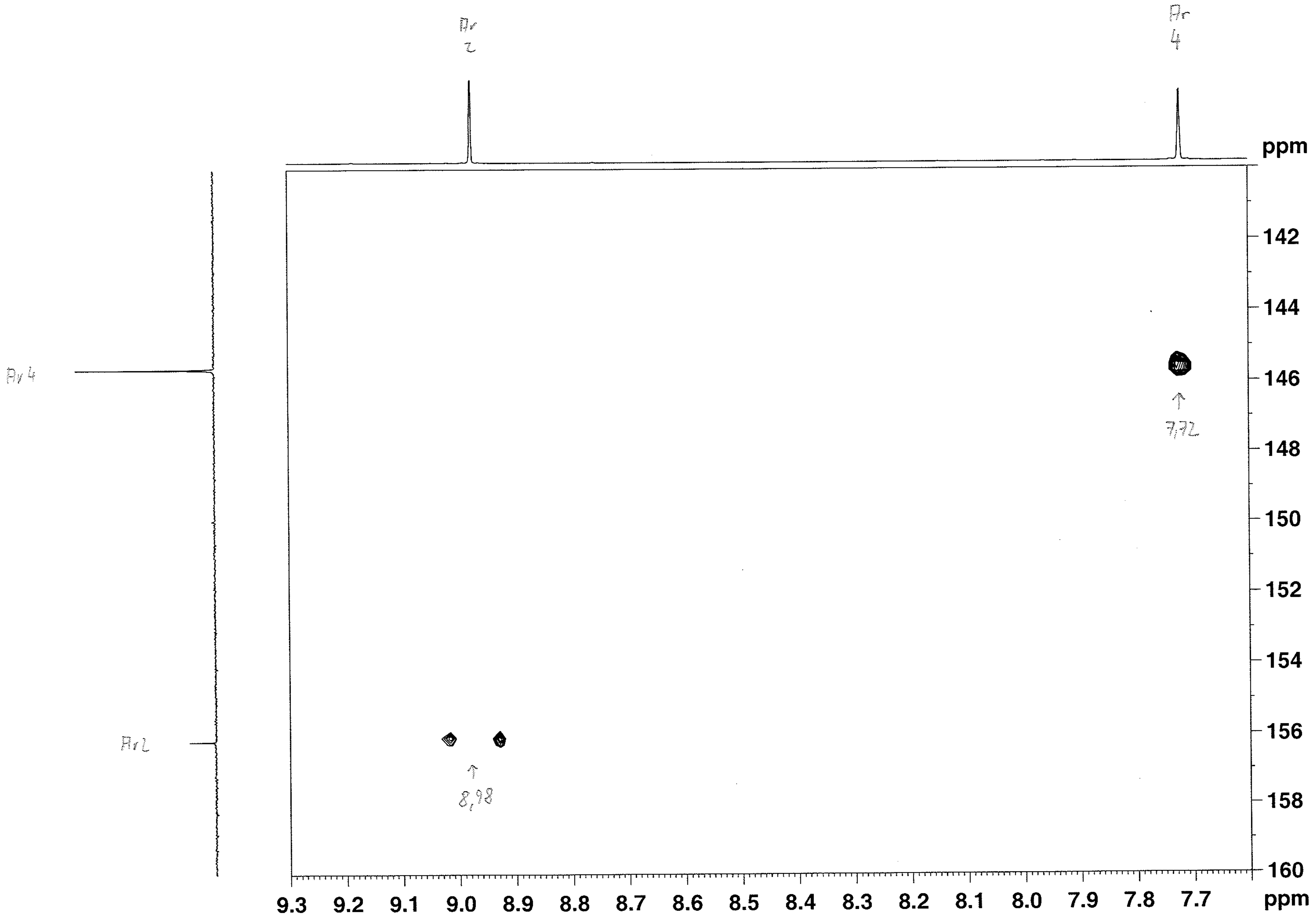

VD253 in d6Acetone (HSQC) 28.9.2017

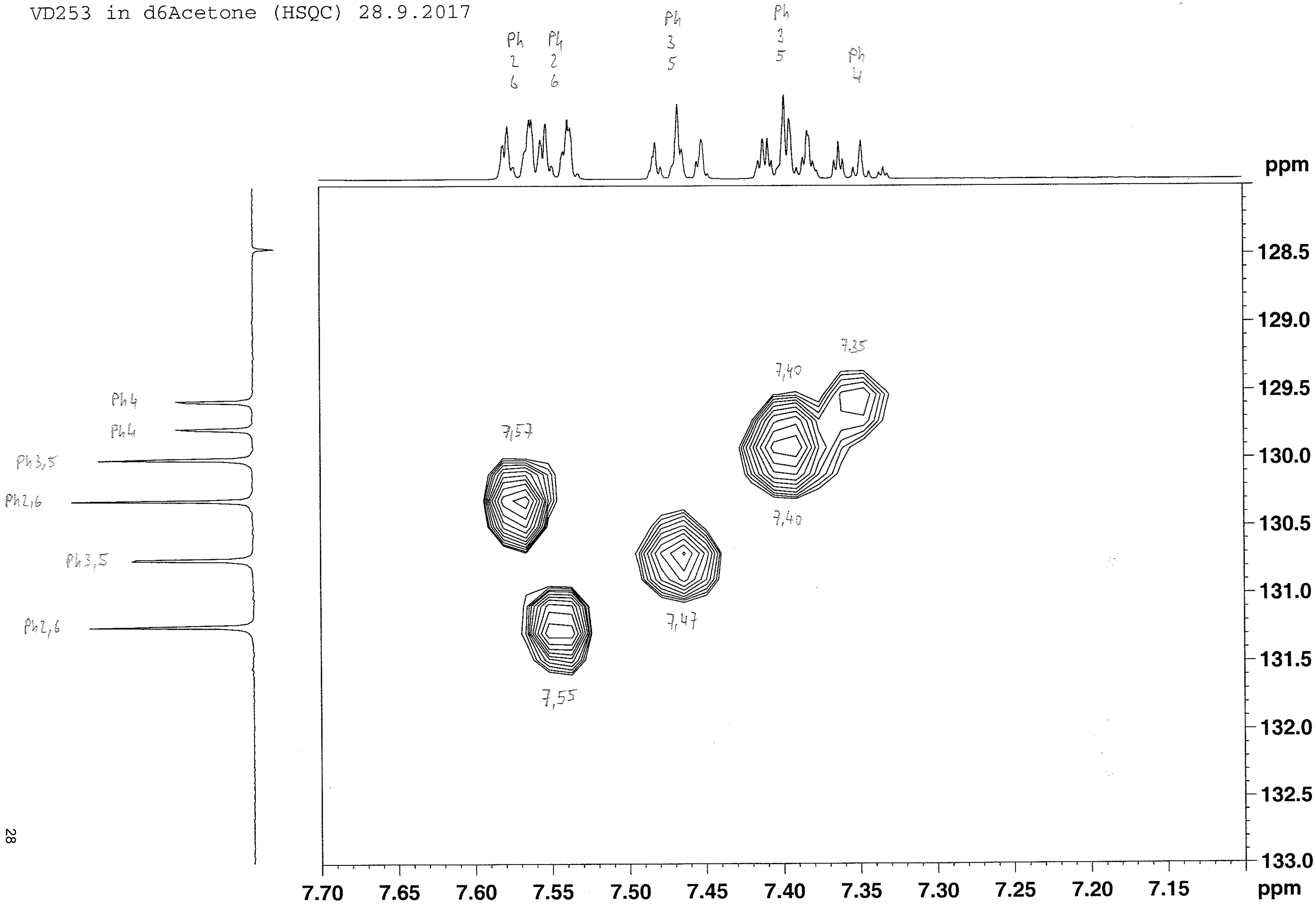

VD253 in d6Acetone (HSQC) 28.9.2017

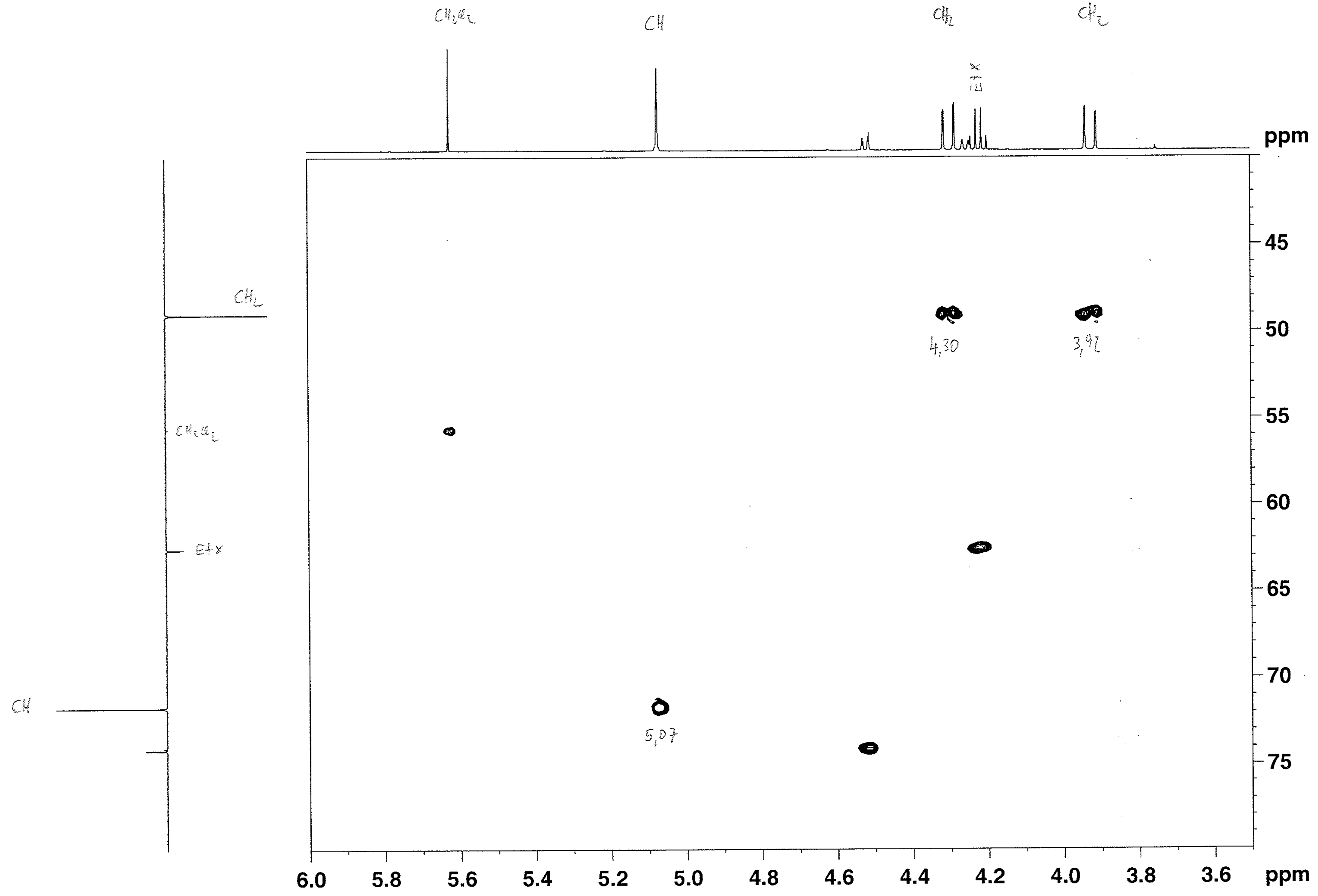

VD253 in d6Acetone (HSQC) 28.9.2017

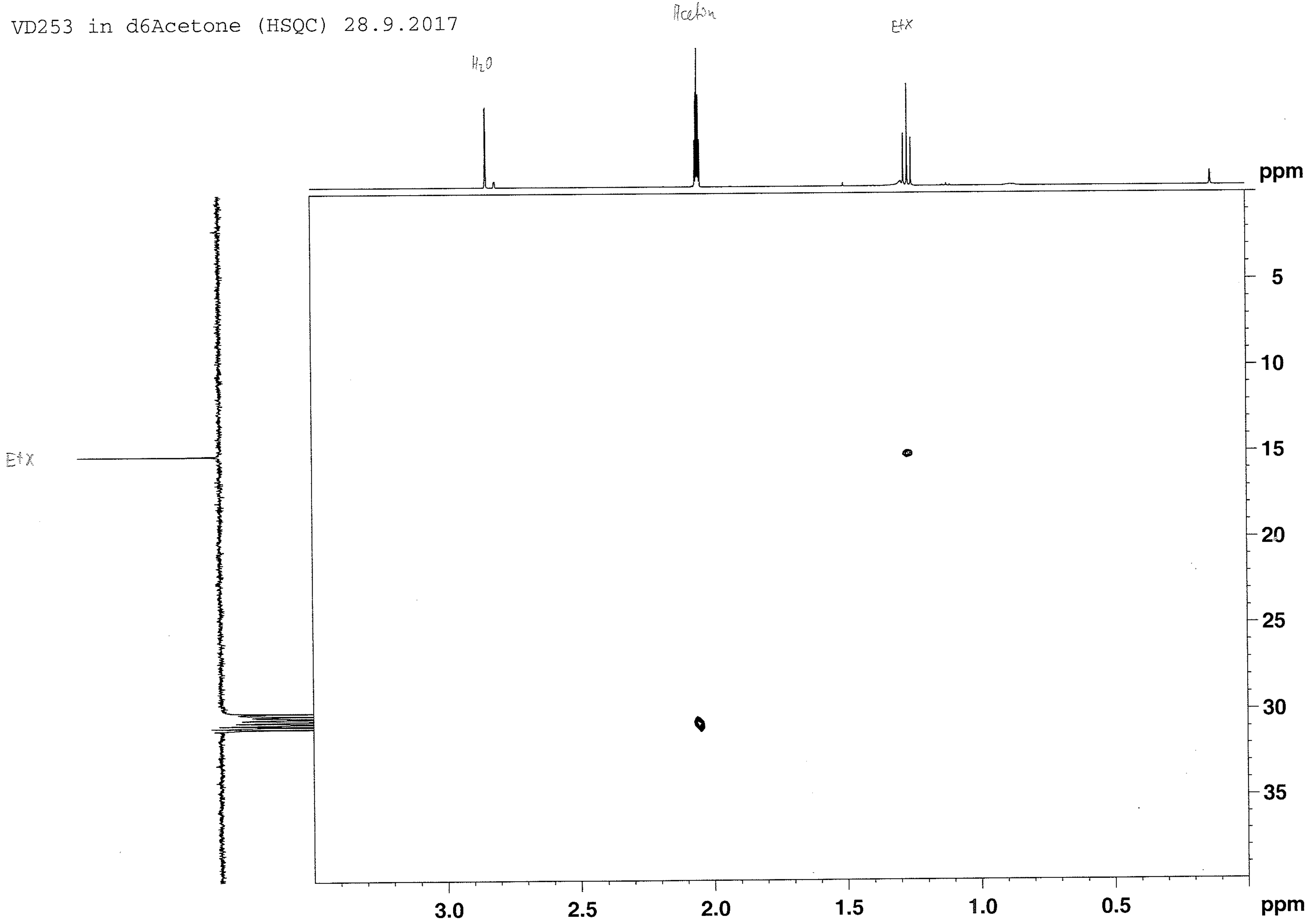

VD253 in d6Acetone (HMBC) 28.9.2017

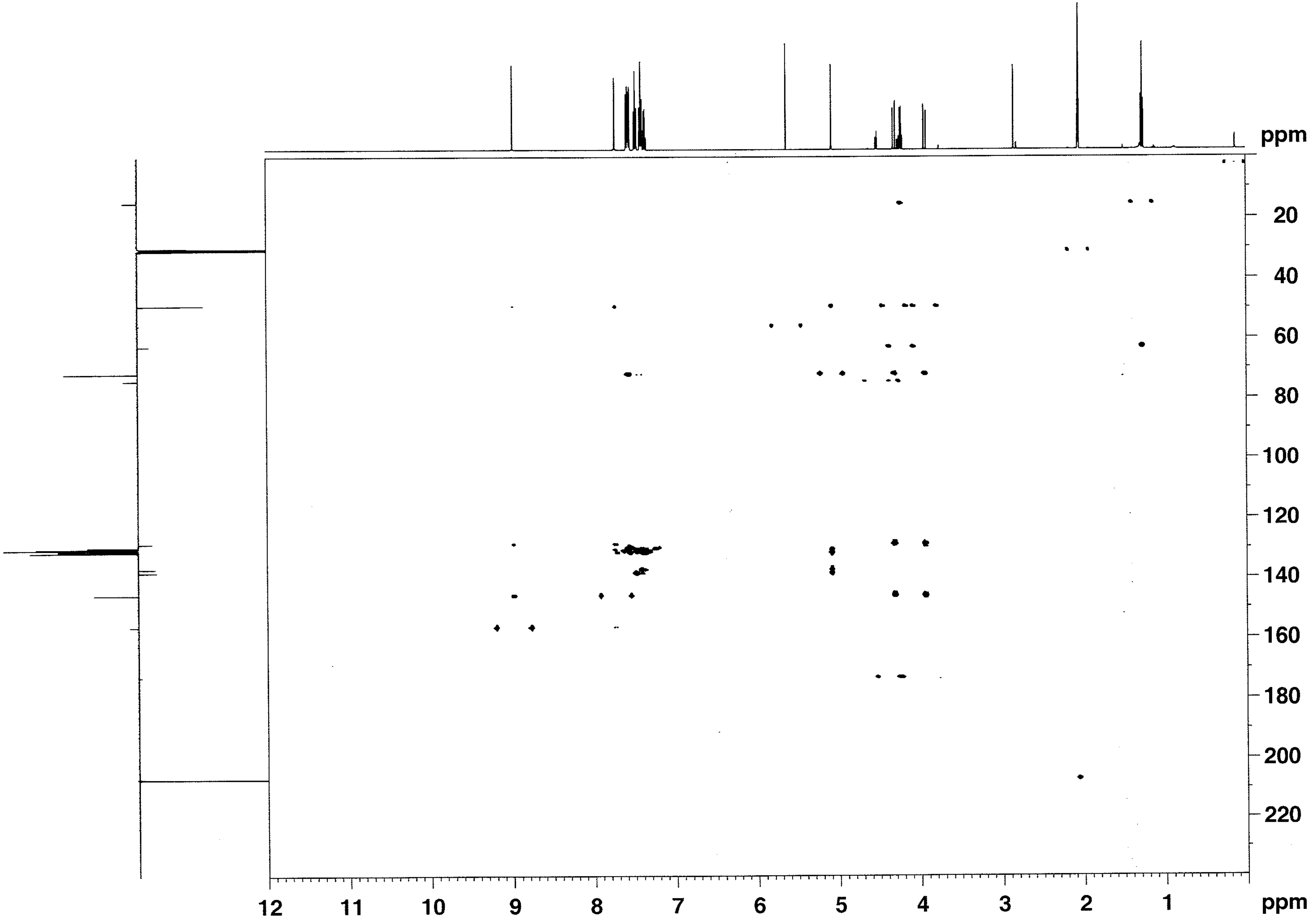

VD253 in d6Acetone (HMBC) 28.9.2017

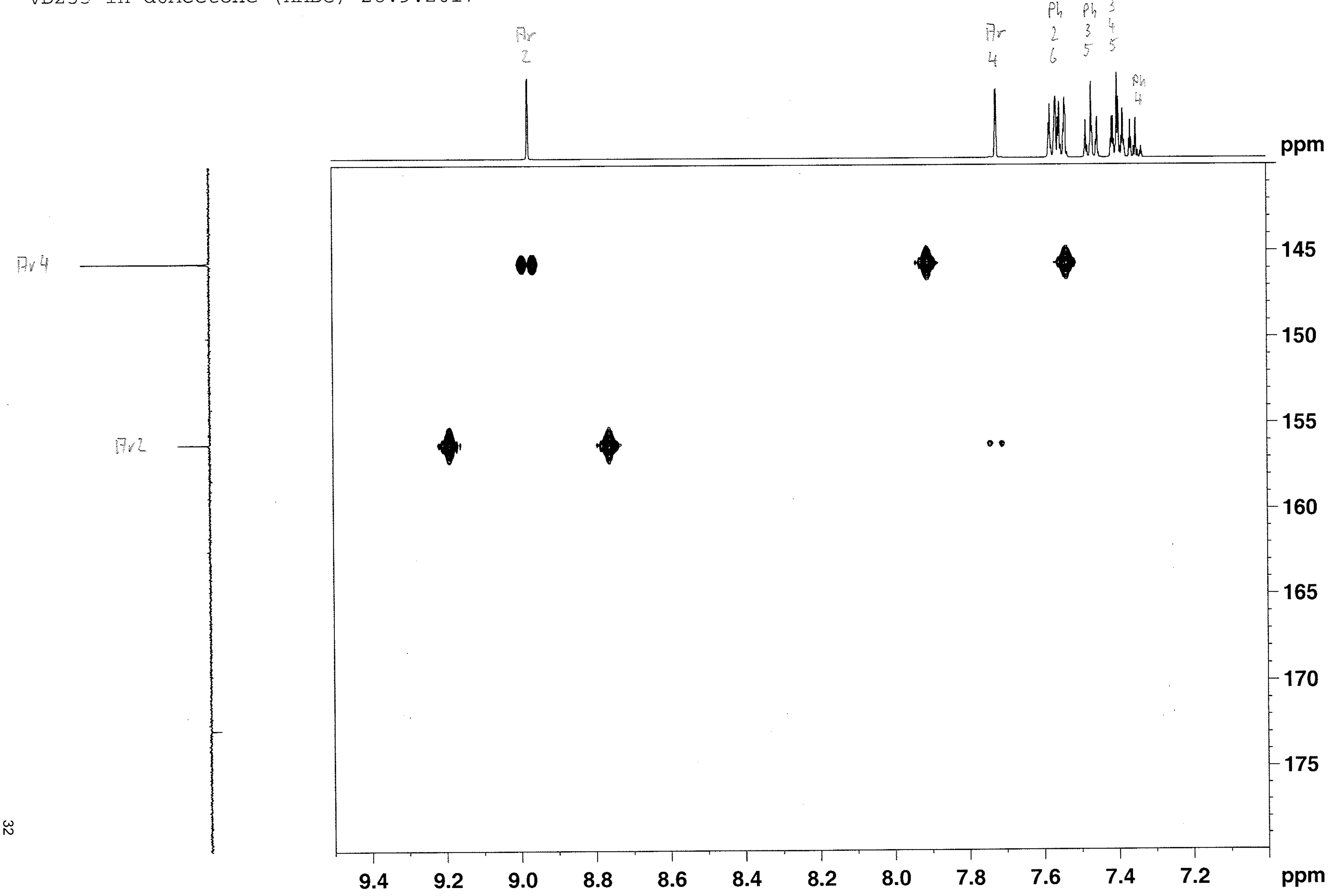

VD253 in d6Acetone (HMBC) 28.9.2017

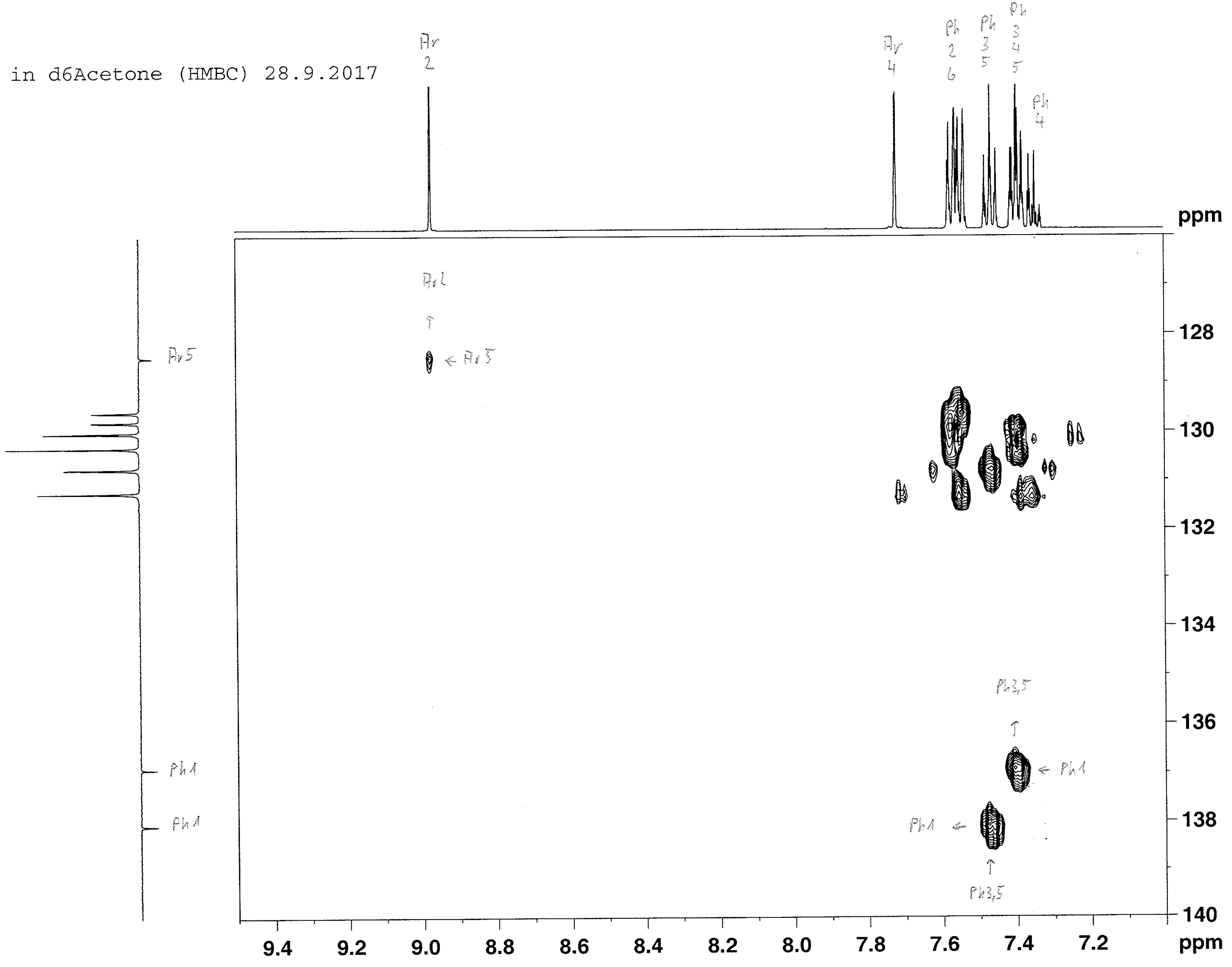

VD253 in d6Acetone (HMBC) 28.9.2017

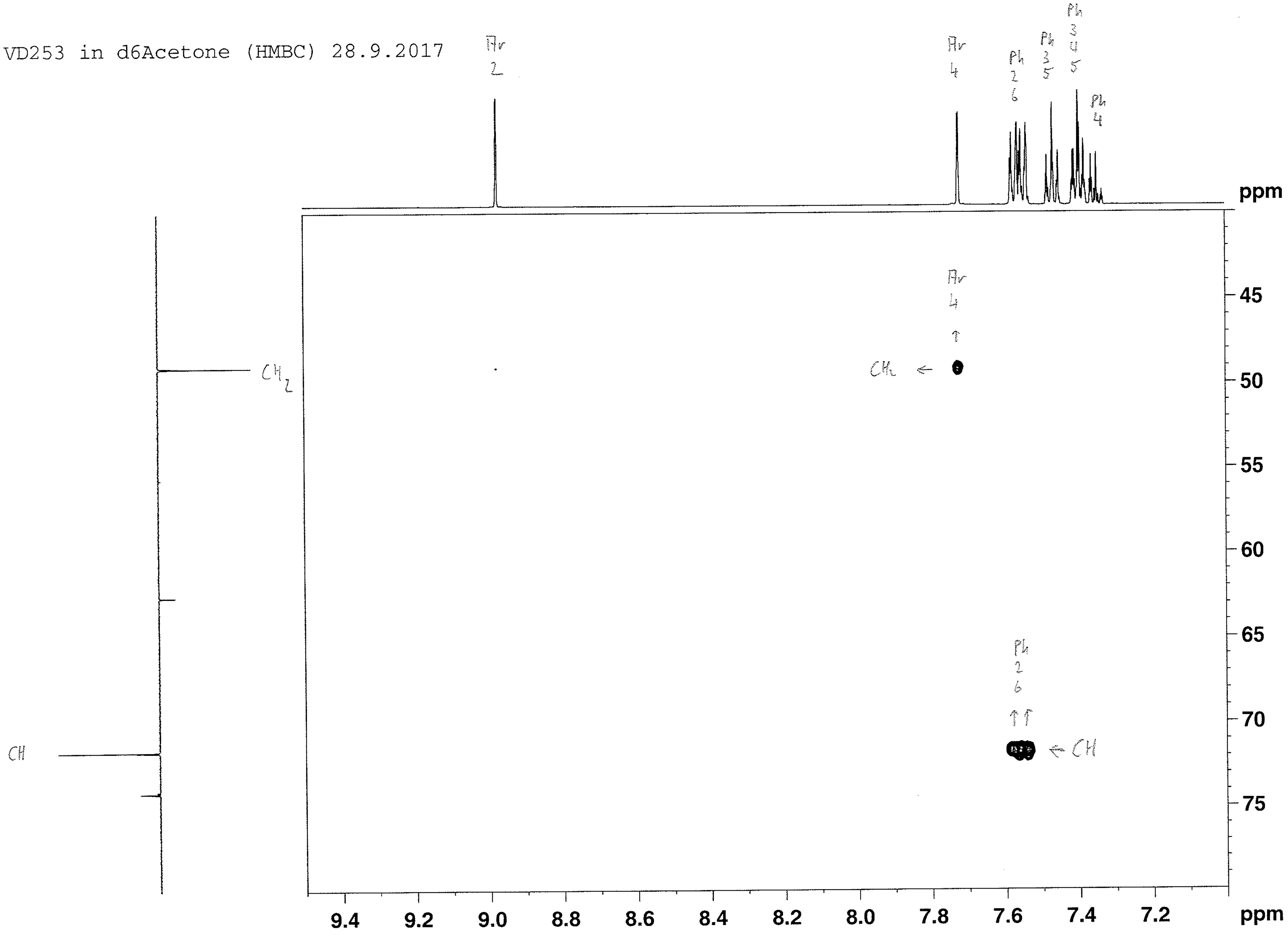

VD253 in d6Acetone (HMBC) 28.9.2017

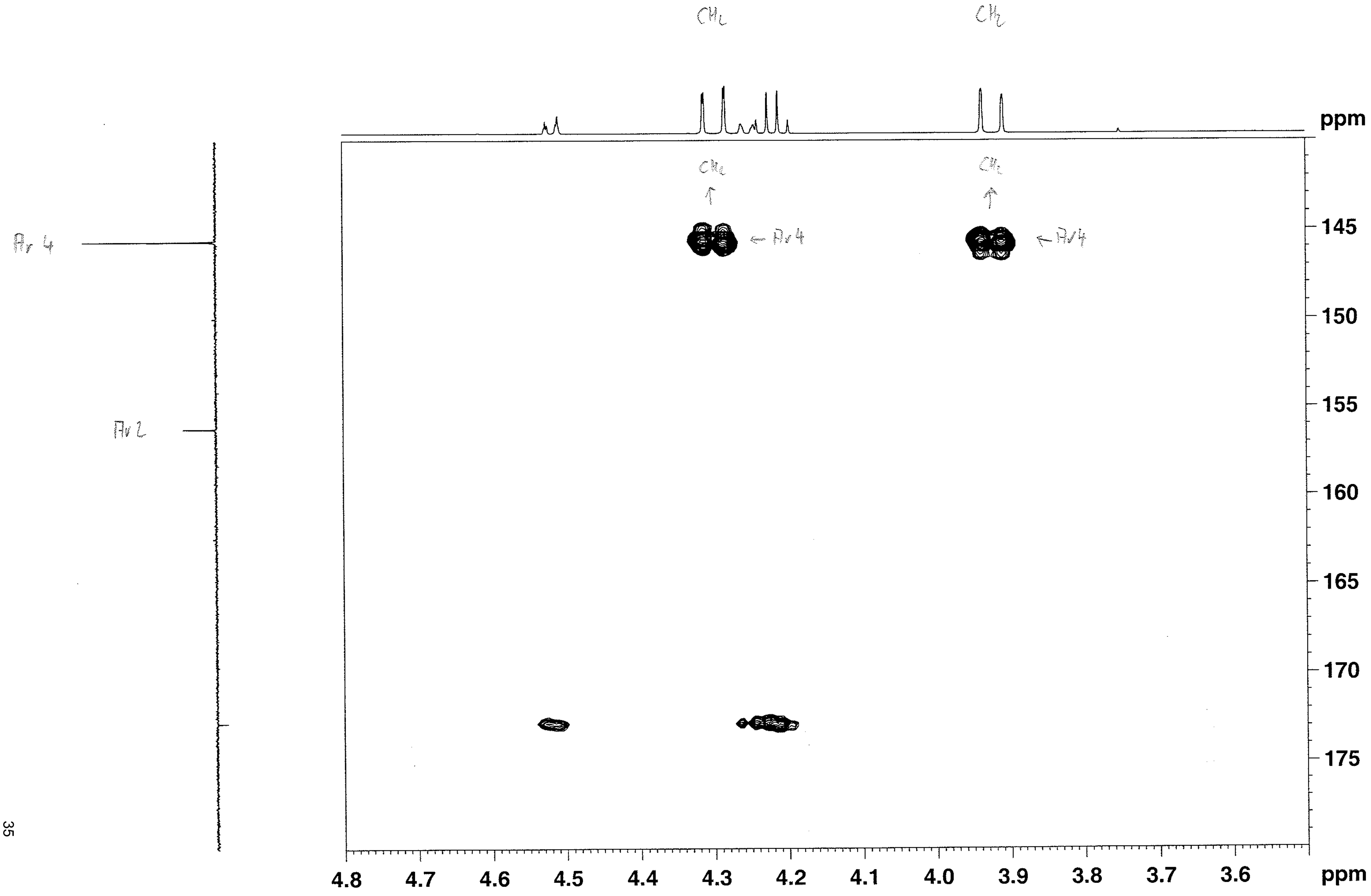

VD253 in d6Acetone (HMBC) 28.9.2017

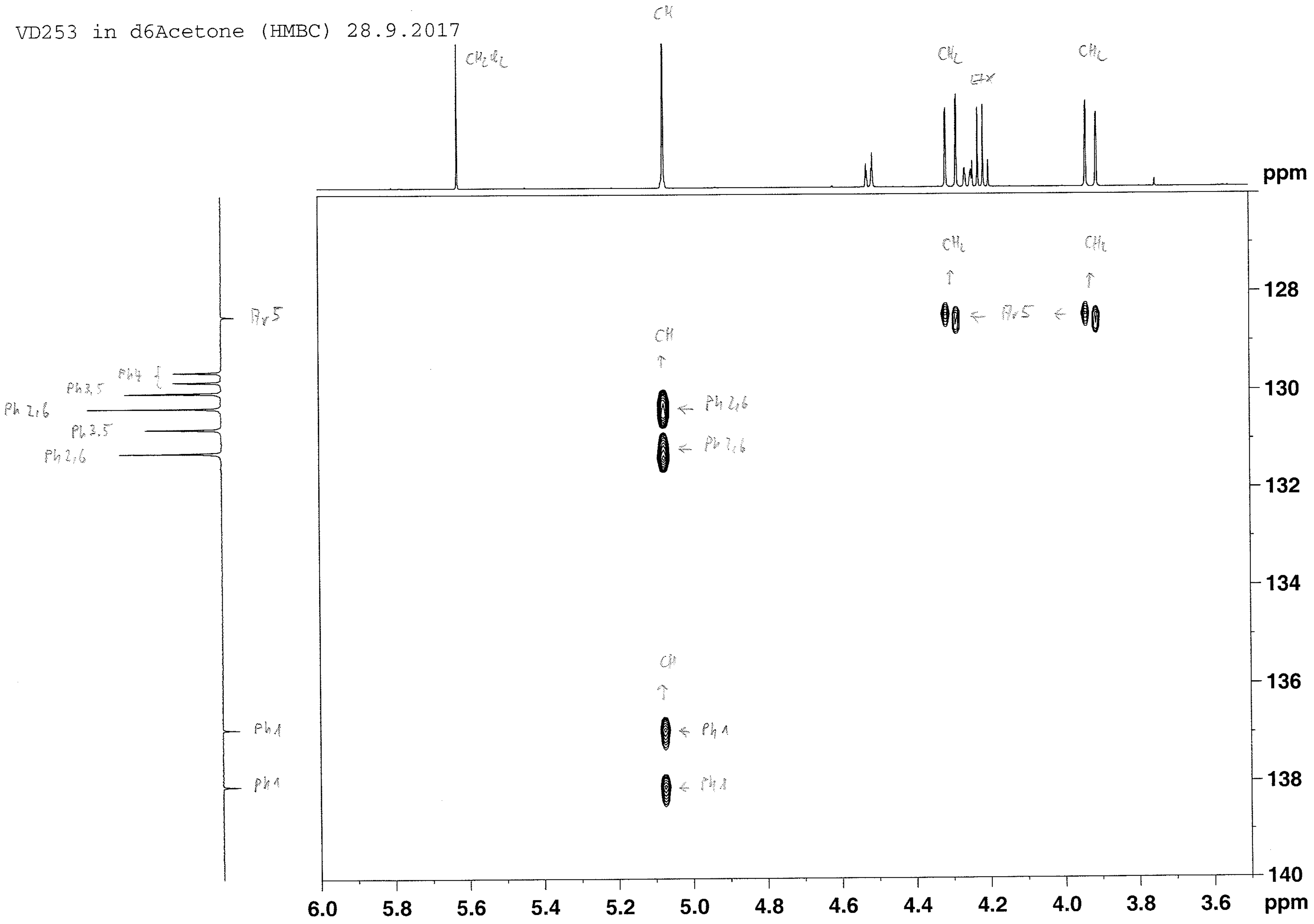

VD253 in d6Acetone (HMBC) 28.9.2017

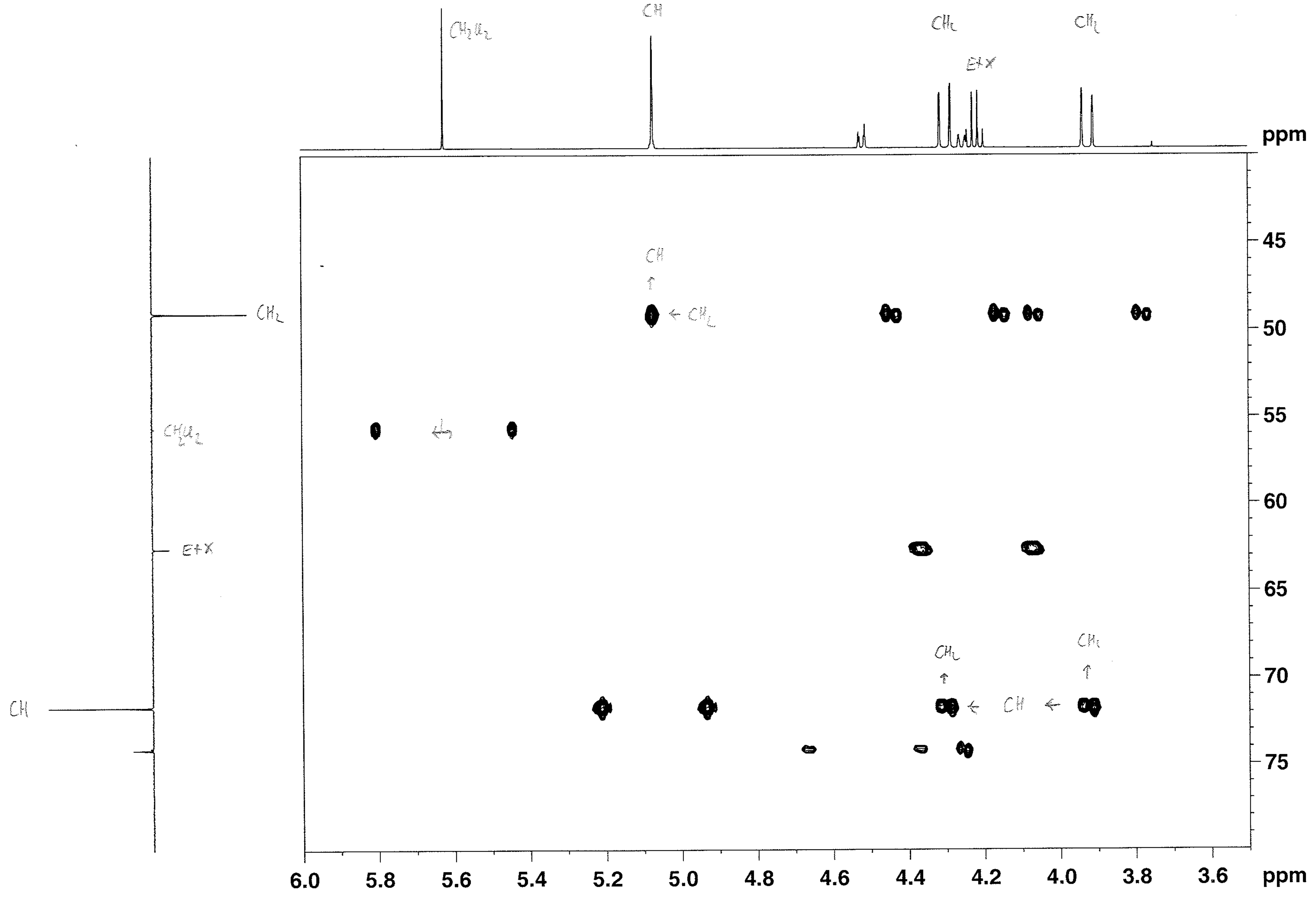

VD253 in d6Acetone (HMBC) 28.9.2017

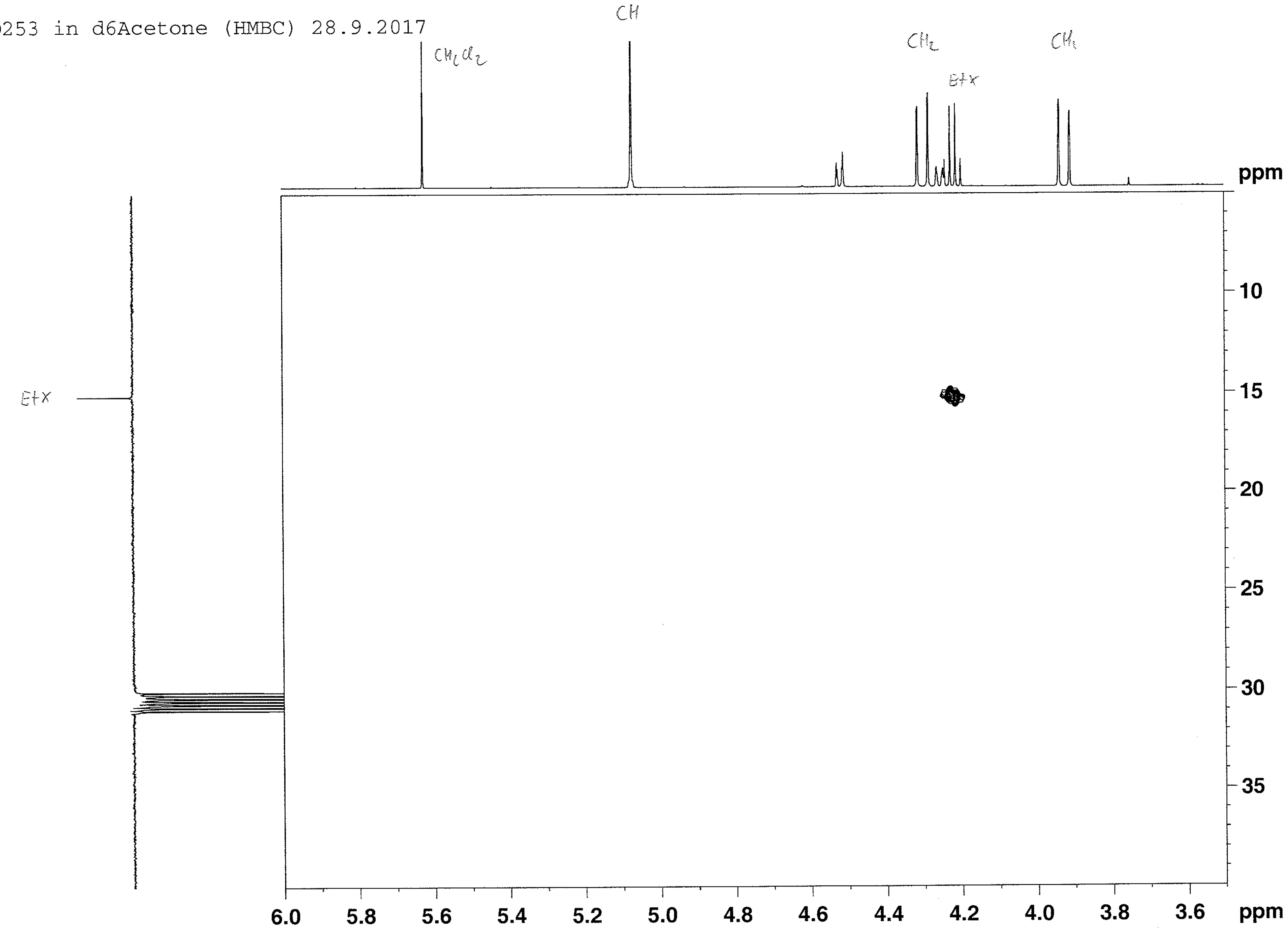

VD253 in d6Acetone (HMBC) 28.9.2017

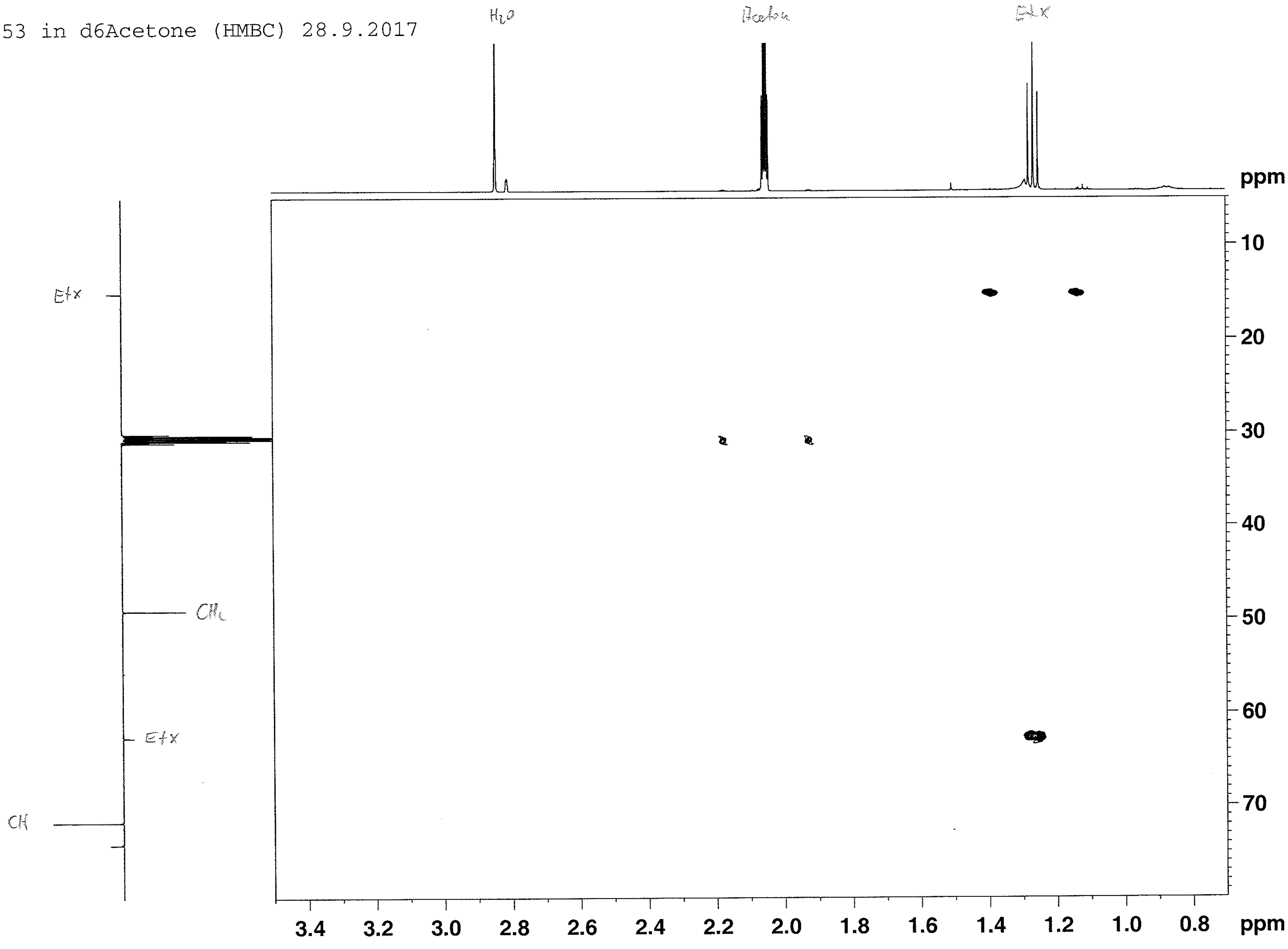

Title:

Absolute Configuration Determination Report

GENERAL INFORMATION	
**Customer**	University of Vienna<br>Department of Pharmaceutical Chemistry<br>Predrag Kalaba
**Sales Order Number**	N/A
**Sample code (Our ref.)**	**S17130 / S17131**
**Sample description (Your ref.)**	S17130 = CE-123A<br>S17131 = CE-123B
**VCD-spectrometer**	ChiralIR-2X w/ Dual*PEM*
**Report prepared by**	Wouter Herrebout
**Report validated by**	Wouter Herrebout
**Report signed by**	NA
**Date**	May, 20th, 2017

RESULTS	
Absolute Configuration of **S17130: (*S*)**<br>Absolute Configuration of **S17131: (*R*)**	Confidence Level: **99 %**

MEASUREMENT PARAMETERS	
**Concentration**	4.1 mg / 100 µL
**Solvent**	$CDCl_3$
**Resolution**	4 cm^{-1}
**PEM setting**	1400 cm^{-1}
**Number of scans/Measurement time**	75.000 scans / 24.0 hours
**Sample cell**	BaF_2
**Path length**	100 µm

CALCULATION DETAILS	
**Force fields used in MolMec conformational analyses**	MMFF94S, MMFF, SYBYL
**Number of conformations generated**	18
**Methodology and basis set for DFT calculations**	SCRF-B3LYP/6-31G(d)<br>SCRF-B3PW91/6-31G(d)
**Enantiomer used for calculation**	(*S*)
**Number of conformations used in calculated spectrum**	11
**Number of low-energy conformations shown in report**	2

COMMENTS

VCD spectra were recorded using $CDCl_3$ as a solvent. Baseline corrections were introduced by using spectra of both enantiomers.

Calculations were performed using B3LYP and B3PW91 functionals, to check for consistency.

17546 Bee Line Hwy | Jupiter, Florida 33458, USA
ph. 561 625.0133 | fax. 561 625.0717 | www.btools.com

Title:

Absolute Configuration Determination Report

Molecular Formula: $C_{17}H_{15}NOS_2$

Chemical Structure:

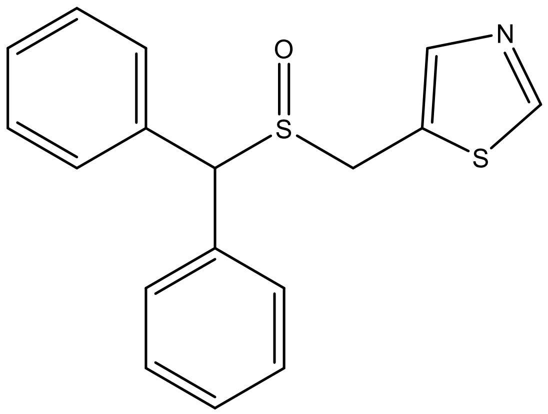

Absolute Configuration Determination Report

Experimental IR and VCD spectra for S17130

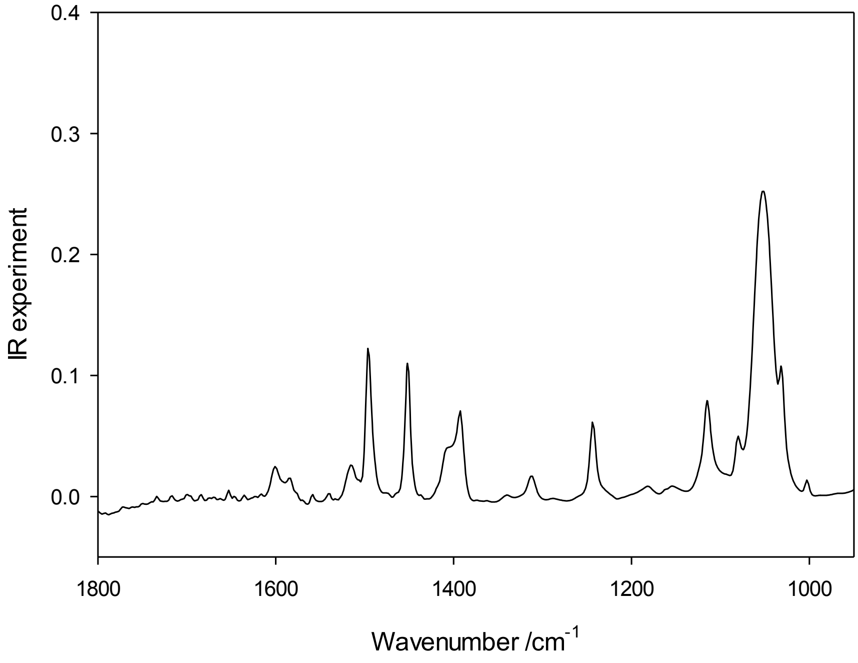

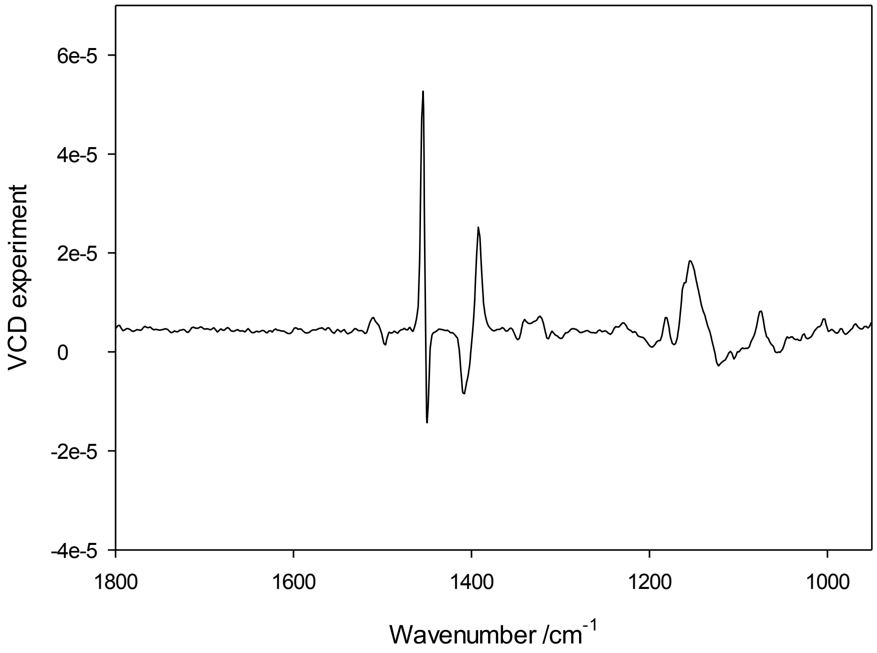

Absolute Configuration Determination Report

Optimized geometries and relative populations of the two calculated lowest-energy conformers for the (*S*) enantiomer obtained at the SCRF-B3LYP/6-31G(d) level:

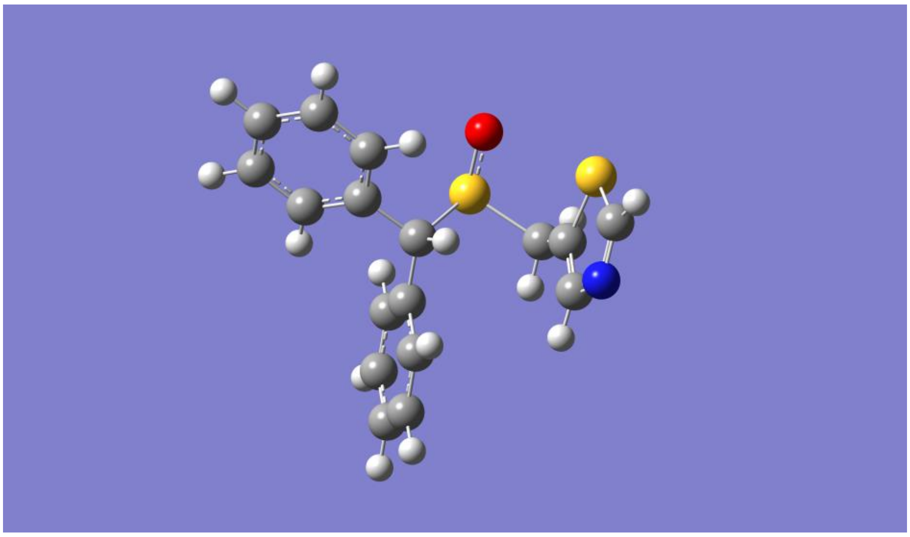

53.6%

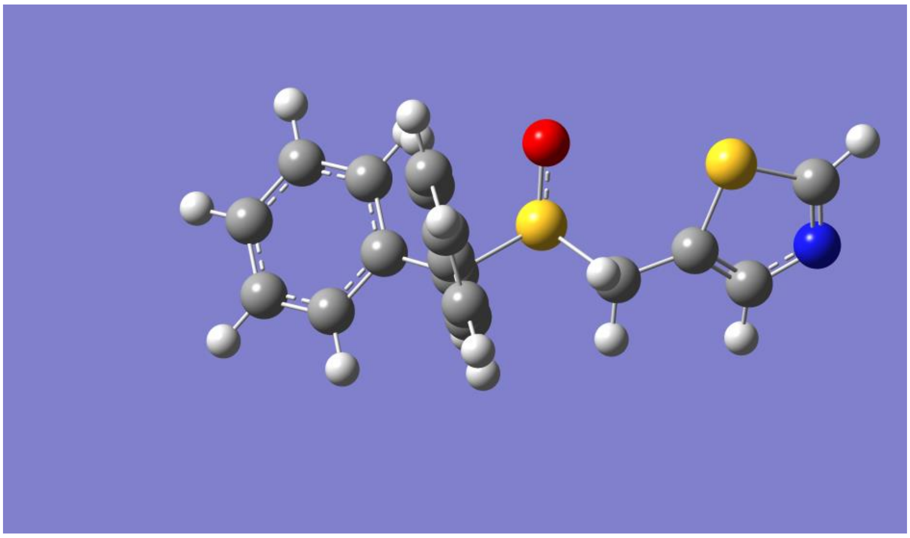

10.7%

9 conformations have a Boltzmann population larger than 1%; 12 conformations have a Boltzmann population larger than 0.1%.

Calculated IR and VCD spectra for the (*S*) enantiomer: B3LYP/6-31G(d)

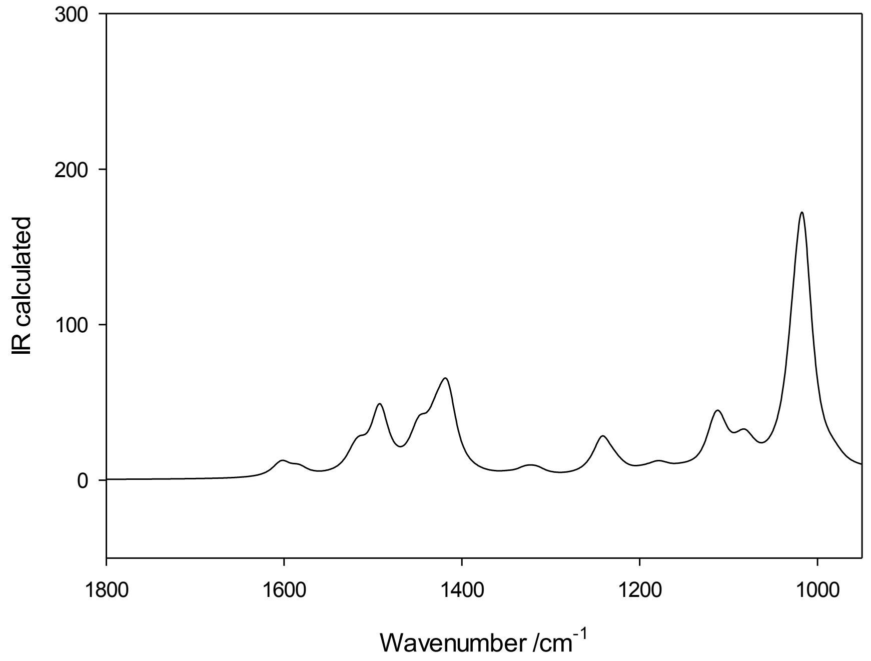

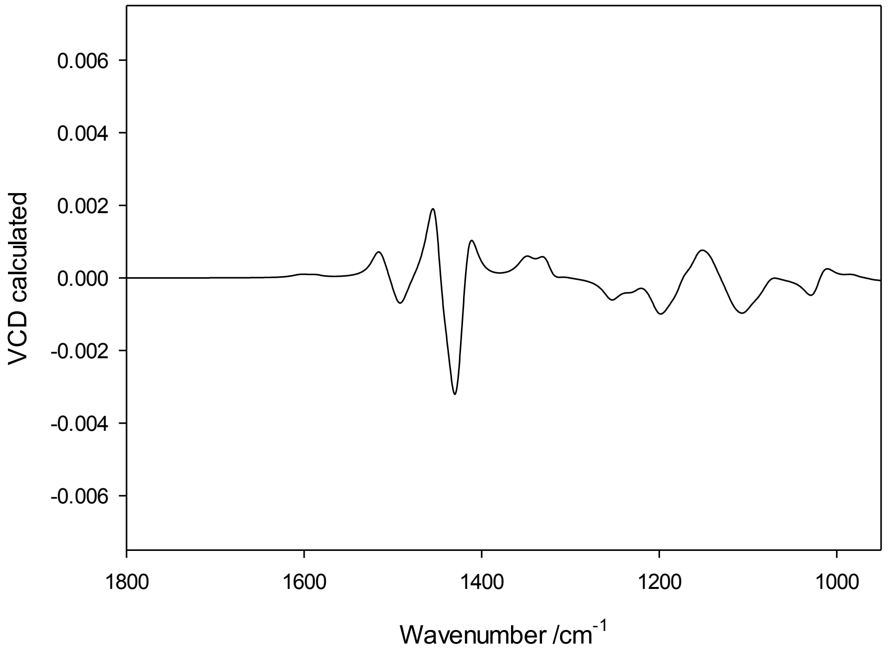

Absolute Configuration Determination Report

Optimized geometries and relative populations of the two calculated lowest-energy conformers for the (*S*) enantiomer obtained at the SCRF-B3PW91/6-31G(d) level:

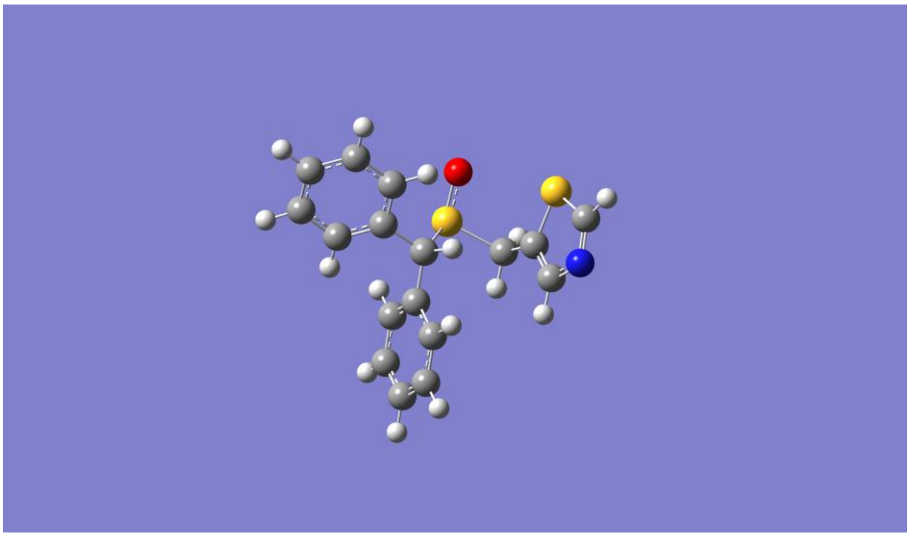

56.8%

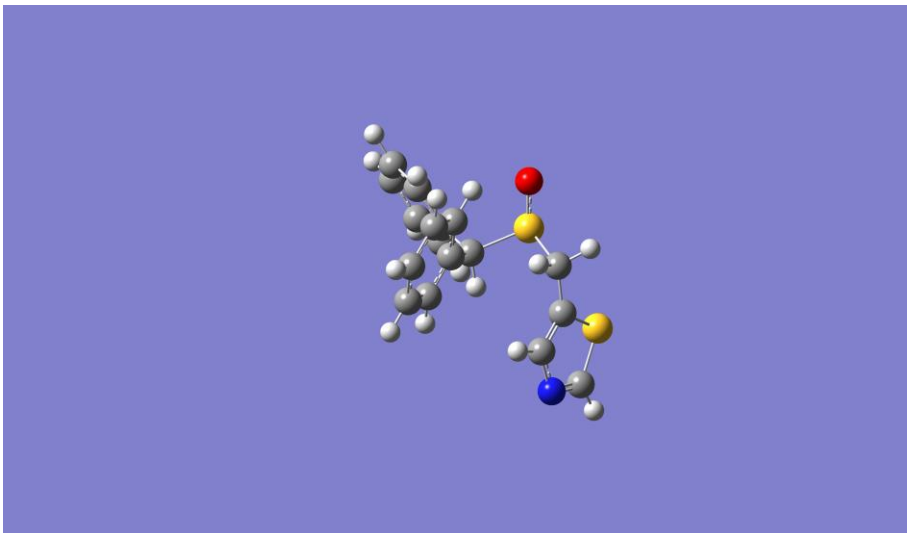

8.7%

9 conformations have a Boltzmann population larger than 1%; 11 conformations have a Boltzmann population larger than 0.1%.

Calculated IR and VCD spectra for the (*R*,*R*) enantiomer: B3PW91/6-31G(d)

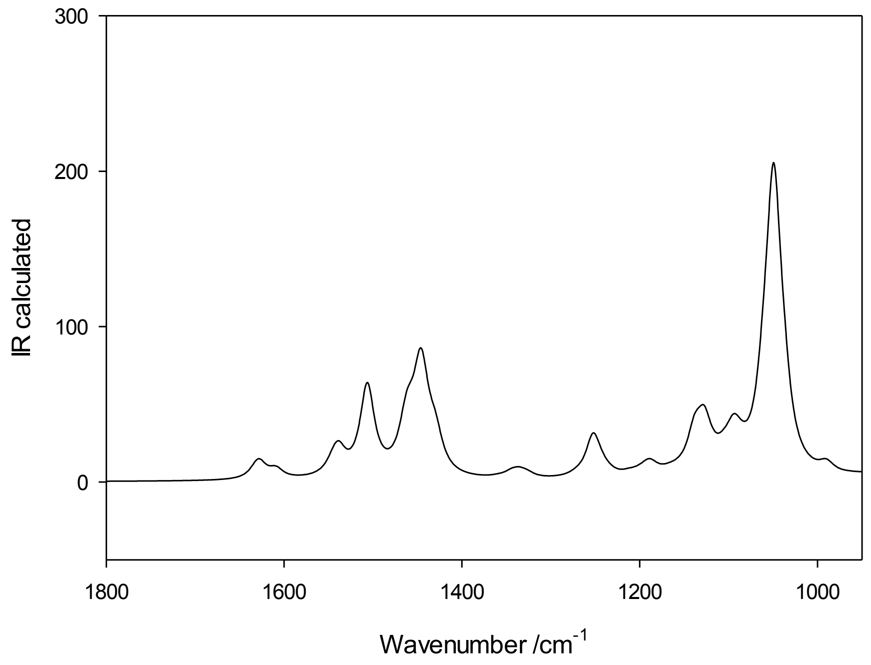

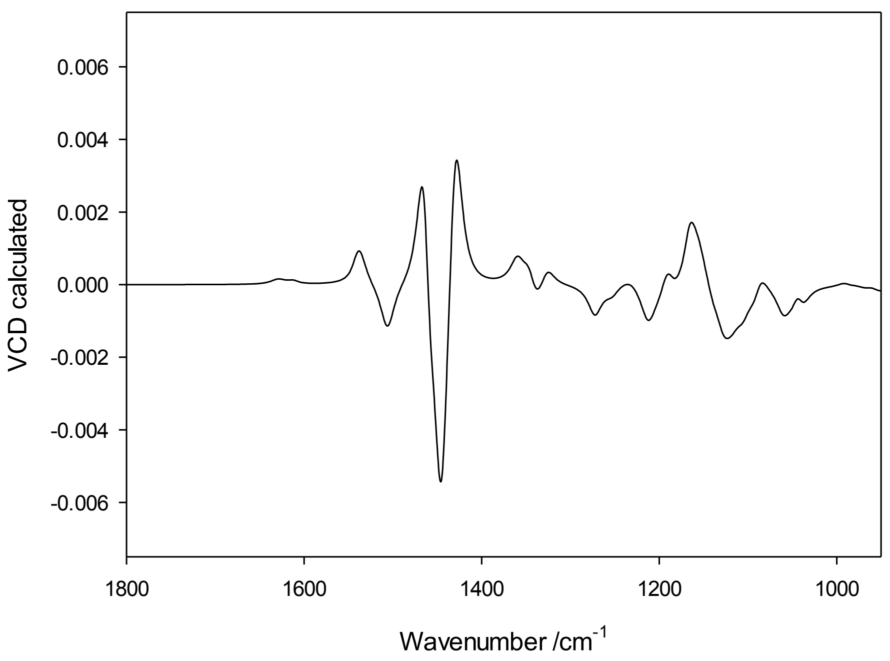

Inspection of the calculated and experimental spectra shows that the B3LYP IR and VCD spectra for the (*S)* enantiomer reproduce the experimental IR and VCD spectra of **S17130**.

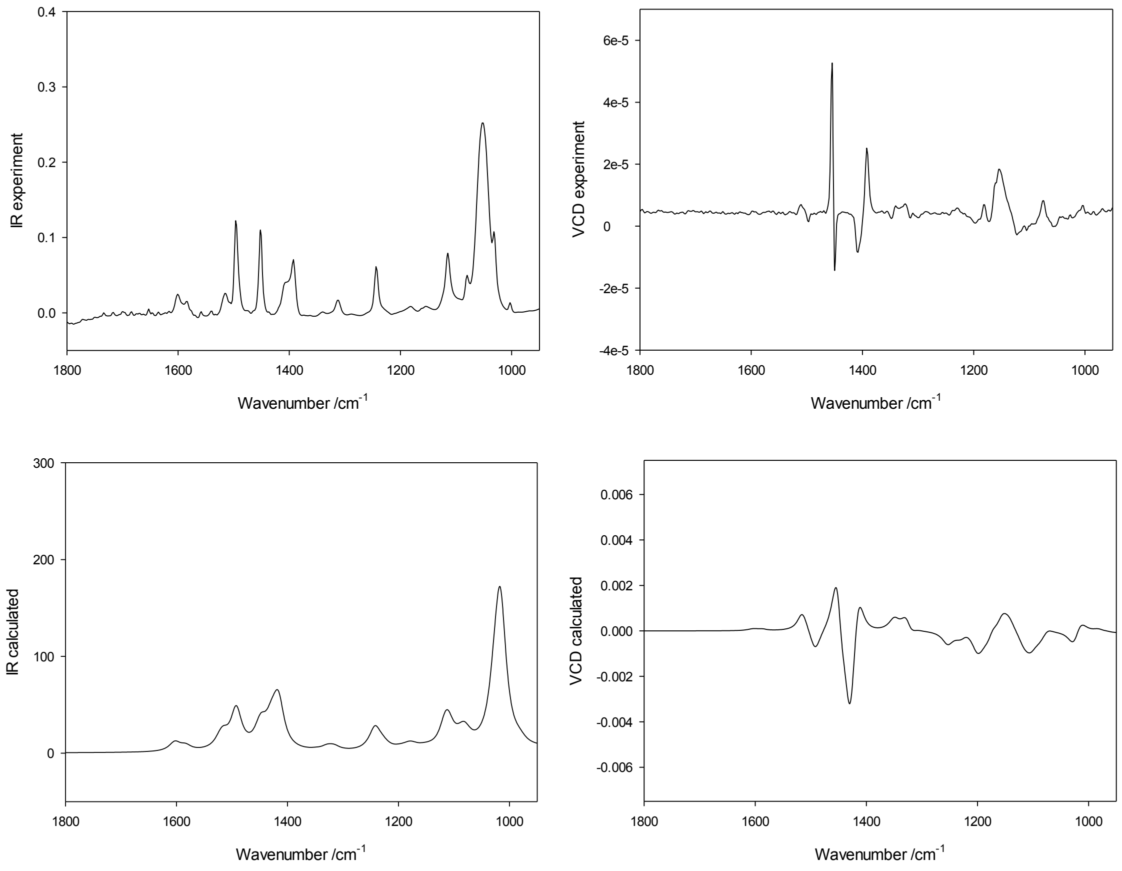

The assignment of the AC of S17130 to (*S*) is confirmed by the neighborhood similarities and confidence levels calculated.

Absolute Configuration Determination Report

Table 1. Numerical comparison describing the similarity in the range of 950-1700 cm^{-1} between the calculated IR and VCD spectra and the experimental IR and VCD spectra of **S17130**.

**S17130 / CE-123A**		***(S)***			
Calculated level	σ^a	$\Sigma_{IR}{}^b$	$\Sigma_{VCD}{}^c$ (%)	Δ^d (%)	CL^e (%)
SCRF-B3LYP	0.967	85.7	66.8	51.4	99

[a]σ: scaling factor. [b]Σ_{IR}: IR similarity, gives the similarity between the calculated and experimental IR spectra. [c]Σ_{VCD}: single VCD similarity, gives the similarity between the calculated and experimental VCD spectra. [d]Δ: enantiomeric similarity index, gives the difference between the values of ∑ for both enantiomers of a given diastereoisomer. [e]CL: confidence level.

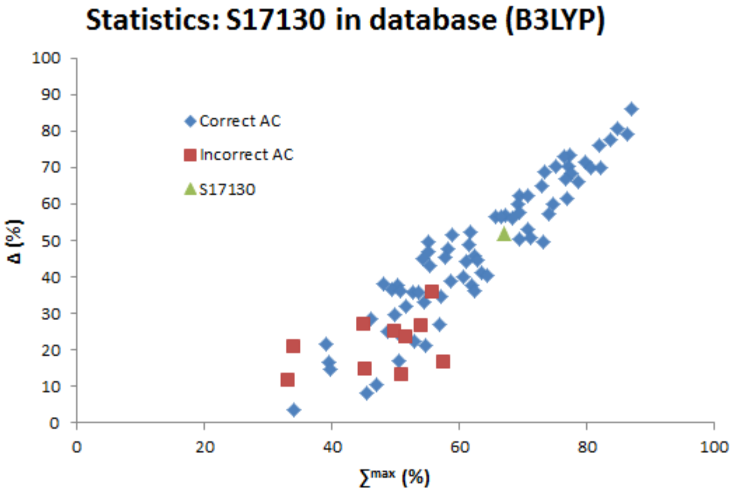

Absolute Configuration Determination Report

Inspection of the calculated and experimental spectra shows that the B3PW91 IR and VCD spectra for the (*S)* enantiomer reproduce the experimental IR and VCD spectra of **S17130**.

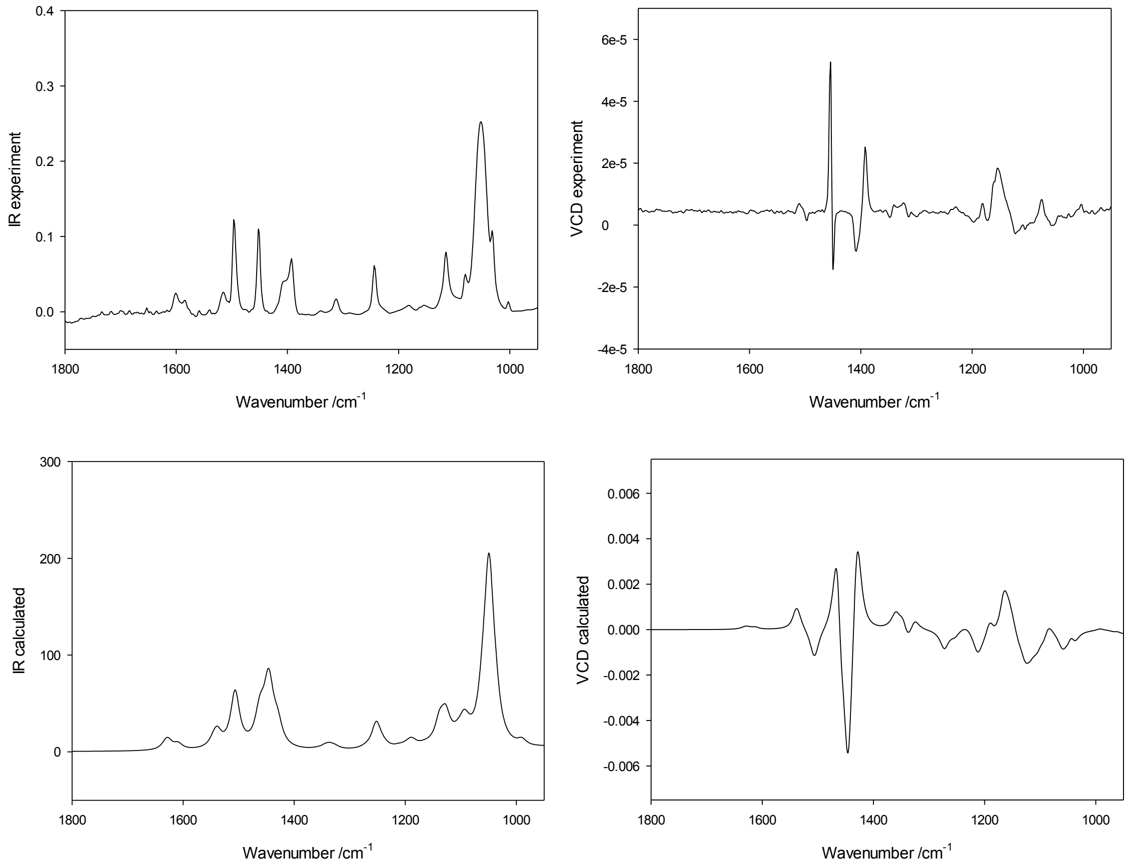

The assignment of the AC of S17130 to (*S*) is confirmed by the neighborhood similarities and confidence levels calculated.

Absolute Configuration Determination Report

Table 2. Numerical comparison describing the similarity in the range of 950-1700 cm^{-1} between the calculated IR and VCD spectra and the experimental IR and VCD spectra of **S17130**.

**S17130 / CE-123 A**		***(S)***			
Calculated level	σ^a	$\Sigma_{IR}{}^b$	$\Sigma_{VCD}{}^c$ (%)	Δ^d (%)	CL^e (%)
SCRF-B3LYP	0.965	85.1	61.1	44.0	99

$^a\sigma$: scaling factor. $^b\Sigma_{IR}$: IR similarity, gives the similarity between the calculated and experimental IR spectra. $^c\Sigma_{VCD}$: single VCD similarity, gives the similarity between the calculated and experimental VCD spectra. $^d\Delta$: enantiomeric similarity index, gives the difference between the values of Σ for both enantiomers of a given diastereoisomer. ^{e}CL: confidence level.

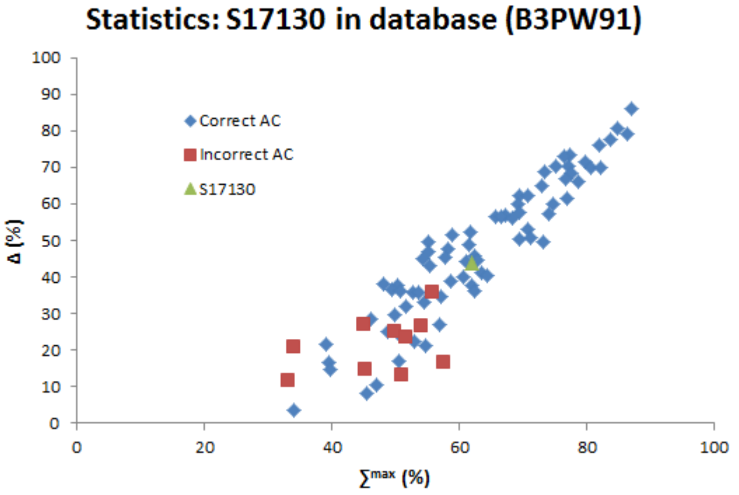

.

Absolute Configuration Determination Report

CONCLUSIONS

Absolute configuration of **S17130** (CE-123 A) was assigned as (*S*) based on the agreement of VCD spectra

Confidence Level: **99 %**

Absolute configuration of **S17131** (CE-123 B) was assigned as (*R*) based on the agreement of VCD spectra.

Confidence Level: **99 %**

GENERAL INFORMATION	
**Customer**	University of Vienna<br>Department of Pharmaceutical Chemistry<br>Predrag Kalaba
**Sales Order Number**	N/A
**Sample code (Our ref.)**	**S17310 / S17311**
**Sample description (Your ref.)**	S17310 = VD-253<br>S17311 = VD-257
**VCD-spectrometer**	ChiralIR-2X w/ Dual*PEM*
**Report prepared by**	Wouter Herrebout
**Report validated by**	Wouter Herrebout
**Report signed by**	NA
**Date**	December, 14th, 2017
**RESULTS**	
Absolute Configuration of **S17310: (*S*)**<br>Absolute Configuration of **S17311 (R)**	No Confidence Level
**MEASUREMENT PARAMETERS**	
**Concentration**	2.5 mg / 100 µL , 4.4 mg / 100 µL
**Solvent**	$CDCl_3$
**Resolution**	4 cm^{-1}
**PEM setting**	1400 cm^{-1}
**Number of scans/Measurement time**	75.000 scans / 24.0 hours
**Sample cell**	BaF_2
**Path length**	100 µm
**CALCULATION DETAILS**	
**Force fields used in MolMec conformational analyses**	NA , calculations performed for S17130
**Number of conformations generated**	NA , calculations performed for S17130
**Methodology and basis set for DFT calculations**	NA , calculations performed for S17130
**Enantiomer used for calculation**	NA , calculations performed for S17130
**Number of conformations used in calculated spectrum**	NA , calculations performed for S17130
**Number of low-energy conformations shown in report**	NA , calculations performed for S17130

S17130 = CE-123A was confidently assigned to S (earlier report , 99% CL)
S17131 = CE-123B was confidently assigned to R (earlier report , 99% CL)

IR and VCD spectra of S17310 / VD-253 and S17311 / VD-257 were recorded. Assignment of the AC was performed by comparing the experimental IR and VCD spectra of S17130 / S17131 and S17310 / S17311.

Due to additional spectral features in the IR and VCD spectra, assigned to unidentified impurities, no confidence levels could be obtained for S17310 / S17311. The characteristic pattern observed in VCD allows the AC of S17310 and S17311 to be confidently assigned.

Molecular Formula: $C_{17}H_{15}NOS_2$

Chemical Structure:

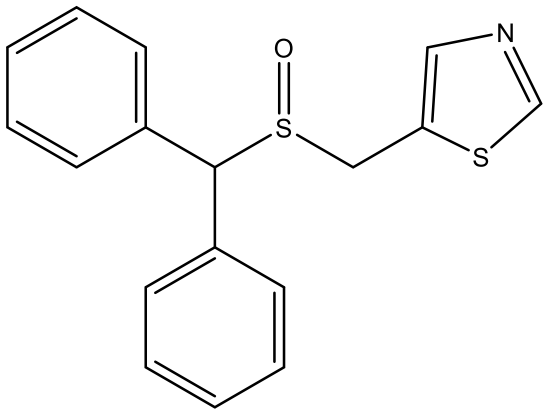

Experimental IR and VCD spectra for S17130 – taken from earlier report

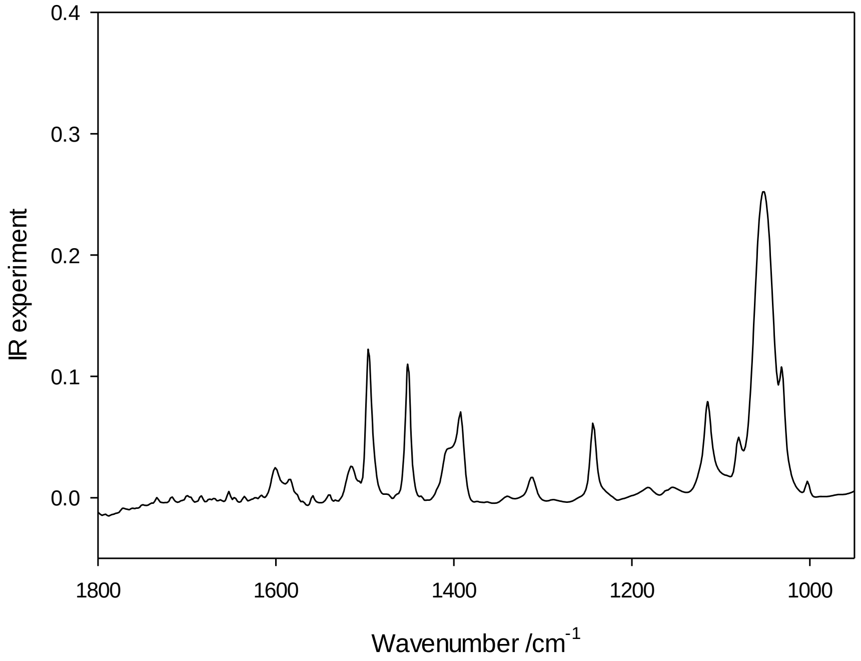

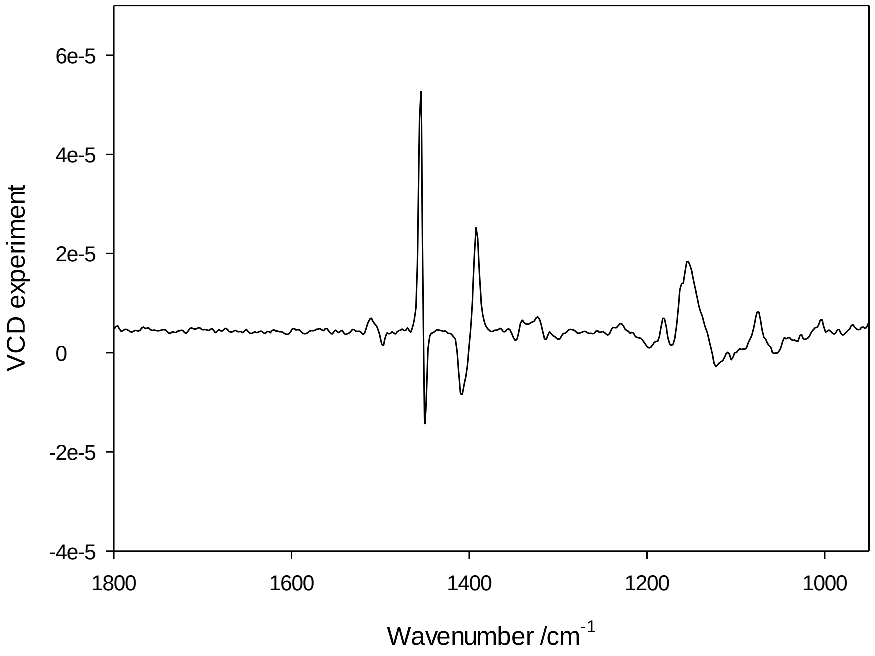

Experimental IR and VCD spectra for S17310 – new data

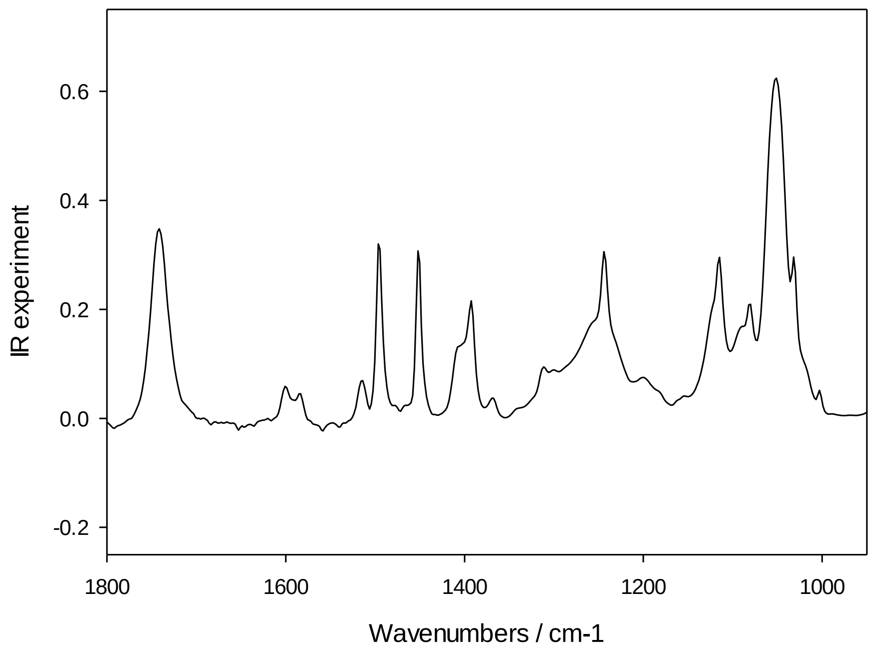

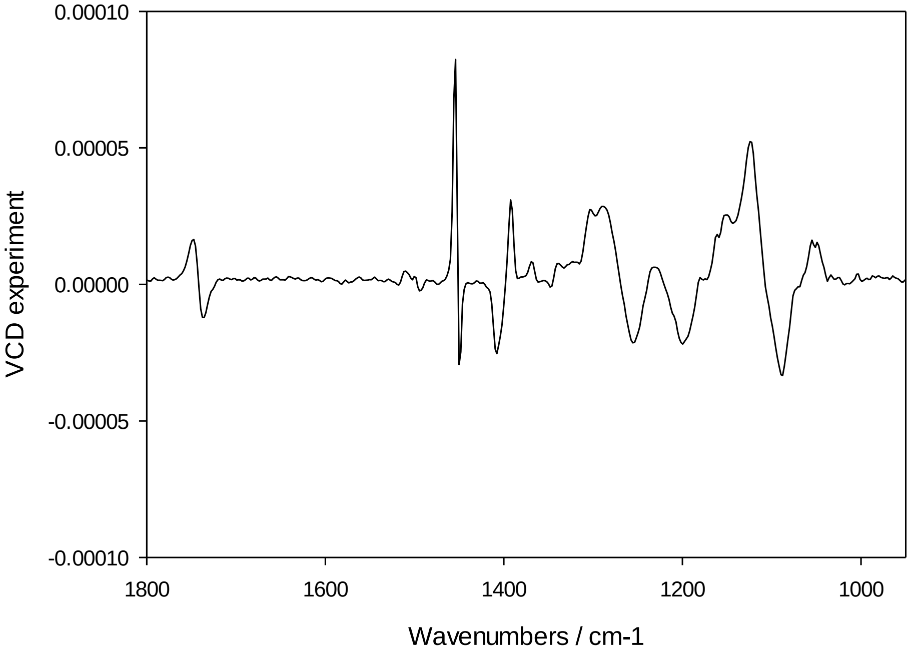

Experimental IR and VCD spectra for S17310 (black) and S17130 (red) – impurities in S17310/311

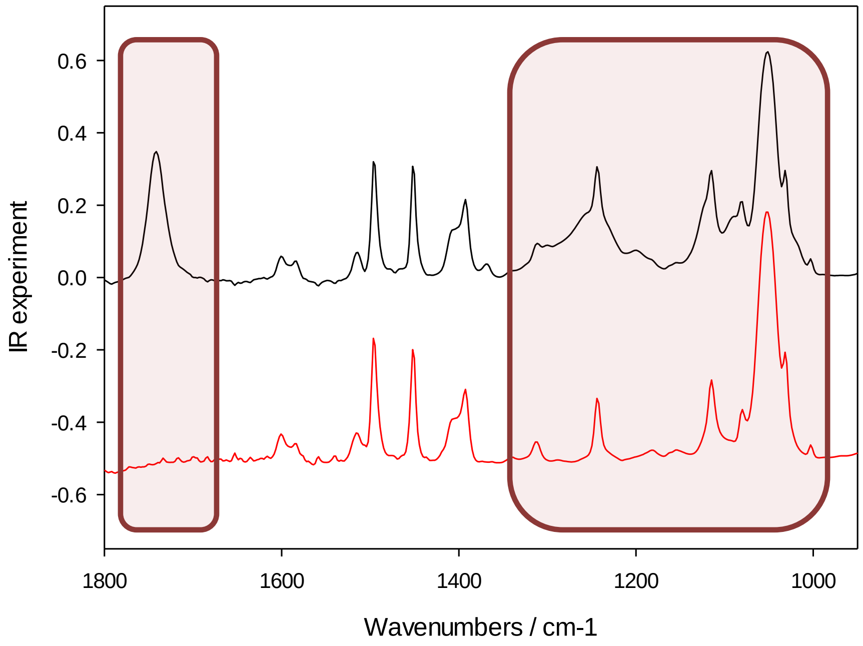

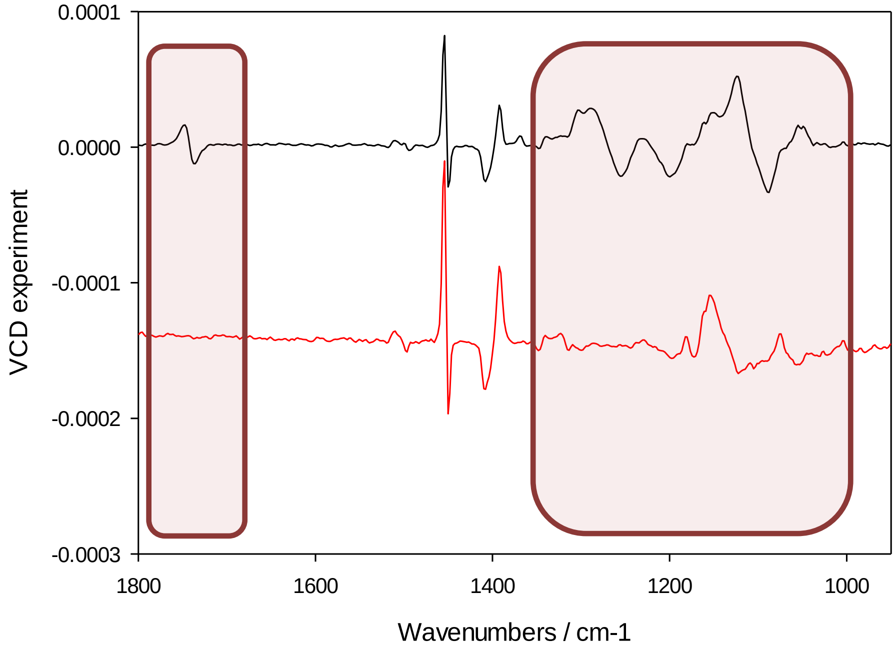

Experimental IR and VCD spectra for S17310 (black) and S17130 (red) – characteristic patterning allowing the AC to be identified

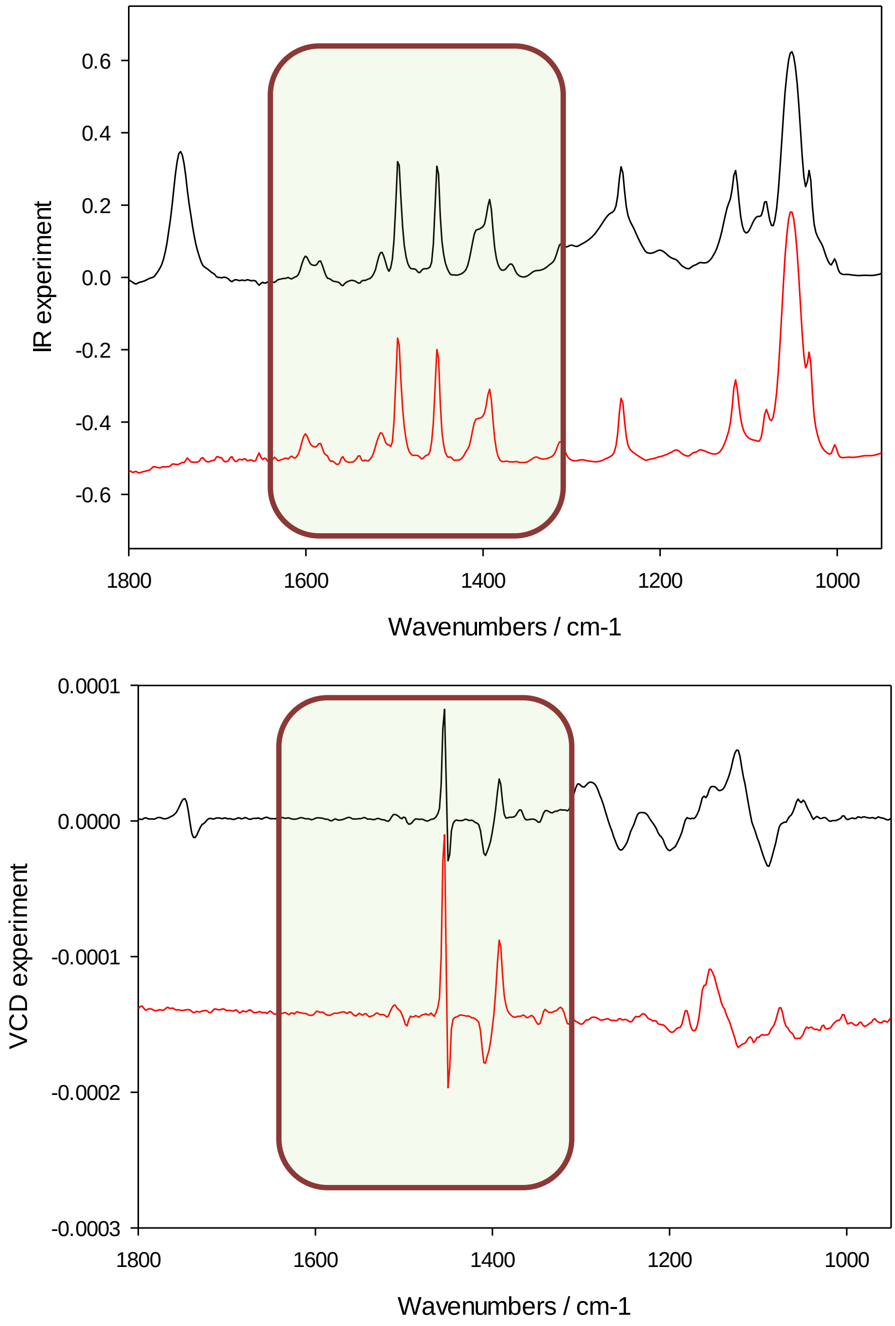

Supplement: Supplementary file 1 [file DataSheet_1.pdf]
